# Practical guidelines for Bayesian phylogenetic inference using Markov Chain Monte Carlo (MCMC)

**DOI:** 10.12688/openreseurope.16679.2

**Published:** 2024-06-28

**Authors:** Joëlle Barido-Sottani, Orlando Schwery, Rachel C. M. Warnock, Chi Zhang, April Marie Wright

**Affiliations:** 1 Institut de Biologie de l’ENS (IBENS), École normale supérieure, CNRS, INSERM, Université PSL, Paris, Île-de-France, 75005, France; 2 Department of Biological Sciences, Southeastern Louisiana University, Hammond, Louisiana, 70402, USA; 3 Department of Biological Sciences, Virginia Polytechnic Institute and State University, Blacksburg, Virginia, 24061, USA; 4 Department of Biological Sciences, Louisiana State University, Baton Rouge, Louisiana, 70803, USA; 5 GeoZentrum Nordbayern, Department of Geography and Geosciences, Friedrich-Alexander Universität Erlangen-Nürnberg, Erlangen, Bavaria, 91054, Germany; 6 Key Laboratory of Vertebrate Evolution and Human Origins, Institute of Vertebrate Paleontology and Paleoanthropology, Chinese Academy of Sciences, Beijing, 100044, China

**Keywords:** Bayesian phylogenetic inference, MCMC, troubleshooting, phylogenetic inference software, fossilized birth-death, total-evidence, BEAST2, MrBayes

## Abstract

Phylogenetic estimation is, and has always been, a complex endeavor. Estimating a phylogenetic tree involves evaluating many possible solutions and possible evolutionary histories that could explain a set of observed data, typically by using a model of evolution. Modern statistical methods involve not just the estimation of a tree, but also solutions to more complex models involving fossil record information and other data sources. Markov Chain Monte Carlo (MCMC) is a leading method for approximating the posterior distribution of parameters in a mathematical model. It is deployed in all Bayesian phylogenetic tree estimation software. While many researchers use MCMC in phylogenetic analyses, interpreting results and diagnosing problems with MCMC remain vexing issues to many biologists. In this manuscript, we will offer an overview of how MCMC is used in Bayesian phylogenetic inference, with a particular emphasis on complex hierarchical models, such as the fossilized birth-death (FBD) model. We will discuss strategies to diagnose common MCMC problems and troubleshoot difficult analyses, in particular convergence issues. We will show how the study design, the choice of models and priors, but also technical features of the inference tools themselves can all be adjusted to obtain the best results. Finally, we will also discuss the unique challenges created by the incorporation of fossil information in phylogenetic inference, and present tips to address them.

## 1 Introduction to MCMC

Phylogenetics has always had a fundamental problem. For any reasonable number of taxa, the number of possible topologies that could connect them quickly scales to be larger than the number of stars in the sky. It is intractable to evaluate all of them. And yet, increased taxon sampling is crucial to phylogenetic accuracy ( Heath *et al.*, 2008; Hillis *et al.*, 2003; Rannala *et al.*, 1998). One computational technique revolutionized our ability to enumerate and evaluate solutions in a Bayesian framework. That technique is Markov Chain Monte Carlo (MCMC). Though others exist, such as sequential Monte Carlo (SMC, Bouchard-Côté *et al.*, 2014) or Hamiltonian Monte Carlo (HMC, Dinh *et al.*, 2017), MCMC remains the most commonly applied technique for this type of evaluation.

To understand MCMC, we must first take a step back and understand mathematical models. In a model, parameters describe what the researcher views as important facets of the process that generated our observed data. For example, in a phylogenetic model of molecular evolution, there may be a parameter governing the rate at which transitions have occurred and a different one governing the rate at which transversions have occurred to generate an observed multiple sequence alignment. In most models, parameters are usually random (also called stochastic) variables, meaning the value of a parameter is derived from an event with some element of randomness, such as a draw from a probability distribution or a coin flip. In the models we consider, most of the parameters are continuous, meaning they can take any value within their reasonable ranges. The uncertainty of a continuous parameter is described by a probability density function (e.g., a uniform or an exponential distribution), and the probability within a range of values is the area under the curve of the probability density function. For discrete parameters, such as the tree topology, each possible value of the parameter has a probability. We collectively use “probability distribution” for both discrete and continuous parameters.

In a maximum likelihood (ML) estimation, we try to find the values for all our parameters that maximize the likelihood of the parameters given our data. ML solutions can be efficiently estimated through a number of mathematical techniques. In a Bayesian estimation, we estimate a distribution of the parameters that are plausible under our model given the data. In addition, Bayesian inferences integrate prior distributions, which describe our prior knowledge and understanding about the model and parameters, before having looked at the data. Bayesian inference thus offers a more complete picture of the results, integrating uncertainty in the results as well as existing information from previous studies. However, it is also more complex, because for many real world scenarios, the true distribution of plausible parameters cannot be calculated directly.

MCMC algorithms allow us to find the set of plausible solutions of a Bayesian inference, that is, an estimation of the posterior distribution of the parameters. The algorithm for MCMC sampling most frequently employed in phylogenetic studies is known as the Metropolis-Hastings (MH) algorithm, though others exist. The general way it works is that a starting set of values is proposed for the parameters. This set is then scored according to some criterion. Then, one or more model parameters are perturbed, or changed. This could be a simple change, like making a number a little bigger. In the case of phylogenetics, we often need to use more complex moves to propose new values for non-numeric objects like clades and trees (this will be described in **Moves/Operators**). “Monte Carlo” is the operative term here. The city Monte Carlo is famous for its casinos and games of chance. This means that we perturb the parameters pseudorandomly (at random within some set of conditions). The new value or set of values proposed will be re-scored according to the evaluation criterion. If it is better, this solution becomes the new current solution from which new moves will be performed. If the score of the proposed value is worse than the current, we still have a chance to accept it − broadly explore the entire parameter space and lower our chances of staying stuck in a local optimum. The probability of accepting the proposal depends on the difference of evaluation values between the new and current scores, so that much worse proposals mostly will be discarded. The “Markov” chain part of the name comes from this being a Markov process, meaning a memoryless process. That is, the new state proposed depends only on the current state, not on the previous states. If a parameter value (or a region of values) has a high score, it will be visited many times in an analysis. In Bayesian phylogenetics, MCMC samples parameter values proportional to their posterior probability. Therefore, if a set of values for model parameters give a good solution according to the evaluation criterion, the MCMC will tend to sample those values and other similar values often. MCMC chains are ergodic, meaning that any state of the chain is theoretically reachable from any other state. To fulfil this criterion of ergodicity, we place moves on every parameter in our model, thereby enabling the algorithm to sample thoroughly. Finally, MCMC is sometimes referred to as a “simulation” algorithm, which can be confusing. The reason for this is that we are not changing the underlying data, but proposing new values for model parameters to try and improve the fit of the model to the data. Often, this involves drawing parameter values out of a distribution, or scaling parameters in our model − both of these are forms of simulating new values.

Much like Bayesian analysis itself, MCMC was not developed to deal with phylogenetics, or even biological data directly. Those applications came later. Invented in the early 1950’s, MCMC was originally used in physics to describe equilibrium between the liquid and gas phases of a chemical ( Metropolis *et al.*, 1953). In this case, all the values being perturbed in the model are numerical, which is not always the case with phylogenetics. From a humble beginning of trying to model a simple physical system, the MH MCMC algorithm drew the attention of statisticians, who popularized its use across nearly every quantitative discipline. In the following sections, we will discuss how MCMC works for phylogenetic inferences, how to troubleshoot an MCMC inference, and some tips and tricks for MCMC success.

## 2 MCMC inference applied to phylogenetics

### 2.1 The basics

**Figure 1.**
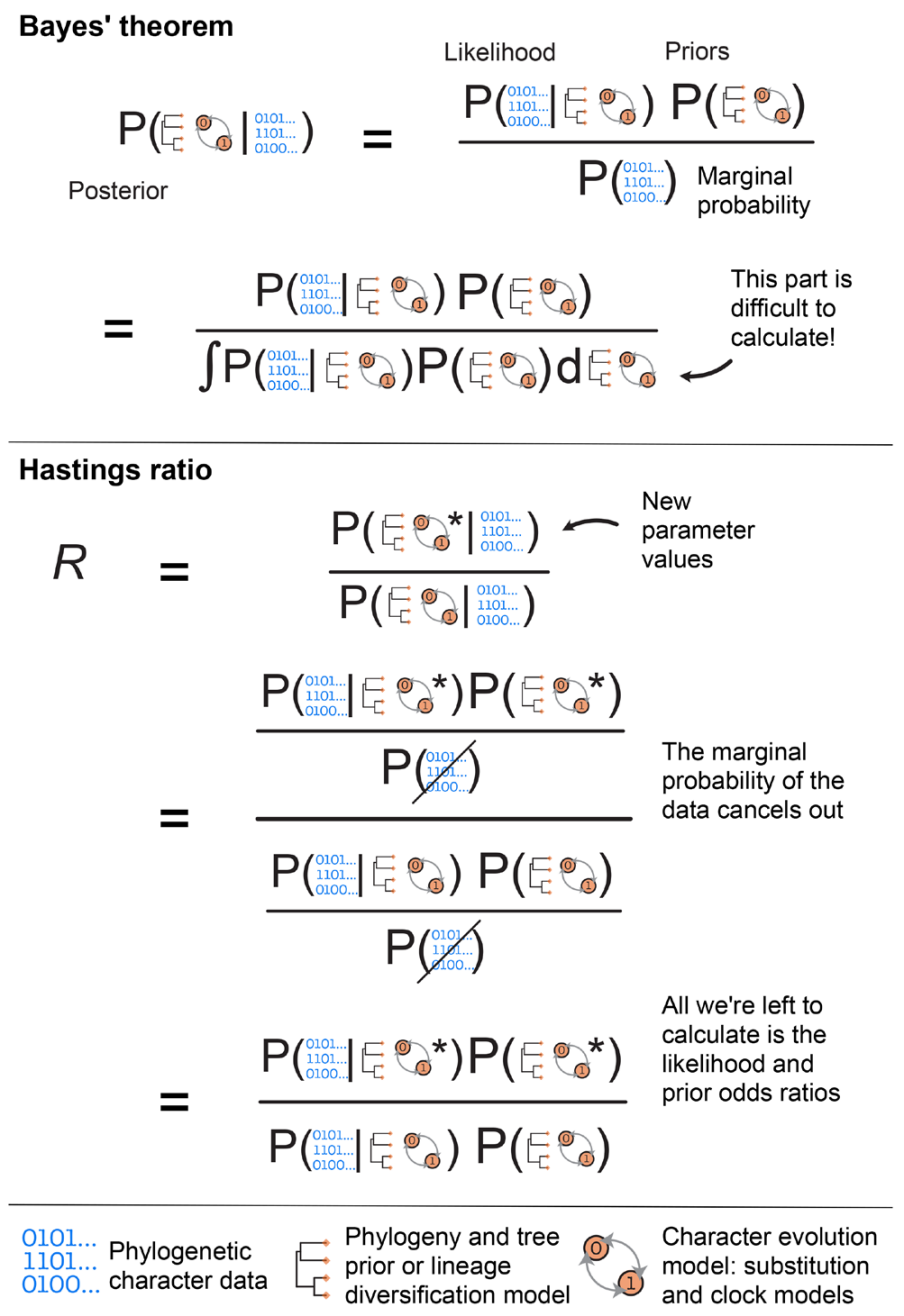
The top panel shows Bayes’ theorem and the relationship between the posterior, likelihood, priors and the marginal probability of the data. The bottom of this panel shows an alternative way of writing the marginal probability, which illustrates more explicitly why the marginal probability is difficult to calculate. During MCMC we sample new parameter values at each step and compare their posterior probability to the previous set of values using the Hastings or posterior odds ratio, R. The middle panel shows the Hastings ratio, and illustrates that since the marginal probability cancels out, we avoid having to calculate it during MCMC. The bottom panel provides a legend for the main data and model components used in Bayesian phylogenetic inference. The observed data used to infer phylogenies typically comprise discrete morphological characters (shown here) or molecular sequences. During inference the tree topology and branch lengths are sampled from a prior distribution that depends on the type of inference − in particular, if the tree is dated we typically use lineage diversification models, such as birth-death processes. The character evolution model will always include the substitution process and will additionally include the clock model if we aim to infer a dated phylogeny.

Before we can understand MCMC in-depth, we need to discuss some basic information about Bayesian inference. Bayesian inference refers to a statistical framework for evaluating the fit of models and parameters to the observed data, based on a quantity called the posterior distribution. The posterior distribution is calculated from three quantities: **the prior distribution, the likelihood,** and **the marginal probability** of the data. Bayes’ Theorem is shown in Figure 1 and shows the relationship between these three quantities. We will first describe them and how they fit together, then move on to how MCMC is used in their calculation.

***2.1.1 The likelihood.*** The likelihood of the models and parameter values describes how probable the observed data is given those models and values, i.e., how likely it is that those models and values represent the true generating process. If we are only concerned with the highest likelihood given the data, we usually do not need MCMC inference. Many phylogenetic tools can perform maximum likelihood (ML) inference, which finds a set of values for the model parameters that maximize the probability of observing the data.

In a phylogenetic context, the data will usually be our observed molecular sequence alignment and/or morphological character matrix. The model will typically describe the process of evolution that generated these data. In a Bayesian phylogenetic inference, the calculation of the likelihood will include a substitution model, which describes the relative rate of change from one character to another as well as the frequencies of each character state, and a clock model, which describes the overall rate of change through time and across the tree. For example, the simplest substitution models are the Jukes-Cantor model (molecular data; Jukes & Cantor, 1969) and the Mk model (morphological data; Lewis, 2001). These models assume that one parameter describes the process of sequence evolution generating the data, and as a result these models are often referred to as ‘all-rates-equal models’. This one parameter is a rate of change between different molecular or morphological character states. Many substitution models (such as the Kimura 2-parameter model ( Kimura, 1980), the Felsenstein 1981 model ( Felsenstein, 1981), the Hasegawa-Kishino-Yano model ( Hasegawa *et al.*, 1985), and the General Time-Reversible model ( Tavaré, 1986)) are more complex, and reflect different assumptions regarding the hypothesized process of sequence change and evolution.

In a Bayesian analysis, the likelihood is one component of the three parts of Bayes’ Theorem ( Figure 1). It is calculated at each step in the MCMC analysis and is an important part used to estimate the posterior probability distribution given the data. The other important part is the prior.

***2.1.2 The prior.*** A crucial analytical difference between a maximum likelihood method and a Bayesian one is the presence of a prior. The term prior means that the distribution of the parameters reflects one’s belief before observing the data. Each parameter in a Bayesian analysis has a prior probability distribution. For instance, we can set an exponential distribution on a given rate parameter. Under this prior, a rate that is very high is believed to be less likely than one that is very short. This means that rates are expected to be fairly low, but we still allow the possibility that they could be higher.

In Bayes’ Theorem, the prior and the likelihood are multiplied together, thus proposed parameter values are evaluated based on both the likelihood and the prior distribution. Therefore, if we expected a solution to be unlikely and thus specified a low prior probability for it, that low prior will lower the posterior when being multiplied with the likelihood. Importantly however, if against our expectations, this solution is strongly supported by the data, the resulting high likelihood may overcome the effect of the low prior and still lead to high posterior support. This is how we can still find solutions which are different from our initial expectations, if the data suggest them. But this also highlights why we have to be careful not to specify priors that are too strict (i.e., that specify the prior probability of reasonable solutions to be 0), and prevent the MCMC from exploring the parameter space the data would favour. In addition, we note that in the context of Bayesian phylogenetics not all parameters of interest will be fully identifiable (e.g., rates and times, see section Inferring dated trees and incorporating fossils). This means that the priors we use in these analyses should be carefully considered and justified.

***2.1.3 The marginal probability.*** The marginal probability of the data, also called the marginal likelihood, is the probability of the data without considering any particular model parameters, but conditioned on the models themselves and the constraints of the prior. Thus it gives the overall likelihood of the chosen model over all possible parameter values. This is usually the most challenging part of the calculation, as calculating the absolute probability of the data averaging over all possible values of the model parameters is not computationally feasible in many cases. In a typical Bayesian phylogenetic inference, we avoid calculating the marginal probability using the MH algorithm ( Figure 1, explained below). However, if we can calculate the marginal probability, it allows us to perform model selection. The marginal probability is typically computed by sampling many different solutions and averaging them for their probability. Different estimation methods have been developed to approximate the marginal likelihood, such as path sampling, which includes the widely used stepping-stone method ( Baele *et al.*, 2012) or nested sampling ( Russel *et al.*, 2018). Overall, these methods remain expensive. Note that prior specificity matters for model selection, and overly-vague priors can cause issues for model selection and parameter estimation, even if the true parameter is included ( Zwickl & Holder, 2004).

***2.1.4 The posterior.*** The posterior distribution (posterior for short) is the probability distribution of the model parameters given the data. The posterior can change if the underlying data, model, or prior distributions change.

As explained in the previous section, the theoretical posterior (i.e., the exact, ‘true’ solution) is almost always impossible to calculate directly. Hence we use MCMC to sample a set of parameter values that can approximate the posterior distribution of the parameters (usually called the posterior sample or MCMC sample), using the machinery introduced in section **Implementation of MCMC in phylogenetic inference software**. MCMC is key in Bayesian computation, as it allows us to sample from the posterior distribution. MCMC can even evaluate different potential model solutions through reversible-jump MCMC, which allows the chain to move between different models (and their associated parameter spaces) during the inference. It is important to note that the result of an MCMC inference is the full posterior sample and the distribution of solutions. The distribution not only quantifies a point estimate (such as the mean or median), but also the uncertainty associated with it. In this sense, we typically evaluate the whole set of the posterior sample rather than an individual point from the sample.

***2.1.5 The Metropolis-Hastings algorithm.*** The MH algorithm enables us to sample from the posterior without having to calculate the marginal probability of the data. The trick is that we use the *posterior odds ratio* or *Hastings ratio* ( *R*) to evaluate how the chain proceeds, i.e., whether we accept the newly proposed values at each iteration. More specifically, this is the ratio of the posterior probabilities for the new values versus the current (parent) values. Since the marginal probability is the same in both cases, it cancels out when we calculate the ratio, meaning we only need to calculate the likelihood and the prior probability for each set of values, shown in Figure 1.

**Figure 2.**
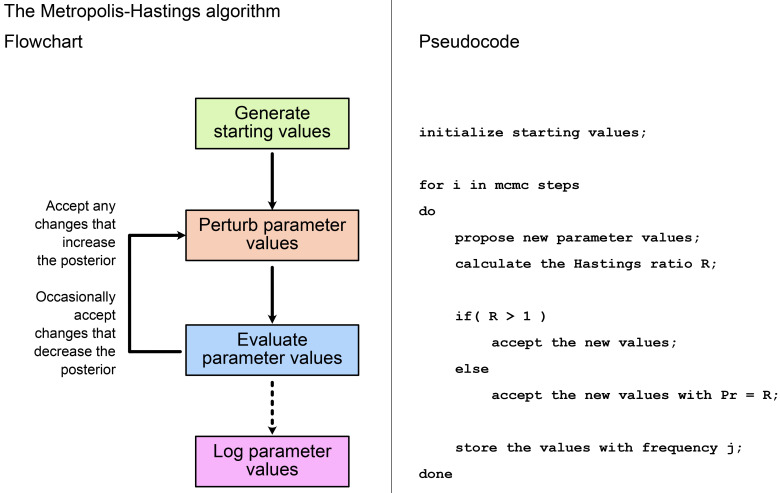
Flowchart and pseudocode showing the main steps in the Metropolis-Hastings algorithm. See Figure 1 for a full description of the Hastings ratio.

Figure 2 shows the main steps in the MH algorithm. As described in the **Introduction**, we first propose an initial set of values for all model parameters, including the topology (if estimating), and record the likelihood and prior probability associated with these. In each subsequent step, at least one model parameter is perturbed, and again we record the likelihood and prior probability. We evaluate the new values using the Hastings ratio. If *R >* 1, i.e., the new values improve the posterior, these are always accepted and become the updated current values from which the chain proceeds. If *R* < 1, the new values are only accepted with probability = *R*. This means, if the posterior associated with the new values is much lower, there is only a small chance of them being accepted. If the new values are not accepted, then the current values remain unchanged. By following these rules, the algorithm spends most of its time in regions of the parameter space with the highest posterior probability. We repeat the process of perturbation and evaluation until we have a sufficient number of MCMC samples to approximate the posterior. Several online tools demonstrate how this process works on different shapes of posterior space, such as the MCMC robot ( Lewis, 2024) or the MCMC demo app ( Feng, 2024). We do not need to store the values at every iteration, since consecutive values will be strongly correlated with each other. The best sampling frequency will depend on the size of the moves between iterations, and also on the specific software implementation used. For computational and memory reasons, we usually aim to record between 1,000 and 10,000 posterior samples. We discuss how to assess whether these samples are a good representation of the posterior later in this article.

***2.1.6 The posterior sample.*** The posterior sample is a set of plausible solutions for a given dataset, derived through MCMC analysis. The posterior sample is composed of all recorded steps, which is a subsample of the steps visited by the inference. The distribution of solutions in the posterior sample is, itself, meaningful. Each entry sample in our posterior sample will have a posterior probability, and solutions will be sampled proportional to their posterior. A solution with a good posterior probability will be visited many times, whereas a solution with a poor one will be seldom seen in the posterior sample. How often a solution is sampled out of the total number of samples is often considered a measure of support. For example, a common measure of support for clades on a tree is the posterior probability, which corresponds to the proportion of trees in the posterior sample which contain that specific subclade. A nice property of the posterior sample is that it not only provides the joint estimation of all the parameters, but also individual estimations for all the parameters. Indeed, taking only the sampled values for a specific parameter provides the *marginal posterior distribution* of this parameter, which allows us to estimate values for that parameter while integrating over all possible values of the other parameters. This means that all parameters of the inference can be analyzed independently.

### 2.2 Implementation of MCMC in phylogenetic inference software

***2.2.1 Unrooted versus rooted trees.*** Phylogenetic trees exist in multiple forms. The first important distinction is between unrooted trees, which simply describe the evolutionary relationships of all the samples, and rooted trees, which include an explicit origin or starting point for the evolutionary process. Another important feature of phylogenies is whether they are dated, i.e., whether their branch lengths are expressed in units of genetic/morphological distance or in units of time. Estimating a dated phylogeny requires a model for the molecular or morphological clock, a prior on the phylogeny, as well as time information to calibrate the tree. This information can be provided directly through the data, if the dataset includes samples from multiple points in time, such as fossil specimens. Alternatively, the information can be provided as node calibrations, which provide information directly on the ages of specific nodes of the phylogeny. The prior on the phylogeny can be provided by a model of lineage diversification (usually a birth-death process or a coalescent process ( Lambert & Stadler, 2013)), or simply use a uniform distribution on tree topologies ( Ronquist *et al.*, 2012).

Dated trees are naturally rooted, as the earliest time point of the tree is obviously the origin of the process. Undated trees can also be rooted, by using one or more outgroup samples. In this case, the root is placed at the point in the tree where these outgroups diverge from the main clade of study.

A much wider array of biological questions can be addressed using dated phylogenetic trees (e.g., diversification rate estimation or the application of phylogenetic comparative methods), but inferring dated trees increases the complexity of the analysis, making MCMC inference more challenging. Thus we mainly target this article at analyses which include a molecular or morphological clock as well as time information, although many of the tips detailed here are equally applicable to undated phylogenies.

***2.2.2 General frameworks.*** Bayesian phylogenetic inference is often implemented in large software frameworks which group together many different models. In this paper, we chose to focus on BEAST2 ( Bouckaert *et al.*, 2014), MrBayes ( Huelsenbeck & Ronquist, 2001) and RevBayes ( Höhna *et al.*, 2016) as our examples. These frameworks are generally designed to be modular, with each component of the analysis operating independently from the others. This means that any component, e.g., the substitution model, can be modified easily or extended without having to change anything else. It also means that core parts of the MCMC inference, for instance the MCMC algorithm itself, do not have to be reimplemented when a new model or a new type of data is introduced.

**Figure 3.**
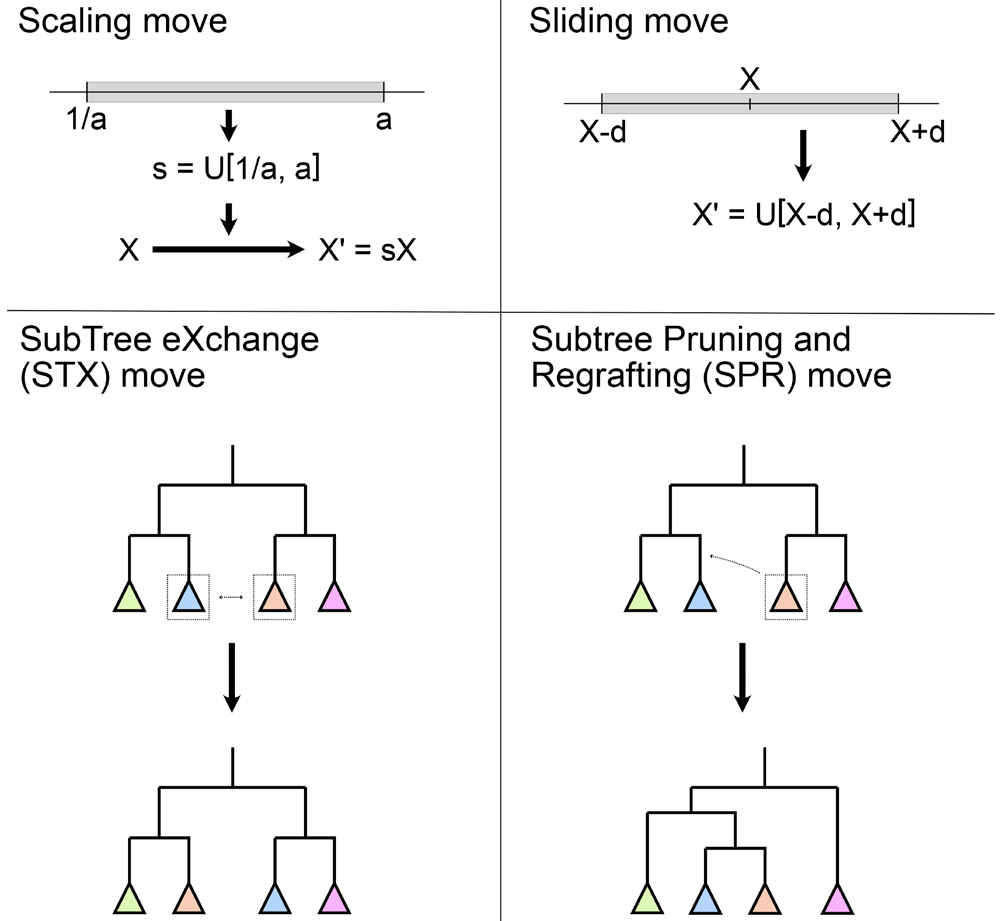
Examples of some common moves used in Bayesian phylogenetic inference. Scaling and sliding moves operate on a numerical parameter (X), such as the molecular clock rate, the speciation rate or the age of a fossil. Subtree exchange (STX) and subtree pruning and regrafting (SPR) moves operate on the tree topology.

***2.2.3 Moves/operators.*** As introduced earlier, MCMC inference relies on moving step by step through the parameter space and recording the state of the model parameters periodically. The recorded parameter states are the MCMC sample. Thus, any MCMC inference software must implement code to advance the chain. Since this code is not part of the model and usually does not depend on the specific choice of analysis, it is generally placed in separate components. In phylogenetic inference tools, these components can be called *proposals*, *moves*, or *operators*, but they all perform the same function in the inference. Examples of some of these moves are shown in Figure 3.

Moves are composed of three elements: first is the parameter or parameters they act on, meaning the parameters they change. Some moves only operate on one parameter at a time, while more complex moves can act on several (correlated) parameters at the same time. For instance, the up-down operator in BEAST2 will scale both the branch lengths of the tree, and the clock rate simultaneously. The second component of a move is the algorithm used to change the value of the parameter(s). These range from basic operations, such as proposing a new value using a sliding window centered on the current value, or scaling the current value of the parameter by a given factor, to much more complex ones such as those used to modify the tree. Finally, the third component of a move is its weight, which determines the frequency with which it will be used during the actual inference. A move with higher weight will be used more often, which should in principle lead to the corresponding parameters moving more often, and in turn provide more accurate estimates for these parameters. It should be noted that MCMC implementations differ in how weight is applied. Some attempt one move per step in the MCMC chain (e.g., BEAST2 and MrBayes), meaning only one parameter changes at a time and the weights represent the probability for any particular one to be chosen. Others move a whole set of parameters at each step (e.g., RevBayes), with the weights representing how many times a move is attempted for a particular parameter during each step. This is also the reason why the number of generations that were run for a given analysis cannot always be compared directly between implementations, as one ‘iteration’ or ‘generation’ of the chain may actually imply different numbers of actual parameter moves (or attempted moves).

However, the efficiency of the MCMC inference also depends on the acceptance proportion of each move, i.e., the percentage of times that the move is accepted during the MCMC run. A move with a very low acceptance rate will have little impact on the overall inference, even if its weight is high. On the other hand, a very high acceptance rate can indicate that the move is proposing new values that are too close to the original values, which slows down the inference and increases the number of steps needed to properly explore the parameter space. For MCMC moves operating on a continuous numerical parameter, such as a branch length or evolutionary rate, the highest efficiency is typically achieved when the acceptance proportion is around 0.2 to 0.4 ( Yang, 2014, section 7.3–7.4). Software implementations such as MrBayes, BEAST2, and RevBayes, typically provide an automatic tuning mechanism, which is enabled by default and adjusts each operator’s configuration to reach the target acceptance proportion, say 0.3. For topological moves or moves which jump between different models, the efficiency is different from that of the more simple moves, and essentially depends on the specific design of the proposal algorithm. As a result, general users cannot easily optimize these moves. Good tree proposals are still under development, there is no perfect one to rule them all. In practice, using a collection of moves that make both big and small topological changes is advised. For example, MrBayes combines a Nearest Neighbour Interchange move (NNI, a narrower implementation of STX) and two SPR variants (see Figure 3) to update the tree. Tree moves should usually have much higher weights than the simple moves, as the tree space is tremendous.

The array of available moves in phylogenetic inference can be daunting for users. Luckily, most inference software proposes a default setup for standard analyses, which includes reasonable moves covering all parameters of the analysis. The default selection of moves usually leads to satisfying results for most standard analyses, however, they certainly cannot fit all circumstances. We will see in later sections how to diagnose and adjust the move setup to help with misbehaving analyses.

## 3 Challenges of phylogenetic MCMC inference

As mentioned in the section **Introduction to MCMC**, MCMC was not developed for use in phylogenetics. It was developed for use with physics models, which usually have solely numerical components, often with many observations relative to the number of parameters. The use of MCMC for phylogenetics raises a new set of issues. In a phylogenetic analysis, we are often principally concerned with estimating a non-numeric parameter: the phylogeny itself! We also often have high-dimensionality models, which contain a large number of parameters. Biology is complex, and we expect the generating model to be complex as well. This can raise serious performance issues for our MCMC inference, either when exploring the tree space or when calculating the posterior values. We will now dive into some of these issues, and how MCMC inference has been adapted to work with phylogenetic trees and data.

### 3.1 Non-numeric data

As explained in the previous section, MCMC relies on perturbing our model parameters through *moves.* For numerical parameters, it is often very easy to perform a move. For example, slide moves simply change the numeric value of a parameter within a window of a given size. Scale moves make values a bit bigger or smaller, while ensuring negative numbers stay negative and positive ones stay positive. For more complex cases, such as simplexes (sets of values that must sum to a number, typically one − for instance nucleotide frequencies in a substitution model) or ratios, moves can be designed to ensure the conditions on the parameter are always met.

However, a tree is not a simple number or set of numbers, but a complex structure describing the arrangement of all the samples in a topology. To explore the tree space, we thus need to change not only the branch lengths, but also the order and the composition of all subclades of the tree. This requires a different set of MCMC moves, often called tree moves or topology moves. These moves propose rearrangements of the tree topology, and need to adjust or resample the associated branch lengths. Indeed, traversing tree space was a core challenge in developing phylogenetic applications of MCMC. Algorithms for efficiently sampling phylogenetic tree space became available in the late 1990s ( Mau & Newton, 1997; Mau *et al.*, 1999), when Bayesian approaches for phylogenetics began to appear. However, for more complex models, for instance models involving networks or multiple correlated trees, designing good tree moves remains an issue.

### 3.2 High-dimensionality models

Biology is complex, and therefore, models to describe the behavior of biological systems will also tend to be complex. Think for a moment about a phylogenetic substitution model, for example, the GTR + Γ model. In this model, each nucleotide (A,C,T,G) has a different frequency, and the rates of substitution between all pairs of nucleotides are different. In addition different sites of the alignment have different overall rates of substitution, modelled by a gamma distribution. Applied in a Bayesian context, the model has many parameters: a tree topology, the branch lengths on the tree, exchangeability rates between nucleotides, equilibrium state frequencies of the nucleotides, the parameters of the gamma distribution representing among-site rate heterogeneity. For even a small tree with few samples, this is many parameters. In addition, some of these parameters may be correlated, for instance the branch lengths of a timed tree and the average clock rate have an inverse relationship. As a result, many posterior spaces in phylogenetic inference are in configurations referred to as “rugged” ( Brown & Thomson, 2018), or having mixed areas of high probability (“peaks”) and areas of low probabilities (“valleys”). This ruggedness can make it difficult to use MCMC in high-dimensional space. As shown on Figure 2, MCMC will generally refuse to take a step if the proposed solution will be much worse than the current one. Thus the inference can end up trapped in local optima. New computational methods are required to traverse these types of rugged spaces. For example, using proposal algorithms which perturb several correlated parameters at the same time can make it easier to find alternative peaks in the posterior surface. For instance, the up-down operator in BEAST2 will scale both the branch lengths of the tree, and the clock rate simultaneously. In most cases, however, overcoming these difficulties require the developers to implement more efficient MCMC moves, which can be challenging.

In addition to traversal issues, more complex models can also suffer from performance issues in the likelihood calculation itself. A common problem for lineage diversification models such as birth-death processes, for instance, is that we do not observe the parts of the phylogeny which have not been sampled. Thus we are missing a large part of the true evolutionary process. When calculating the prior probability of the phylogeny given the diversification model, we have to account for all possible histories in the unobserved parts of the tree. In more complex models, this calculation will frequently involve numerical integration, which is computationally very expensive and can suffer from numerical instability, meaning that the probability value cannot be estimated for some parameter configurations. Although this issue can be improved by smart implementation of the models (see for instance the work done by Scire *et al.* (2022) on the BEAST2 package BDMM), it represents a fundamental limitations for more complex processes.

### 3.3 Inferring dated trees and incorporating fossils

Inferring dated trees is substantially more challenging than non-time constrained tree inference. First, phylogenetic characters do not contain information about absolute time, meaning rates and times are not fully identifiable. This means the posterior will be sensitive to the time priors, even when we have a large amount of sequence data ( Rannala & Yang, 2007; Yang & Rannala, 2006). Second, it requires the addition of a clock model and uses more complex tree models, usually coalescent or birth-death process models ( Drummond *et al.*, 2006; Thorne *et al.*, 1998; Yang & Rannala, 2006). It also requires additional time information. In macroevolutionary phylogenies, this time information generally comes from the fossil record, either in the form of node calibrations, or by directly including fossil specimens in the inference (sometimes called tip calibrations). Tip-calibrated analyses provide a better representation of the uncertainty associated with the fossil record, and arguably involve less subjective user choices, such as the choice of the distribution used for node calibrations ( Ronquist *et al.*, 2012). However, including fossils also presents specific challenges.

There are two main sources of uncertainty associated with fossils that should be considered in Bayesian inference: taxonomic or topological uncertainty and fossil age uncertainty. Inference under the fossilized birth-death (FBD) process can incorporate both phylogenetic and age information ( Heath *et al.*, 2014; Stadler, 2010). And because the model incorporates the fossil sampling process explicitly, extinct samples can be recovered as tips or *sampled* *ancestors* along internal branches. This requires special moves that propose changes to the total number of nodes in the tree, since each sampled ancestor reduces the number of tips by one ( Gavryushkina *et al.*, 2014; Heath *et al.*, 2014). In terms of data, we have two alternative options for informing the position of extinct samples within the tree. First, fossils with no character data can be assigned to a node using topological constraints. Constraints can be based on evidence from previous phylogenetic analyses or descriptive taxonomy. Using this strategy, the position of the fossil below the constraint node is sampled using MCMC. The precise position of the fossil cannot be inferred without character data, but the posterior output will reflect the uncertainty associated with fossil placement below the constraint node.

Alternatively, if morphological character data is available for fossil and extant samples, we can use a ‘total-evidence’ approach. Using this strategy, fossil placement can be sampled using MCMC and the position of taxa with character data can be inferred ( Barido-Sottani *et al.*, 2023; Gavryushkina *et al.*, 2017; Zhang *et al.*, 2016). This approach is conceptually preferable, since it more directly accounts for the phylogenetic uncertainty associated with fossils. In practice, however, character data is not available or limited for most groups (many discrete morphological matrices contain *<*100 characters) and, unlike DNA, character states can be subjective and uncertain ( Wright, 2019). Continuous character data, which might be more readily available for some groups, can also be used in total-evidence analyses ( Álvarez-Carretero *et al.*, 2019; Zhang *et al.*, 2024), although the use of this data type for phylogenetic inference needs to be carefully considered ( Varón-González *et al.*, 2020).

Fossil age uncertainty is straightforward to incorporate into Bayesian phylogenetic inference using the FBD process. Fossils are dated to within a known geological interval and the bounds of this age range (i.e., the minimum and maximum ages) can be used to inform priors on fossil ages. The age of fossils is then sampled during MCMC, therefore accounting for this uncertainty. This is preferable to fixing fossil ages to a point estimate within the known range of uncertainty, which can lead to erroneous parameter estimates ( Barido-Sottani *et al.*, 2019; Barido-Sottani *et al.*, 2020). In fact, fossil ages can be even be estimated using this approach ( Barido-Sottani *et al.*, 2022; Drummond & Stadler, 2016). Typically, a uniform distribution is used to model the age uncertainty associated with fossils, between the minimum and maximum possible ages based on stratigraphic and radiometric evidence. However, additional information could be used to construct more informative non-uniform priors on fossil ages.

## 4 Troubleshooting tools and techniques

### 4.1 How do I know if my MCMC is good?

Before we talk about troubleshooting, we first must figure out how we even know if there is anything to troubleshoot. We generally consider an MCMC inference to be complete when it reaches what is termed *convergence*. This is typically when a chain has arrived in its *stationary distribution*, that is, when additional sampling no longer affects the distribution of state values estimated. In plain language, once you are in the stationary distribution, you can do moves and change individual parameters, but the overall distribution of values will not change. The goal is to find this stationary distribution for all the parameters in your analysis. At the very least, users should ensure that the parameters primary interest to their research questions, along with the prior, likelihood and posterior, have converged satisfactorily. The phase before the chain has converged is called *burn-in*. The samples collected during burn-in should be discarded, usually 10–30% of the chain length, only keeping the remaining samples for the parameter estimation. For instance, by default MrBayes discards the beginning 25% samples as burn-in.

This sounds easy on the surface, but much ink has been spilled on appropriate ways of diagnosing whether or not our analysis has converged. Assessing convergence is usually done with convergence diagnostics. These are summary statistics that tell the researcher about how the MCMC inference, or chain, has performed and if it has converged. By far, the most commonly used diagnostic in phylogenetics is the *Effective Sample Size*, or ESS, which is calculated for every optimized parameter in the analysis separately (as well as for the likelihood, prior, and posterior).

When we perform MCMC inference, each time we do a move, we draw new values for one or more parameters, then accept or reject these values ( Figure 2). This is often called an MCMC step. Different software implementations and models will require different numbers of steps to reach convergence. You might think that the number of steps would be equivalent to the number of samples in the posterior sample. But in an MCMC chain, different steps will be correlated with one another. This is referred to as *autocorrelation*, and is the result of the fact that the parameter values present at step *i* are used to propose the parameter values for step *i* + 1 ( Figure 2). The ESS is specific to a posterior sample and to a given parameter, and describes the number of uncorrelated (independent) samples that would be needed to approximate the posterior distribution of a parameter with similar precision to that posterior sample. It is usually defined as ESS = *N/τ*, in which *N* is the number of generations and *τ* is the autocorrelation time. Due to autocorrelation, the ESS is typically smaller than the number of steps in the MCMC chain, because the difference between two successive samples is usually quite small. If we were drawing completely independent samples, the difference between sample *i* and sample *i* + 1 could be quite large (i.e., an independent sample could be drawn from anywhere in parameter space, so a series of such samples may explore the different areas of that space more quickly than when done step by step by an MCMC chain).

In practice, this means that we assess whether an MCMC chain has converged by checking whether the ESS for its parameters is above a certain number, meaning the chain has become stationary for long enough when we account for its autocorrelation. An ESS of over 200 has become the *de facto* standard in biological analyses, though reasons for this are largely arbitrary (but see section **Convenience**). Eventually, we would want the ESS for all parameters to be above the threshold, but there are some more insights to be gained along the way. The ESS of the likelihood and posterior are often treated as the indicator for overall convergence of the analysis, while the individual ESS of each optimized parameter is used to diagnose the specific causes of convergence issues. When inspecting the ESS along with the trace (e.g., in Tracer), they should tell a similar story: parameters with ESS above the threshold should show a stationary trace too, while for parameters with low ESS, the trace may not be stationary yet, and may in fact provide insights as to the nature of the problem. If the trace looks stationary but the ESS is still below the threshold, this may simply imply that it needs to run for a bit longer. Together, ESS and the trace should highlight which parameters struggle to converge, so that they can be targeted for adjustments. However, in practice it is not always that simple, since parameters are interdependent and affect each other’s convergence, so this needs to be kept in mind when searching for the culprit. It is also worth noting that certain types of parameters are by nature unlikely to ever converge. A prime example for this would be individual branch rates, if the tree is jointly inferred (e.g., in an FBD analysis) – since changes in tree topology change the position and numbering of branches, the trace output for these does not strictly follow the same branch after each step, thus there is no expectation of them converging along with the rest of the chain. In this case, the ESS of the overall variance and mean of branch rates is likely a better indicator of convergence.

Another simple way to check for convergence is to run several different chains for the same analysis. MCMC chains which use the same data, models and priors are guaranteed to converge on the same distribution, independent of the starting values used. Thus running multiple chains from different starting values and checking if the results obtained match is a good way to assess if the analysis has converged. In particular, certain issues can only be detected through the use of multiple chains, such as a chain getting stuck in a local optima due to the presence of several ‘peaks’ in the posterior (i.e., multimodality). As the ESS is based purely on samples present in the chain, it can reach good values even if the chain failed to reach the highest peak. Note that posterior samples from all chains can be combined together in the final result, thus the time spent on the different chains is not wasted.

In the next section, we will discuss software and tools for assessing ESS that were developed for Bayesian phylogenetics, as well as other avenues for understanding convergence issues. Other tools exist that were not developed with phylogenetics in mind, but are nonetheless also very useful, e.g., the R package coda ( Plummer *et al.*, 2006). Despite these tools, the assessment of MCMC convergence remains a challenging task in phylogenetic analyses, and future developments are needed to resolve some of these challenges. A promising example might be the use of L-lag couplings for Markov chains ( Biswas *et al.*, 2019) to estimate mixing and convergence jointly across the parameters of the tree ( Kelly *et al.*, 2023a; Kelly *et al.*, 2023b).

### 4.2 Tools of the trade

***4.2.1 Tracer.*** Tracer ( Rambaut *et al.*, 2018) is one of the most commonly used pieces of software for convergence assessment, due to the ease with which it can be used. A log file of sampled solutions from the MCMC can be read in. In its default view, a list of parameters in the model and their ESS value can be seen, as well as estimates of the value (mean, median, and spread) for each parameter sampled. Tracer automatically flags ESS values below a threshold of 200. Although this threshold value is somewhat arbitrary, it has been widely accepted in current practice as offering a good trade-off between convergence and computational cost of the inference.

**Figure 4.**
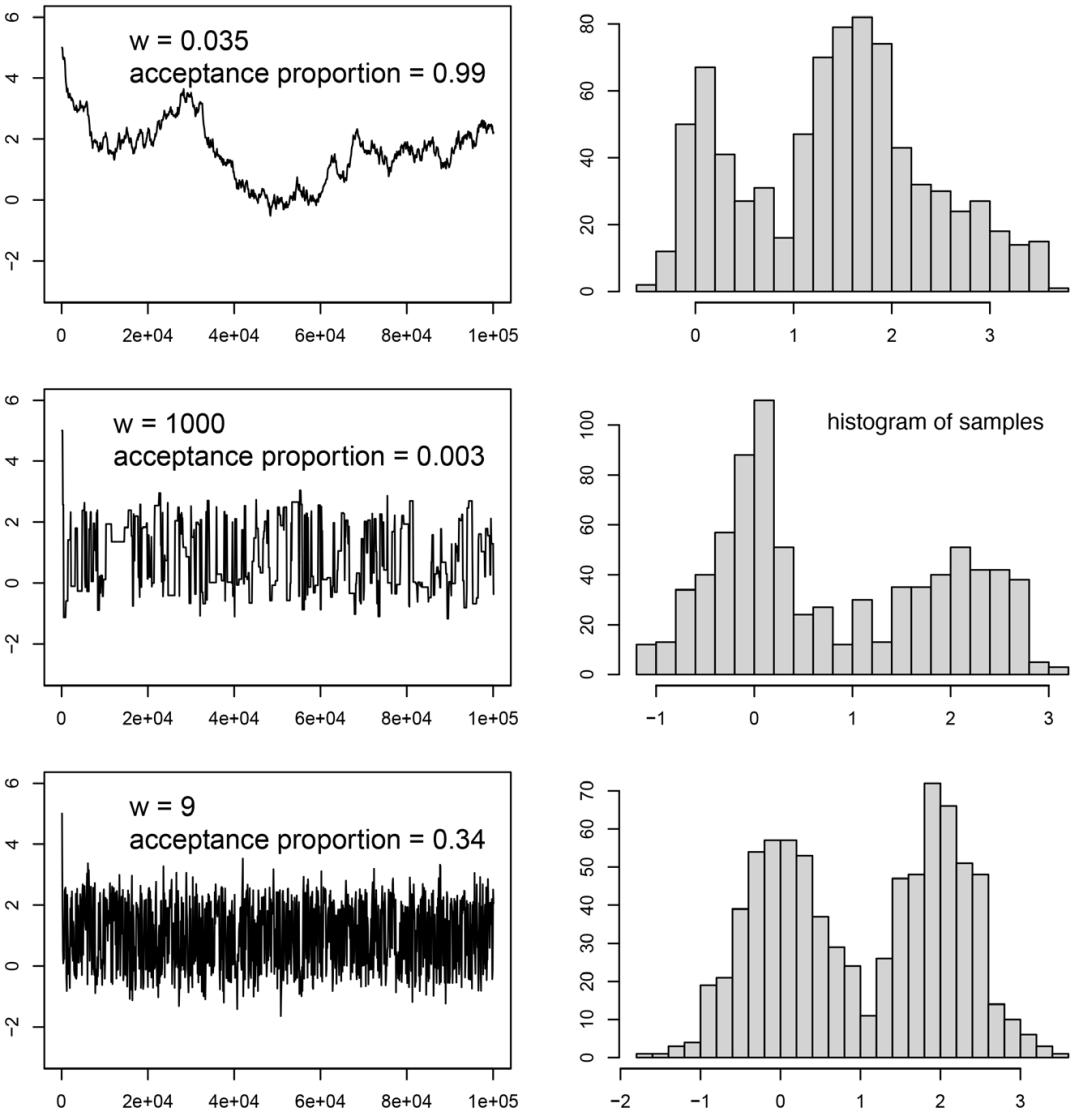
The target distribution is a half-half mixture of two normal distributions, one with mean 0 and standard deviation 0.5, the other with mean 2 and standard deviation 0.5. This distribution is estimated using MCMC with the sliding move (see Figure 3). The window size ( *w*) is a tuning parameter of the move. For each *w* value, the left panel shows the trace of the MCMC samples, while the right panel shows the histogram of the MCMC samples (discarding the first 20% samples as burn-in).

The trace panel, however, is most useful for debugging convergence issues (see the next section for some common issues). The trace window shows the values sampled for each parameter over the MCMC run. An example of different traces can be seen in Figure 4. Ideally, the trace will appear as what is often termed the “hairy caterpillar” ( Figure 4, last row). This is a sample that is well-converged. This pattern is generated by finding a good solution (or a set of good solutions) and sampling around that solution. Typically when this happens, the run has reached its stationary distribution.

***4.2.2 RWTY.*** Tracer remains the most used software for convergence, but it does not calculate an effective sample size for the most important model parameter − the tree itself. The ESS of the overall posterior or the ESS of parameters tied to the tree, such as the tree height or MRCA ages of specific clades, can be used as indirect signs of the (lack of) convergence of the phylogeny, however it is preferable to have a direct indicator. The R package RWTY (aRe We There Yet; Nylander *et al.*, 2008; Warren *et al.*, 2017) calculates an approximate ESS of the tree topology, which can provide additional information on the convergence of the tree. Additional graphical outputs can be generated in RWTY, such as treespace plots, which allow the visualization of how an MCMC chain explored parameters during its run.

***4.2.3 Convenience.*** Convenience ( Guimarães Fabreti & Höhna, 2022) is an R package that takes a fundamentally different approach to both how to calculate and how to assess ESS than RWTY and Tracer. It can produce visual outputs for convergence assessments, but also can produce simple text outputs stating if a run has converged or not.

ESS is still calculated in convenience. But rather than using an arbitrary threshold, such as an ESS of 200, convenience calculates a minimum threshold for a good ESS based on the standard error of the mean (SEM). The SEM allows a researcher to know how much error there is in the estimate of the posterior mean, compared to the variance of the posterior distribution. For this calculation, the posterior distribution is assumed to be shaped like a normal distribution, so the width of the 95% probability interval of the distribution is approximately equivalent to 4 *δ*, with *δ* being the standard deviation. This quantity is the reference used to calculate the threshold. By default, the ESS threshold in convenience is set to 625, which corresponds to an SEM equal to 1% of the interval width. By contrast, the threshold of 200 set by Tracer corresponds to an SEM of 1.77% of the interval width. Although higher ESS values are always better from a convergence point of view, they can also come at considerable computational cost, particularly for more complex analyses. Thus the choice of threshold should be adapted to each situation, for instance by using larger thresholds for critical parts of the inference and lower thresholds for less important estimates.

Convenience also allows the tree convergence to be estimated, by calculating the ESS of splits in the tree. A split represents a particular subclade of the tree, which can be either present or absent in each posterior sample. By calculating the ESS of all splits, we can thus obtain an estimate of the ESS of the tree topology. Finally, the reproducibility of an MCMC run is also considered by convenience. Two MCMC runs of the same analysis can be compared against each other using the Kolmogorov–Smirnov (KS) statistical test ( Kolmogorov, 1933; Smirnov, 1939), which tests if two samples were drawn from the same underlying distribution. If your two MCMC chains do not seem to be drawn from the same distribution, then this means your MCMC simulations are not consistently finding the same stationary distribution. This is likely due to one or both chains not having converged yet. It can also be indicative of the presence of multiple alternative possible solutions, with each chain finding a separate local optimum. Different slices of the same MCMC chain can also be compared against one another using the KS test to assess if the chain is in the process of converging.

## 5 Common issues and proposed resolutions

**Figure 5.**
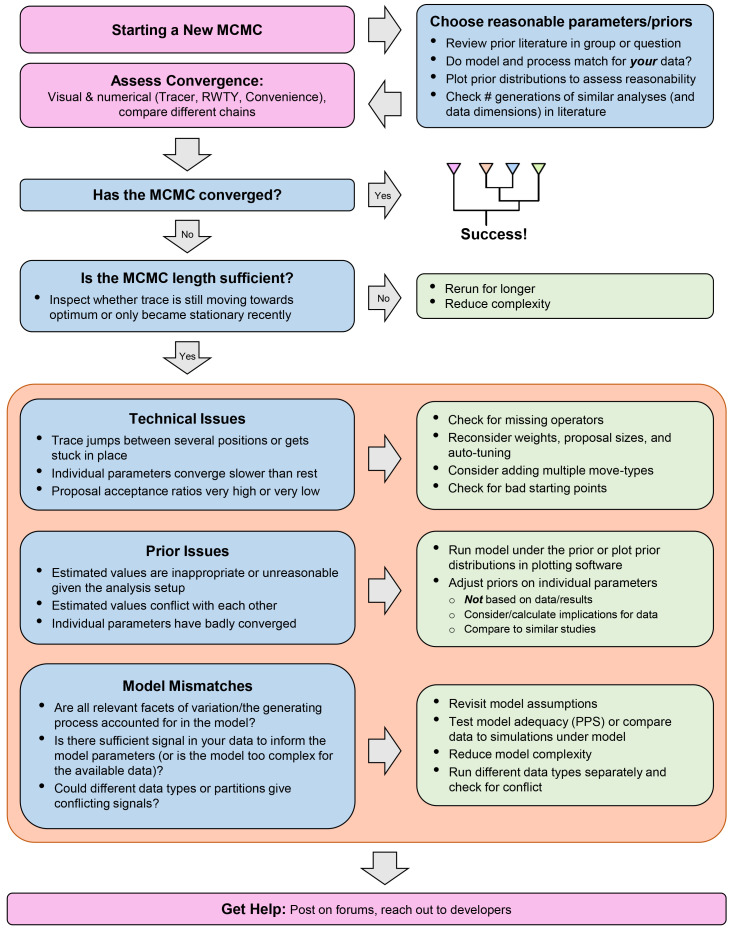
A flowchart to guide users through the MCMC-debugging process, highlighting key points mentioned in the text, with common issues in blue boxes and corresponding resolutions in green. Note that the different types of issues and resolutions within the orange box are not meant to be addressed in the order shown, but represent different avenues for investigating an issue.

As we have seen, MCMC analyses are composed of many different parts, which can make it difficult to identify the cause of problems. In this section, we detail some common issues which can affect the convergence of an MCMC inference, or even prevent it entirely from starting. An abbreviated overview of all the issues and resolutions described below can be found in Figure 5. Note that we focus in this article on issues and solutions from the perspective of a user of phylogenetic inference software. However, many issues (such as setups with strong correlations, as mentioned earlier) require further development work, and thus cannot be implemented by the users alone.

### 5.1 Inference technical setup

***5.1.1 Moves/operators.*** If an analysis does not converge well, or takes unreasonably long, it is worth checking the operators. Each parameter that is supposed to be estimated by the analysis needs to have at least one operator associated to it, in order to be optimised. If an operator is missing, that parameter will never change from its initial value, which not only means it will not converge, but also that other parameters can be prevented from converging properly. This issue tends to be more frequent in RevBayes, where there is no default operator setup proposed by the software and the choice of operators is left entirely to the user.

Another possibility is that the weights of the individual operators may need to be reconsidered (i.e., how often a new value should be proposed for the corresponding parameter). In some cases, some parameters are mixing well, and only a few specific ones are causing problems. In this case, it can help to increase the weight of the operators corresponding to badly-estimated parameters, so that more moves are being proposed each generation for them. Similarly, decreasing the weights of operators corresponding to well-estimated parameters will decrease the amount of computational time spent on proposals for these parameters, without affecting convergence too much.

Alternatively, if changing the weight did not fix the chain’s behaviour, we should consider its proposal size (i.e., how far from the current parameter value a proposed new value is). Many proposals, especially proposals on numerical parameters, include a configuration value which affects this size. A proposal size that is too small will make convergence of the corresponding parameter very slow, even at high operator weights ( Figure 4, top row), and may even trap the chain on a local optimum. If proposal sizes are too large instead, the chain may ‘overshoot’ the optimal parameter values or roam too far from them to converge properly. Overshooting optimal values may also mean that many proposals land on parameters with worse likelihood scores, which means they get rejected frequently and the chain experiences periods of stasis and thus overall poor mixing ( Figure 4, center row). The sampling pattern for that parameter may then also be too ‘coarse’ to properly capture the peaks and valleys in the likelihood.

A way to catch issues related to proposal-size is to check the final acceptance ratios for all operators, as well as the final trace. BEAST2 will even offer suggestions for adjusting the proposal sizes based on acceptance ratios at the end of the chain. Other than that, appropriate proposal sizes are not always straightforward for users to determine, but the problem can be alleviated in two ways: by letting the inference software auto-tune them, or by using a combination of proposals operating on the same parameter, but with varying proposal sizes. The latter is particularly helpful if the likelihood surface is very heterogeneous, as the chain then has a variety of step-sizes available, potentially increasing the likelihood that the appropriate one can be proposed. However, auto-tuning should be turned off if this strategy is chosen, so the separate proposal sizes will not change throughout the chain.

If auto-tuning is turned on (which is the default in BEAST2 and MrBayes, and optional in RevBayes), proposal sizes of moves will periodically be adjusted to guarantee a good acceptance ratio (e.g., to match a goal of 0.4). For example, if too many of the recently proposed moves were accepted, this might indicate that their size was too small (i.e., they may be slowly trudging uphill towards an optimum, see Figure 4, top row) and it will thus be increased. If too few proposals were accepted, this might in turn mean their size was too large (i.e., they shoot past optimal values into parts of parameter space with lower likelihoods, see Figure 4, center row) and will be decreased.

We generally recommend making use of such tuning features, but urge users not to mistake them for a magic solution to all proposal-size problems. Instead, one should be cautious not to ‘mis-tune’ or ‘over-tune’ the analysis. The main considerations when setting up auto-tuning are how often, and for how long to tune. Depending on the implementation, users can specify during what portion of the chain the parameters are tuned (i.e., during a dedicated tuning interval or burn-in phase, or throughout the run), and how often the parameters are being tuned during that interval. Tuning orients itself on the behaviour of the proposals during the chosen tuning intervals. Thus, these intervals need to be representative for the rest of the chain going forward, if the tuned values should be useful. In particular during the early stages of the MCMC (i.e., the burn-in phase), larger proposal sizes may be favoured as the chain moves from parameter values with low likelihood towards the optima, whereas smaller sizes might be favoured when exploring the likelihood surface around the optima. This generally means that proposals should possibly be re-tuned multiple times, to allow for feedback from the new behaviour of the tuned operator, and suggests that longer phases of tuning are needed for chains initialised at naïve starting values, than for those tailored to possibly start closer to the optima. However, if tuning intervals are kept too short, the available information might not be representative for the operator’s behaviour, resulting in unnecessary or inappropriate proposal size changes. Furthermore, while continuous tuning throughout the analysis can help account for the different requirements far from the optima versus close, there is a danger to tune towards the current location of the chain, homing in on smaller and smaller proposal sizes and thereby ‘trapping’ the chain on a local optimum. We would thus prefer to mainly tune during burn-in, and not during the main part of the analysis unless there is evidence that it is necessary. However, using the aforementioned strategy of multiple operators with varying, un-tuned proposal sizes might be a more helpful approach in such a case. Note that these changes can be integrated when running a new chain or when resuming the current one, as proposal configurations do not change the posterior distributions.

It can be difficult to identify which parameters exactly are causing the problem, since they can affect the mixing of others, blurring the picture. In particular, if the tree estimation has not converged, this can affect many other parameters. Often it is possible to identify the culprits by revisiting how the parameters are causally connected in the model. If available, a look at a schematic representation of the model might help getting more clarity on how different parameters may affect each other’s mixing. In BEAST2 or RevBayes, this representation can be obtained directly from the software (through BEAUti in the case of BEAST2, or by printing the model’s DAG [directed acyclic graph] in the case of RevBayes). MrBayes has the ‘showmodel’ command to show the current model, parameters in the model, and priors for the parameters. It also has the ‘showmove’ command to show the MCMC moves for the parameters.

***5.1.2 Starting values.*** Another problem is the initialisation of the MCMC chain at a ‘bad’ position. This means that our analysis started at a combination of parameter values that is either very far from the true values, or at a combination of values that is implausible or hard to compute given our data. As a result, the analysis may take much longer to converge (since it has to first slowly make its way out of the poorly fitting area of parameter space), or may crash altogether (e.g., because no likelihood could be calculated for conflicting parameter values). Ideally, users will have thought well about the possible values of all parameters and have set the respective prior distributions to favour the most plausible parameter values. However, the initial values are often left as the default (in BEAST2) or are picked at random from the prior (in RevBayes), so the chain can start in an unfavourable part of parameter space, or at an implausible combination of values. For example, we could start with some proposed very short branches along with a very low mutation rate, which could never explain the observed differences between the sequences of taxa. Or the starting values for the speciation and extinction rates could be implausibly high compared to the root age of the tree and its number of taxa.

To combat this, we usually have the option to specify the starting values for each parameter to something we deem reasonable. It may not always be straightforward to know what those values should be for a particular parameter, but beyond trial and error, a few standard options have been established. One possibility is to start at the expected mean of a prior distribution, which would be expected to work well as long as the prior distribution itself is sensible. Reminding oneself of the parameters’ biological meaning can also help to come up with a good solution. For example, speciation and extinction rates eventually just determine how many species we expect to arise and die out again over a given time period. Thus, a commonly used starting value for speciation rate is *λ* = *ln*( *nTips/*2) */rootAge*, which gives a simple estimate of net diversification (sometimes called the Kendall-Moran estimate; Baldwin and Sanderson, 1998), while extinction is set to *µ* = *λ/*10. Starting values can also be set for non-numerical parameters. Starting trees can be provided which may already be closer to the true solution (e.g., a quick maximum likelihood tree or a previously-published estimate) than a randomly drawn tree sample. However, attention has to be paid to the tree not being in conflict with other priors or constraints. For instance, the starting tree needs to be compatible with additional time information such as node calibrations, and with added constraints such as subclades (i.e., monophyletic sets of tips).

It is important to remember that starting values do not have to be spot-on estimates of where the actual true values lie, because after all, the MCMC is expected to go find those. The goal is merely to ensure that we have set a feasible combination of values for the chain to start from. Doing so does not only prevent computational issues (in case of unfeasible parameter combinations), but can also speed up the analysis (because we do not force the chain to trudge through parameter space that is far from the optima anyways, and instead allow it to start exploring feasible solutions instead). Also, it may prevent issues with the auto-tuning performed by the software. Since auto-tuning usually happens at the beginning of the inference, the behaviour of the moves may end up being tuned to suit a different part of parameter space than where the chain eventually should spend most of its time exploring, as described above. Of course, it is always advisable to run your analysis several times from different starting positions. This can help ensure that the analysis is not biased by its starting position. If running the analysis from different starting points leads to strongly different outcomes, this may indicate that the model is not adequately capturing the process that generated the data and is biased by its starting values.

***5.1.3 Inference algorithm*.** A final problem which can happen with MCMC is that the inference gets stuck in local optima for extended periods, which means not only that reaching convergence will take a very long time, but also that there is a risk of assuming the chain has converged when it has not. This issue is more frequent when the likelihood surface of the chosen model is more complex, and particularly for models where a lot of components are correlated (e.g., a multi-species coalescent model containing many different phylogenies which need to be consistent with each other). One sign that this is occurring is the presence of ‘plateaus’ in the trace, where the parameter value will oscillate in the same place for a lot of consecutive iterations, before jumping suddenly to a very different place in the parameter space. Another sign could be that independent runs generate inconsistent MCMC samples, indicating each run could potentially be sampling different local optima.

One potential way to resolve this issue is to use an expansion of the MCMC algorithm called Metropolis-coupled MCMC (MCMCMC or MC³, Altekar *et al.*, 2004). This algorithm relies on using multiple chains, weighted by a parameter called the ‘heat’. One chain (called the cold chain) has a heat of 1, meaning that it behaves like a regular MCMC chain; this is the chain that we will use to draw samples from the posterior. The other chains have a higher heat (of value < 1), which raises the posterior probability to a power, thereby flattening the likelihood surface ( Geyer, 1992; Gilks & Roberts, 1996). This means that heated chains can move more quickly and easily through the parameter space, and can reach points of high likelihood that the regular chain has not found yet ( Whidden & Matsen IV, 2015). However, as the heated chains do not evaluate the true posterior probabilities, we discard samples from such chains. Instead, the MCMCMC algorithm will use the heated chains to help explore the parameter space, and will periodically switch states between the cold chain and the heated chains (in an additional MCMC move), allowing the regular chain to ‘jump’ to new places in parameter space, as long as those new places have a higher posterior than the current ones.

MrBayes ( Altekar *et al.*, 2004), BEAST2 ( Müller & Bouckaert, 2020) and RevBayes currently support the MCMCMC algorithm, and it can be used along with any model or prior choice. MrBayes uses it by default (performing two independent runs and one cold chain and three heated chains per run). While MCMCMC potentially improves the performance of the inference, chain switching can mislead convergence assessment using regular ESS values ( Guimarães Fabreti & Höhna, 2022), and poorly chosen temperatures can lead to inefficient mixing and other issues, and should be looked out for ( Brown & Thomson, 2018).

### 5.2 Choice of model and priors

Even with all the technical aspects of the analysis set correctly, we can get convergence problems and faulty behaviour of the parameters. Such issues can either stem from unexpected interactions of priors, clashing components of the model, or mismatches of the model with the data. It can at first be challenging to distinguish those. If we do not already have a suspicion as to what the culprit might be (e.g., based on the trace, peculiarities of the data or model), one way to tell whether the issue lies with the analysis setup *per se* or with the pairing of data and model, is to run the MCMC ‘under the prior’. This means removing the likelihood from the posterior calculation, so that only values from the prior will be sampled and none of the data is involved. Thus, any remaining issues will be due to problems in the analysis setup, such as conflicting or interacting priors – and *vice versa*, if there are no such remaining issues, the problem may lie with the data or the model. Running the MCMC inference under the prior is useful not only for troubleshooting potential setup issues, but also for interpreting the results of the actual analysis. The difference between the prior distribution and the full posterior gives an estimate of how much of the signal present in the posterior sample actually comes from the sequence or character data, as opposed to the prior distributions. Note that although fossil ages are technically data, the probability of the tree under the FBD process given the fossil ages is considered part of the prior by BEAST2 and RevBayes. This can impact model selection and marginal likelihood estimators, as detailed in May and Rothfels (2023).

***5.2.1 Priors.*** The choice of good priors can make a big difference for the success of the MCMC. Of course, coming up with good priors is not trivial, and generally applicable advice is not always available. One difficulty is that priors should be clearly separated from the data. In a Bayesian inference, the probability of the data is accounted for by the likelihood. So, if the priors are also informed by the same data, then the information provided by the data ends up being counted twice by the inference, which will artificially increase its contribution to the posterior. Priors can thus be based on previous studies or biological knowledge, but *not* on analyses using the current dataset under study.

So how do we set priors? It may be tempting to just follow tutorials or use default priors at first, however, we strongly encourage users to think more critically about the implications of the prior choice for each individual analysis. While it is true that developers often design default settings to be a reasonable starting point for most analyses, they are by no means meant to be a one-fits-all solution, and one should not expect them to necessarily be an optimal or even good fit for ones own problem. As an extreme example, the default prior on some parameters in BEAST2 is a so-called improper prior, such as 1/X or Uniform(0, +∞), as the distribution does not integrate to one but to infinity. For example, the prior on the mean clock rate in BEAST2 is set to Uniform(0, +∞), because what constitutes a reasonable value for the clock rate is extremely dependent on both the organism and the timescale of the dataset. Thus it is up to the user to select a reasonable prior distribution for this parameter. Overall, improper priors are statistically problematic and should be avoided if possible. In general, your data or question may be quite different from what the method developers had anticipated, and often the behaviour of a model with different data and under different parameters is something that can only really be started to be explored once a new model/use case has been developed.

Thinking more carefully about the priors and their implications will go hand in hand with a deeper understanding of the model itself, which should be an additional encouragement to dive into it. The key is to remember that the prior distribution of a parameter represents the probability of those values being proposed during the MCMC, and values outside of it can never be tried. In particular, this means that long-tailed prior shapes, such as lognormal or exponential distributions, are often better than uniform distributions, which restrict the range of values which can be tried by the inference. Note also that priors always influence the results of the inference, and that setting very vague priors is not an optimal choice in most circumstances. For instance, in the example of the clock rate prior presented earlier, a prior distribution of Uniform(0, + *∞*) puts a lot of weight on very high values for that parameter, and will thus encourage the inference to try these values. If the data is not very informative on this particular parameter, this can result in estimated values which are absurdly high from a biological point of view, or can lead to convergence issues. A better prior would use our understanding of evolutionary processes to put more weight on biologically plausible values.

When choosing a prior, we thus need to consider what particular parameter values would imply for the data. For instance, substitution rates describe how fast mutations happen in the sequences and become fixed, and thus how much the sequences of the species under consideration could diverge over time.

Overall, in order to identify reasonable priors, we can ask the following questions:

- Have the parameters used in our analysis been estimated in other contexts or for similar datasets?
- What priors have similar studies chosen and how comparable is their data to ours? Note that these priors still need to be critically evaluated, as our understanding of plausible parameter values may have changed since the previous study.
- Does the range of parameter values allowed by the prior make sense given our data and analysis setup, for instance is it consistent with the expected number of substitutions in the alignment or the minimal clade ages?
- Can we do rough calculations to calibrate our prior expectations by, e.g., dividing the number of extant species by the assumed clade age, to get a rough estimate of net diversification?
- Can we obtain estimates for the parameters from sources outside of our dataset, for example using the fossil record to get an idea of how much extinction our focal clade may have experienced? Note that this requires making sure that the parameters chosen actually represent the same quantities between models, which is not always the case. For instance, extinction rates obtained from the fossil record represent a different parameter than death rates used in the fossilized birth-death process ( Silvestro *et al.*, 2018; Stadler *et al.*, 2018).

Although this may sound like a lot of work, it is also important to remember that identifying reasonable values for the different parameters, finding previous estimates for comparison, and evaluating the biological implications of the different values will always be needed to interpret the results of the analysis. The main difference in a Bayesian inference compared to other types of inference is that this work has to be performed upfront, rather than after the inference is finished.

It is generally advisable to plot the specified prior distributions and think about what they imply. Overall, the actual shape of the distribution (lognormal, gamma, etc.) is usually less important than the range of plausible values covered by the distribution (the 5% and 95% quantiles). However, the shape of the distribution affects the weight given to different parts of the range, i.e., whether low values are more likely under the prior than high values. Comparing the distributions for different values by using the visualization tool in BEAUti or plotting them in R is a great way to get a better idea of what is happening. It should be noted that simply looking at a curve may be misleading. Because the area under a certain section of the curve (e.g., a long tail) may still be large, even if the height of that section of the curve looks small. Thus, quantifying how much area is covered by the distribution (such as through quantiles) is still important. But in the case of node calibrations, even if each calibration is reasonable by itself, their combination can restrict the parameter space in unexpected ways ( Warnock *et al.*, 2015). This brings us back to running the analysis under the prior alone, as mentioned initially. This type of analysis can help spot situations in which the analysis is not specifying parameter distributions that the researcher considers reasonable. The effective prior on a node age in an inference will be the product of the prior set by the tree model, and of all additional calibration times set for the tree.

Finally, it can happen that information from previous studies leads to very different or even conflicting results for plausible parameter values in a specific dataset. One possible solution is to set the priors to be very uncertain and let the analysis decide which hypothesis is supported by the data, but this may not always be possible, and it risks creating convergence issues due to using vague priors. A good alternative is to run the same analysis using multiple different prior configurations, which will show how much the results and conclusions are impacted by the prior choices. If different priors yield clearly different results, further investigation is needed to determine what conclusions, if any, can be drawn from the inference. Possible next steps could include running the analysis under both sets of priors to test whether either setup is strongly biasing the result (and is thus in conflict with the data), or evaluating the biological information that informed the priors to begin with, and whether either of them is backed up more convincingly. If one (or several) parameters show prior sensitivity for both sets, this may indicate that there is not enough signal in the data to properly infer it, and one should explore the possibilities to add data or to simplify the model to avoid estimating this parameter, if feasible. If removing a parameter is not an option, model testing and adequacy testing could be employed to explore the most suitable prior configuration for the data, or model averaging could be used to integrate over the different priors to see what conclusions are still possible despite the lack of signal to inform this parameter. Even when there is no conflict in the literature, evaluating the robustness of the results when using different prior setups provides important information to inform which conclusions can be drawn from the analysis. This is especially important if running the analysis under the prior shows that the prior has a large influence on the results (i.e., the posterior distribution obtained with the data is very similar to the posterior distribution obtained with the prior alone).

***5.2.2 Model***. When the analysis is set up correctly and priors are reasonable, the cause for convergence problems may lie with the model itself, or how it relates to the data. It may seem daunting to choose between all the different types of models out there. There are a few pieces of software that can help researchers get an idea of plausible models. ClockstaR ( Duchêne *et al.*, 2014) can be used to choose appropriate relaxed clocks for molecular data. EvoPhylo ( Simões *et al.*, 2023) can do a similar selection for morphological data partitions. Although model selection can not be used to select between alternative birth-death sampling models because fossil ages are technically considered as part of the prior ( May & Rothfels, 2023), integrated tools in the Paleobiology Database website can also assist in finding reasonable starting parameters for FBD analyses. These tools use established paleontological methods for estimating parameters for speciation, extinction and fossilization rates. Using these sorts of tools can help with setting priors that have some support from the established literature.

If different data sources are being used for joint analyses, one might want to try running the different data separately in order to confirm whether they might support incompatible solutions. For example, in a total-evidence analysis, molecular and morphological data may each support different tree topologies. So when analysed jointly, solutions which could increase the likelihood of one type of data will decrease it for the other type, and vice versa, thereby making convergence around an optimal solution impossible. The same could apply to other combinations of data sources, e.g., conflicting molecular markers. Running the data for each type/partition separately can help a researcher determine if the convergence is poor due to methodological issues, or true signal conflict.

Much more fundamentally, the analysis might also just struggle to run or converge because the chosen model is not suitable for the data at all. Carefully revisiting the model’s assumptions and how those should manifest in the data is required to judge this, e.g., are there patterns of variation in our data, for instance between different groups, which the model needs to be able to address? An approach specifically designed to judge such model-data mismatches is model adequacy testing. This is done by simulating new data sets from the inferred posterior distributions, an approach termed posterior predictive simulations (PPS). These simulated data sets are then compared to the initial data using summary statistics which capture its relevant characteristics. If the model is inadequate to describe/analyse the variation in the data, that should be revealed through significant differences in the summary statistics between the data and the posterior simulations. These types of tests exist for a variety of phylogenetic models, including substitution models ( Bollback, 2002; Brown & ElDabaje, 2009; Lewis *et al.*, 2014; Nielsen, 2002), tree inference using molecular data ( Brown, 2014; Duchene *et al.*, 2019; Reid *et al.*, 2014) or morphological data ( Mulvey *et al.*, 2024), continuous and discrete trait evolution ( Blackmon & Demuth, 2014; Huelsenbeck *et al.*, 2003; Pennell *et al.*, 2015; Slater & Pennell, 2014), and diversification models ( Schwery & O’Meara, 2020; Schwery *et al.*, 2023). Approachable examples of such adequacy tests can be found on the RevBayes website ( https://revbayes.github.io/tutorials/intro_posterior_prediction/). However, that approach technically would require posterior estimates from a more or less successful MCMC, which would not be available if the analysis keeps crashing, and which would likely be uninformative if the MCMC did not converge. A good way to circumvent this would be to try and simulate datasets from scratch, based on more or less comparable parameters to the empirical data, and then compare them using the same summary statistics as one would use in the PPS approach. This would be akin to using approximate Bayesian computation (ABC, Sunnåker *et al.*, 2013). Exploring how the empirical data differs from what is expected under the model may allow you to judge the nature of the model-data mismatch.

Finally, it might be worth trying to reduce the complexity of one’s model. While it is tempting to make full use of the levels of complexity modern approaches allow us to model, one ought to consider whether there is enough information in the data for the model to work with. Just like any statistical test has sample size requirements to have the power to detect significant differences, these models need the data to have sufficient size and structure/heterogeneity to be able to infer parameter estimates without too much uncertainty. For example, we may want to use a relaxed clock model to account for the possibility that different parts of our tree evolve at different rates. But if we only have one fossil to calibrate our node ages with, or the sequences are not substantially variable, the model has limited information on which to base any rate differences on the tree. As a result, the different branch rates suggested by the MCMC will possibly meander around the parameter space without any receiving overwhelming support. Using a strict clock instead might neglect possible rate heterogeneity, but will at least be able to converge on reasonable estimates given the limited information available.

### 5.3 Data quality issues

In general, assembling more data leads to more precise and more accurate inferences. For example, previous research has shown that total-evidence studies require *∼*300 morphological characters to obtain reliable estimates of tree topology and divergence times in extinct clades ( Barido-Sottani *et al.*, 2020). Purely from a performance perspective however, it is important to note that additional data is not necessarily better for the convergence of an MCMC inference. Indeed, adding more data comes with added computational costs, and thus can have a net-negative impact on the performance, especially if the added data is very uncertain or conflicts with the rest of the data or with the chosen models and priors. For instance, Portik *et al.* (2023) built phylogenies using either a complete alignment of nuclear markers, a supersparse matrix of *∼*300 genes with large amounts of missing data, or the combination of both. They found that trees obtained using the combined dataset did not significantly differ from the trees obtained using the complete alignment alone. One possible avenue for resolving convergence issues is thus to remove genes or partitions which contain low amounts of information.

Similarly, increasing the number of extant or fossil samples in the tree leads to an exponential increase in the number of possible topologies, and so represents an important drag on performance. We typically select a subsample of extant taxa to be included in our analyses. We may assume extant taxa are sampled uniformly at random; but in many cases, they are sampled sparsely by keeping only one living representative per genus or subclade. The diversified sampling scheme has been implemented in the FBD model ( Zhang *et al.*, 2016) to accommodate such a case.

As mentioned above, there are two options for incorporating fossils directly in the phylogeny using the FBD process: assigning fossils to nodes via constraints or using morphological data in a total-evidence framework. Both approaches to positioning fossils present a challenge for MCMC inference, since even with character data, the topological uncertainty associated with fossils is typically large. And when there is a large amount of phylogenetic uncertainty, the posterior can span a broader flatter area, taking more effort to sample and making it harder to reach convergence. The use of very broad constraints (e.g., assigning all fossils to the root) in particular can lead to convergence issues, since there is insufficient information to inform the topology or other model parameters. To improve convergence, researchers could use the most precise constraints available, i.e., less inclusive nodes or lower taxonomic divisions, such as genera. In addition, it is possible to set a backbone extant tree, which will fix or strongly restrict the position of extant samples in the phylogeny, leaving only the positions of the fossils and the branch lengths to be estimated. That said, we emphasise that constraints should be implemented with extreme caution, as errors in constraints can lead to inaccurate results ( Barido-Sottani *et al.*, 2023). Having character data for fossils can help improve convergence, as it provides direct information about the topology. If convergence issues persist, provided additional taxonomic information is available, both approaches to fossil placement (the use of character data + constraints) could be combined. If additional taxonomic information or morphological data is unavailable, researchers might need to reconsider the scope of their analyses and the application of the FBD process to the data.

In addition to extant taxa, fossils are also usually sampled non-uniformly, with abundant fossils in some strata but rare in others. The FBD model can also take this into account by allowing the sampling rate of fossils to vary through time ( Gavryushkina *et al.*, 2014; Zhang *et al.*, 2016). To increase the biological realism of the FBD process, researchers might be tempted to incorporate variation in the sampling or diversification processes. This leads to an increase in the number of free parameters and another trade-off between model complexity and data availability that must be considered. Increasing the number of fossils will improve parameter estimation, leading to more accurate and precise estimates of the FBD model parameters, as well as divergence times and topology (provided the model is not strongly violated). However, users should bear in mind that adding fossils also increases overall tree size − each fossil is a tip or potential sampled ancestor, whose position must be sampled using MCMC.

This means that although fossils do not typically burden the computation through the addition of character data, which would increase the cost of calculating the likelihood, they increase the tree space, which will take longer to sample using MCMC. For many broader clades (e.g., mammals, animals, plants), including all fossil occurrences, while desirable, is not feasible. Presently, the maximum tree size that could reasonably be inferred using the FBD process is around 500 samples. One approach to get around this for large clades, or datasets with large numbers of fossil occurrences, is to randomly subsample the fossil data ( O’Reilly & Donoghue, 2020). This allows us to obtain a more manageable dataset without violating the sampling process assumptions.

Finally, if age uncertainty is substantial for many or all fossils in your analysis, the MCMC might also take longer to converge. However, compared to analyses that used fixed fossil ages, Barido-Sottani *et al.* (2019) showed using simulations, that incorporating fossil age uncertainty does not make the MCMC inference less efficient, i.e., more iterations are not always required to reach convergence, at least for data sets typical of Cenozoic mammals. This is probably because the use of fixed fossil ages introduces conflict into the tree space, leading to less efficient mixing.

### 5.4 How long should I run my analysis before giving up?

Some analyses take a long time to converge because it is hard to find the optimal values in a large parameter space, or because several local optima exist, and sometimes it takes a long time because a lot of uncertainty exists around the optimal values. Visually inspecting the parameter traces can give indications for this. Are they stabilising around certain values, still showing a trend into a certain direction, or just wildly meandering around? Trends in the trace can indicate that the MCMC is still searching for optimal values and just requires more time to find them (or perhaps needs to be restarted with starting values further in the suggested direction). But continuously rising or declining parameter values can also be pathological behaviour, suggesting misspecified priors or overly strict constraints in related parameters. Over-tuning of moves can also lead to such erratic behaviour, e.g., if the step-size for some parameters was tuned to be overly short or long. Wildly meandering traces could again be an indication of the data not containing enough information for those parameters to be identifiable, or step-sizes to be too long to allow it to settle around the optimal value.

As mentioned above, a behavior researchers sometimes observe is that an MCMC will appear to stabilize on a set of values, then jump to a completely different likelihood. This can be either an improvement (finding better values) or worse. This can happen because the analysis was previously stuck in a local optimum. That is, a region of parameter space that was good, but not the best in treespace. Thus, exploring this new optimum further is warranted. Or it may be that making a change to one parameter, such as the tree, causes a jump to a worse parameter space for other model parameters. In either event, this raises the question of how we can be sure that convergence has been achieved, and that the chain is not going to suddenly change further. Running multiple MCMC chains is an advisable way to discern between these two scenarios. Many software packages default to using two independent runs. Some, such as RevBayes and MrBayes, allow more to be used. Two to four runs are common in published analyses. Depending on the software used, each run either contains a single MCMC chain (such as BEAST2) or multiple chains (e.g., MrBayes uses MCMCMC by default which executes one cold chain and three heated chains per run).

Overall, the number of steps required to achieve convergence is difficult to estimate, as it will depend on all the components of the analysis, including the specific software used. Searching the literature for similar analyses, both in terms of dataset size and of models used, can provide a reasonable order of magnitude for the number of steps needed. The original publications of the specific model or package used, if available, will also provide estimates for what the original authors believed was a reasonable dataset size. Importantly, inference software all integrate a checkpointing mechanism, so analyses which have not converged can be resumed without losing the work already done. Thus it is not an issue if the initial number of steps is too low. Running several different chains with the same analysis can also be helpful in assessing how far the chain is from convergence. If the posterior distributions obtained by the different chains are largely mismatched, then convergence is likely still very far.

It is not uncommon for users of MCMC inferences to be aghast at the required run time. This is particularly the case when analyses are set up to incorporate too many different factors. Thus, minimizing the complexity of the setup from the start is generally a good idea. Ideally, we would want our analysis to be simple enough to be tractable, but complex enough to capture the relevant aspects of the data to answer our question. Unfortunately, the complexity that strikes that balance is often hard to determine *a priori* (or may not even exist for some combinations of question and data). While both gradually simplifying an overly complex model or gradually adding complexity to an overly simple model should be feasible strategies, we feel that erring on the side of simplicity may be more advisable. A successfully completed analysis that ends up being overly simplistic provides more information on how to improve it than an overly complex one that fails to run in the first place. Preliminary model testing, such as determining the most suitable substitution model using jModelTest ( Darriba *et al.*, 2012; Posada & Crandall, 1998; Posada, 2008), can help us narrow down an appropriate range of complexity to start at.

An important contributor to analysis complexity is the number of partitions, so it is good to consider whether they are all needed, and if some of them can share substitution or clock models. In particular, if you notice in the trace that parameters associated with some partitions are purely driven by the prior, then the data is likely over-partitioned. Similarly, using uncorrelated relaxed clock models increases the number of parameters by a large amount, as each branch of the tree is then associated with its own clock rate. If the dataset contains little time information, then there will be little signal in the data to estimate these rates, which is likely to lead to convergence issues. Luckily, there exist several tools to help determine what number of partitions may be best for a given dataset. For molecular data, the software package PartitionFinder ( Lanfear *et al.*, 2012; Lanfear *et al.*, 2017) can similarly be used to find partitions and test for the best substitution models for them. Its output files can be used as input for the aforementioned ClockStaR ( Duchêne *et al.*, 2014), to further determine which partitions require different clock models. We have already mentioned how EvoPhylo ( Simões *et al.*, 2023) can be used to partition morphological character data for clock models. There are a number of approaches to partitioning morphological data ( Casali *et al.*, 2023; Clarke & Middleton, 2008; Rosa *et al.*, 2019). However, conventional approaches used to select among partition schemes for substitution models cannot be used if the size of the Q matrix varies across partition schemes. An alternative approach in these circumstances is to use model adequacy ( Mulvey *et al.*, 2024). See also Casali *et al.* (2023) and Khakurel *et al.* (2024) for further details on the topic of partitions.

Beyond partitions, model adequacy testing (e.g., using posterior predictive simulations, PPS, as previously described in section 5.2.2) can also tell us whether our models are of appropriate complexity for the data. If the complexity of the model does not match that of the data, the differences in the summary statistics between the data and the posterior simulations should show that. However, as mentioned above, unlike preliminary model testing, PPS approaches come into play after the main analysis, as they rely on having the successfully inferred posterior distributions. Thus, starting with a level of complexity that prevents successful completion of an MCMC run will prevent us from using this approach.

Finally, note that using informative priors helps reduce the complexity of the analysis, by reducing the size of the parameter space that needs to be explored by the inference. This is especially true for parameters for which there is little signal in the data, such as the clock rate in an analysis with little time calibration information, or the extinction rate in an analysis with only extant species. For these parameters many different values will result in very similar posterior densities, so the inference can spend a large amount of time exploring a very wide plausible range of values. In this case constraining the values using fairly strict priors will ensure that the inference converges more quickly.

## 6 Good places to look for help

In addition to the guidance provided in this document, many software-specific resources can help in diagnosing and fixing misbehaving phylogenetic inferences. Bayesian inference frameworks are generally associated with a manual, some tutorials and help repositories which provide guidance on frequently used analyses. Specifically, users can look at the built-in help messages in MrBayes, the Taming the BEAST website ( https://taming-the-beast.org) for BEAST2 or the RevBayes website ( https://revbayes.github.io/tutorials/) for RevBayes. In particular, the RevBayes tutorial associated with this article ( https://revbayes.github.io/tutorials/mcmc_troubleshooting/) illustrates many of the issues introduced here, and presents troubleshooting advice targeted to the specific characteristics of the RevBayes framework.

For more detailed and targeted help, forums such as the BEAST user group ( https://groups.google.com/g/beast-users) or the RevBayes user forum ( https://groups.google.com/g/revbayes-users) are also available. Making good use of search engines can usually solve most common problems. If the problem appears to be due to a bug in the software (for instance, the inference crashes or returns non-sensical results), filing an issue on the Github repository is the best way to report it. Reporting an issue automatically alerts all developers, and makes the problem visible to other affected users. Note that for BEAST2, each package has a separate repository, so if the problem appears tied to a specific package the issue should be filed on the package repository rather than the general BEAST2 one. Before opening a new issue, you should make sure that the problem has not been reported already by looking through the list of open issues. As a last resort, developers can be contacted directly, although we recommend exploring the above resources first.

Several rules should be kept in mind when requesting help on forums or from tool developers and when filing issues. First, it is generally good to assume that any would-be helper will need to run the analysis themselves in order to identify the issue. Thus, all data, configuration and code files required to reproduce the problem should be included in the help request. The full error message or problematic output should also be included, so helpers can verify that they have correctly reproduced the issue. If possible, simplifying the analysis by removing elements which do not trigger the issue, or comparing the problematic analysis to a similar analysis which worked, will also be very helpful to track down a problem. Finally, detailed information on the computer configuration used (operating system type and version, software version, compiler version if the software was compiled manually, whether the analysis was run on a local machine or computer cluster) should be provided, particularly when the analysis crashes or fails to start.

## Ethics and consent

Ethical approval and consent were not required.

## Data Availability

No data are associated with this article.
