# Supplementary material for: Practical guidelines for Bayesian phylogenetic inference using Markov chain Monte Carlo (MCMC)
Source: Open Res Eur. 2026 Oct 13;3:204. Originally published 2023 Nov 20. [Version 4] doi: 10.12688/openreseurope.16679.4 (PMC10933576; doi:10.12688/openreseurope.16679.4)
Supplement: Peer review history

# Reviewer report: Reviewer response for version 3

**DOI:** 10.21956/openreseurope.19757.r46216

**Published:** 2024-12-24

**Authors:** David Černý

**Affiliations:** 1 The University of Chicago, Chicago, Illinois, USA

In this paper, the authors offer guidelines for setting up and troubleshooting phylogenetic Markov chain Monte Carlo (MCMC) analyses, with a particular emphasis on the inference of time trees using the fossilized birth-death (FBD) process. The manuscript first sets the scene by explaining the basic terminology (move / operator / proposal, convergence / stationarity, etc.), and as such it will serve as a gentle introduction to the topic for those researchers who are hoping to become more informed users of Bayesian phylogenetic methods.

 Overall, the manuscript retreads a lot of familiar ground (Fig. 4, showing traces produced by MCMC runs with moves that are too small or too large, feels like it has been recreated in the literature dozens of times now), but there is enough useful detail (for example, about differences between major software packages) to make it worth publishing. I have a lot of minor comments that are listed below, and some of these exemplify what I would consider to be two larger problems. First, the manuscript is not structured as well as it could be, which negatively affects the flow of the text. A lot of this has to do with jumping between different levels of abstraction. I would recommend explaining models first (highest level), then Bayesian inference of model parameters and how it differs from maximum likelihood, followed by concerns that are specific to phylogenetics (e.g., differences between dated and undated trees). Only then would I dive into an explanation of the phylogenetic MCMC, and give tips and recommendations about its use. As it stands, the authors start describing the MCMC before they even explain the meaning of the term “likelihood”, which just doesn’t work very well. There are several passages interspersed throughout the text that anticipate later sections of the manuscript, but look out of place when the reader first encounters them.

 My second concern has to do with several problematic instances where the authors seemingly misunderstood the very concepts they are trying to explain – these are likely due to pedagogical concerns about keeping things simple, or just sloppy use of terminology. For example, the authors suggest in several places that the MCMC optimizes parameter values or even searches for the true values (it does no such thing), they confuse the acceptance ratio with the Hastings ratio (while leaving the actual Hastings ratio unmentioned and unexplained), and the prior distribution with the proposal distribution. I have absolutely no doubt about the authors’ expertise, but such relatively basic errors are worrisome, and a manuscript that contains them cannot do the topic justice. Conditional on these errors being fixed, however, I can see the manuscript being useful to many biologists who want to learn more about the topic but are unsure where to start.

**Specific comments**

 Throughout the paper:

 (1) Inconsistent American vs. British spellings (behavior vs. behaviour, optimized vs. optimised).

 Abstract

 (2) I would take issue with the statement that “[m]odern statistical methods” employ models “involving fossil record information”. Sure, some do, but most active areas of phylogenetic research have nothing to do with analyzing fossil data. I understand the FBD model is of particular interest to the authors, but here they are perhaps getting a little ahead of themselves.

 (3) It seems backwards to mention the MCMC and posterior distributions before mentioning Bayesian analysis.

 Section 1

 (4) The first example of a model chosen by the authors is perhaps a bit unfortunate: the typical parameterization involves a transversion/transition *ratio*, rather than a separate parameter for transitions and another one for transversions.

 (5) The authors again seem to be getting ahead of themselves when they describe model parameters as random variables. That’s indeed how they are treated in Bayesian inference, but in maximum-likelihood estimation (and frequentist statistics more generally), every parameter is assumed to have a fixed but unknown value. Since the differences between these two frameworks have not yet been discussed at this point, the authors need to be more careful about not attaching interpretations to model parameters that are only valid under one of them.

 (6) “Bayesian inference thus offers a more complete picture of the results, integrating uncertainty in the results as well as existing information from previous studies” – Though I use and have developed Bayesian phylogenetic methods myself, I find this statement to be overselling things in an unacceptable way. First of all, most of the time, we as Bayesian phylogeneticists are desperate to find “flat” or “uninformative” priors (this includes seemingly mechanistic priors like the birth-death process, whose big attraction is actually the fact that it is flat over labeled histories), so the need to incorporate prior information is more of a burden than an advantage. Second, even when we do pick informative priors (and often it’s impossible not to), they are almost never based on previous studies. Third, the statement completely ignores the old but important objection that priors are subjective.

 (7) “true distribution of plausible parameters” – The authors mean “posterior”, not “true”. I’m not sure the notion of a “true distribution” even makes sense to me, since at the end of the day, I do believe there was just one true evolutionary history that generated my data, not a distribution of them (so I’m perhaps a likelihoodist at heart after all).

 Section 2.1

 (8) Once again, I have to point out that introducing the MCMC before Bayesian analysis seems to me like putting the cart before the horse. It’s really confusing that “likelihood” is only explained several paragraphs after bringing up “maximum likelihood”! I would strongly recommend rearranging this part of the MS.

 (9) 2.1.1 – Mentioning clock models and rates only makes sense in the context of inferring time trees; what about time-free / undated trees, where rates and times are confounded as branch lengths? This is yet another instance of concepts not being explained at a high enough level of abstraction when they are first introduced.

 (10) Also 2.1.1 – It may be worth pointing out that the single parameter of JC69 and Mk is not free, so there’s actually nothing for us to estimate in these models.

 (11) 2.1.2 – “a rate that is very high is believed to be less likely than one that is very short” – This sentence doesn’t work; rates can’t be “short”.

 (12) Also 2.1.2 – I expect many readers to not know what it means for parameters to not be identifiable.

 (13) 2.1.5 – The nomenclature I’m familiar with is that the acceptance ratio is the product of the posterior odds ratio and the Hastings ratio, so I find the authors’ attempt to conflate these two confusing. The actual Hastings ratio – i.e., p(theta | theta’) / p(theta’ | theta) – doesn’t make an appearance in the authors’ explanation, so what they are describing is actually the Metropolis algorithm rather than the more general Metropolis-Hastings algorithm.

 (14) 2.1.6 – The word “subclade” seems unnecessary here. I would just use “clade”.

 Section 2.2

 (15) Subsection 2.2.1 seems both misplaced and misnamed, since the authors focus on the difference between dated and undated (rather than rooted and unrooted) trees.

 (16) Also 2.2.1 – In addition to fossils, it may be good to mention the other common type of time-stamped data: serially sampled viral sequences.

 (17) 2.2.2 – This description is accurate but somewhat confusing, since it seems to conflate two kinds of modularity. One is a property of the models used, the other is a feature of software design.

 (18) 2.2.3 – While this is the RevBayes default and as such perhaps the only thing the reader needs to know about the program, RevBayes *can* be made to count iterations the MrBayes/BEAST way (Höhna et al. 2017).

 (19) Also 2.2.3 – It may be useful to point out that some special moves such as the slice move (Neal 2003) are supposed to have very high acceptance ratios.

 Section 3

 (20) “This can raise serious performance issues for our MCMC inference, either when exploring the tree space or when calculating the posterior probabilities” – Those are not two separate issues, but two ways of describing the same issue. If you are not doing a good job of exploring the tree space, you are not accurately approximating the posterior probabilities.

 (21) 3.1 – The second paragraph tries to reintroduce the topic of tree moves from scratch, despite the fact that they were already described (even including specific examples) several paragraphs above. Here, I would maybe recommend removing the earlier mention and merging it with this latter one to better preserve the flow of the exposition.

 (22) 3.2 – “the parameters of the gamma distribution representing among-site rate heterogeneity” – Usually, this distribution only has one free parameter, since its mean needs to equal 1.

 (23) 3.3 – The point about tip calibrations being less subjective than node calibrations is an interesting one that made me think. Is this actually the case, or are we just more comfortable placing uniform priors on tip ages compared to node ages? Node calibrations inherently involve more sources of uncertainty, but uncertainty and subjectivity are two different things, and many attempts have been made to design node calibrations in a more objective way (Wilkinson et al. 2011; Friedman et al. 2013; Matschiner et al. 2017; Claramunt 2022).

 (24) Also 3.3 – “additional information could be used to construct more informative non-uniform priors on fossil ages” – Yes, one could argue a non-uniform distribution would often be a better representation of our prior knowledge. What if a fossil comes from a Maastrichtian formation, but specifically from the base of the middle member of that formation? What if it is sandwiched between two radiometrically dated horizons, but is closer to one than the other? Etc. But that leads to two problems: (1) the subjectivity is back, big time; and (2) some implementations are not flexible enough to handle non-uniform tip age priors. MrBayes is, and BEAST 2 might be too, but while I don’t consider myself a RevBayes beginner, I’m not sure how I’d torture the program to allow them. Here, it might be worth distinguishing between what’s possible in principle and what’s possible in practice.

 Section 4

 (25) What I miss from the discussion of effective sample sizes is a clear statement that they only quantify the precision of the inference. They tell you whether you have enough samples to achieve a low standard error of the mean, but as the authors themselves point out, in and of themselves they say nothing about whether these samples come from the posterior distribution. With multiple chains, one would definitely hope that the only way to achieve high ESS values is for these chains to converge on the posterior, but even in that scenario there are certain caveats (e.g., it’s not impossible for multiple chains to get stuck in the same region of the parameter space if the posterior is very rugged and their starting values are similar or even the same, as can happen when the user supplies their own initial tree).

 (26) 4.1 – This is very pedantic, but the qualifier “typically” in the 3rd sentence of the 1st paragraph is unnecessary. In the context of MCMC, convergence is short for “convergence to the stationary distribution”; there is no other kind.

 (27) 4.1 (1st paragraph) – I would take the opposite view with regard to the relative dangers of setting the burnin too low/high. If you set it too low, you can always discard extra samples after the fact, but if you set it too high, you’ve wasted time and CPU cycles and there is nothing you can do.

 (28) 4.1 (3rd paragraph) – Is N the number of generations, or the number of moves? This might be a distinction worth drawing given the previous caveat about how different programs count generations in different ways.

 (29) 4.1 (4th paragraph) – “the individual ESS of each optimized parameter” – Wrong nomenclature. MCMC doesn’t optimize; it samples.

 (30) 4.1 (4th paragraph) – The point about branch rate ESS values being meaningless is an important one, but I would perhaps phrase it in a different way. It’s not that these are parameters that are unlikely to converge, it’s that our diagnostic tools conflate different parameters by giving them the same name (e.g., branch_rates[63] on one topology is a different parameter from branch_rates[63] on another topology). In principle, one could develop tools that do not do this. We could instead extract all unique branches present in the MCMC sample, and compute the ESS of each one’s rate.

 (31) 4.1 (5th paragraph) – “are guaranteed to converge” – I would qualify this statement with “when run for long enough”.

 (32) 4.1 (5th paragraph) – “in a local optima” – The word “optima” is plural. The corresponding singular is “optimum”.

 (33) Caption to Figure 4 – “is doing baby steps” – I’d prefer a more formal phrasing.

 (34) 4.2.2 – I would change the punctuation from “[…] of the phylogeny, however it is preferable […]” to “[…] of the phylogeny; however, it is preferable […]”.

 (35) Also 4.2.2 – I would also cite Lanfear et al. (2016) here, who first showed how to approximate topological ESS (with RWTY providing an implementation of their algorithms).

 (36) 4.2.3 – “This is likely due to one or both chains not having converged yet” – The qualifier “likely” is not needed. The stationary distribution of a correctly constructed MCMC sampler is the posterior distribution of the model, so if it is sampling from a distribution other than the posterior, it is by definition because it has not yet converged to its stationary distribution. The scenario where different chains get stuck at different local optima is not a different type of failure (as implied by the word “also”); rather, it is one possible reason why the chains have not converged to their stationary distribution.

 Section 5

 (37) 5.1.1 – “Each parameter that is supposed to be estimated by the analysis needs to have at least one operator associated to it, in order to be optimised” – Again, the MCMC doesn’t optimize. (It is closely related to an optimization technique called simulated annealing, but that’s beside the point here.) I’m surprised the authors would make such a basic mistake after explaining the difference between maximum likelihood and Bayesian inference.

 (38) Also 5.1.1 – “so that more moves are being proposed each generation for them” – Putting it this way presupposes the default RevBayes setup with multiple moves per generation; it doesn’t accurately describe what increasing the weight does in MrBayes or BEAST.

 (39) 5.1.2 (2nd paragraph) – I can think of absolutely no biological justification for setting the extinction rate at 1/10 of the speciation rate. In fact, paleobiology tells us that on average, speciation and extinction rates tend to be very nearly equal (Marshall 2017). This might not make for the best starting values, but in that case, let’s not pretend our starting values are motivated biologically rather than by computational convenience.

 (40) 5.1.2 (3rd paragraph) – “starting values do not have to be spot-on estimates of where the actual true values lie, because after all, the MCMC is expected to go find those” – No, the MCMC is not expected to find the true values. This is a fundamental misunderstanding of what it does, and one that I find quite disturbing in a paper that takes it upon itself to introduce the technique to others.

 (41) 5.1.2 (3rd paragraph) – “instead allow it to start exploring feasible solutions instead” – There is one more “instead” in this sentence than needed.

 (42) 5.1.3 – “each run could potentially be sampling different local optima” – Here, I’d use the singular: “each run could potentially be sampling a different local optimum”.

 (43) 5.2 – “the probability of the tree under the FBD process given the fossil ages is considered part of the prior by BEAST2 and RevBayes” – That’s correct for BEAST 2 but not for RevBayes. In the latter, if you run your analysis with the underPrior=TRUE option (available up to v1.2.4) or with the .ignoreAllData() option (which supplanted the former in v1.2.5), fossil ages will be discarded along with the character data. You never want this in an FBD analysis, since you’re not learning anything useful by pretending you don’t know if there were bears in the Jurassic or trilobites in the Miocene.

 (44) 5.2.1 (2nd paragraph) – “Overall, improper priors are statistically problematic and should be avoided if possible” – This is a bit of a controversial statement; authors from the objective Bayesian school (e.g., Jaynes 2003), including some phylogeneticists (Ronquist 2004), do not consider improper priors inherently problematic. There are, after all, improper priors that allow for proper posteriors, and have a clear meaning as the limit of a sequence of proper priors.

 (45) 5.2.1. (2nd pragraph) – I’m not sure I understand the authors’ point when they say the behavior of a model can only be explored once a new model has been developed. This could use some elaboration, or rephrasing.

 (46) 5.2.1 (3rd paragraph) – “The key is to remember that the prior distribution of a parameter represents the probability of those values being proposed during the MCMC” – No, the proposal distribution is generally not identical to the prior distribution. It can be (in which case the acceptance ratio reduces to the likelihood ratio), but that’s a rather crude way of setting up the MCMC that wouldn’t get us very far in phylogenetics. This is yet another worrying instance of the paper seemingly not having a firm grasp of the topic it tries to explain.

 (47) 5.2.1 (penultimate paragraph) – “The effective prior on a node age in an inference will be the product of the prior set by the tree model, and of all additional calibration times set for the tree” – This is true of BEAST and MrBayes, but not of other Bayesian phylogenetic software such as MCMCTree or RevBayes. Which is a good thing, because this “multiplicative construction” (Heled & Drummond 2012) violates basic rules of probability theory.

 (48) 5.2.1 (last paragraph) – “If different priors yield clearly different results, further investigation is needed to determine what conclusions, if any, can be drawn from the inference. Possible next steps could include running the analysis under both sets of priors to test whether either setup is strongly biasing the result” – So when we find that running the analysis under two sets of priors gives us different results, we should… run the analysis under two sets of priors? Again? Surely this doesn’t make sense.

 (49) 5.2.2 (1st paragraph) – “considered as part of the prior” – The word “as” is unnecessary.

 (50) 5.2.2 (1st paragraph) – The passage about using the PBDB to place priors on FBD parameters could definitely be expanded on, especially in light of the previously mentioned caveat that the origination and extinction rates inferred by traditional paleobiology don’t coincide with the birth and death rates of the FBD model.

 (51) 5.2.2 (2nd paragraph) – “thereby making convergence around an optimal solution impossible” – I think that’s the wrong way of putting it. The posterior would be bimodal in such a scenario, but that doesn’t in itself mean that the MCMC will have trouble converging on it. For an enlightening exploration of MCMC performance on tree mixtures, see Ronquist et al. (2006).

 (52) 5.2.2 (3rd paragraph) – “A good way to circumvent this would be to try and simulate datasets from scratch, based on more or less comparable parameters to the empirical data” – I have no idea what this means. Parameters can’t be “compared” to the data. If the alpha parameter of my gamma ASRV model is 0.234 and my sequences look like this: ACCGCGT…, ACTGGGT…, what sort of comparison am I supposed to make, and how did I get that alpha in the first place?

 (53) 5.3 – Surely the number of characters needed is a function of tree size? 300 characters will be overkill for 10 tips, but insufficient for 250 tips.

 (54) 5.3 (2nd paragraph) – Is FBD with diversified extant sampling available in any package other than MrBayes? If not, this might be worth mentioning.

 (55) 5.3 (3rd paragraph) – “assigning all fossils to the root” – To me, that would be an extremely narrow constraint, not a broad one! I guess the authors mean allowing fossils to attach anywhere in the tree (i.e., anywhere within the clade originating with the root node), rather than just along the root branch, but in that case, the description should be rephrased.

 (56) 5.3 (4th paragraph) – The piecewise-constant (“skyline”) fossil sampling rates described in the studies cited by the authors are actually not a great way of accommodating what we see in the fossil record, which often consists of a few Lagerstätten punctuated by long periods with almost no samples. The sampling burst model proposed by Magee & Höhna (2021) and implemented in RevBayes is much more promising, though underexplored and underutilized.

 (57) 5.4 – I would speak of searching for “high-probability” rather than “optimal” values, to make it clear that the MCMC is not an optimization algorithm.

 (58) 5.4 (4th paragraph) – The recommendation of jModelTest feels like a blast from the past given the fact that the substitution model is rarely the biggest contributor to overall model complexity (especially in the case of hierarchical models like FBD analyses with relaxed clocks), and in light of recent studies such as Abadi et al. (2019) and Fabreti & Höhna (2023) which show that the choice of the substitution model often doesn’t matter very much.

 Section 6

 (59) Please note that the RevBayes user forum has moved from https://groups.google.com/g/revbayes-users to https://github.com/revbayes/revbayes/discussions.

 References

 (60) The title of Kolmogorov’s 1933 paper is misspelled; it should end with “di distribuzione” (two separate words).

**References cited in this review but not in the paper**

Is the rationale for developing the new method (or application) clearly explained?

Yes

Is the description of the method technically sound?

Partly

Are the conclusions about the method and its performance adequately supported by the findings presented in the article?

Yes

If any results are presented, are all the source data underlying the results available to ensure full reproducibility?

No source data required

Are sufficient details provided to allow replication of the method development and its use by others?

Yes

Reviewer Expertise:

Phylogenetic method development; Bayesian phylogenetic inference; macroevolution; computational paleobiology

I confirm that I have read this submission and believe that I have an appropriate level of expertise to confirm that it is of an acceptable scientific standard, however I have significant reservations, as outlined above.

## References

[1] : Model selection may not be a mandatory step for phylogeny reconstruction. *Nat Commun* .2019;10(1) : 10.1038/s41467-019-08822-w934 30804347 10.1038/s41467-019-08822-w PMC6389923

[2] : CladeDate : Calibration information generator for divergence time estimation. *Methods in Ecology and Evolution* .2022;13(11) : 10.1111/2041-210X.139772331-2338 10.1111/2041-210X.13977

[3] : Nucleotide Substitution Model Selection Is Not Necessary for Bayesian Inference of Phylogeny With Well-Behaved Priors. *Syst Biol* .2023;72(6) : 10.1093/sysbio/syad0411418-1432 37455495 10.1093/sysbio/syad041

[4] : Molecular and fossil evidence place the origin of cichlid fishes long after Gondwanan rifting. *Proc Biol Sci* .2013;280(1770) : 10.1098/rspb.2013.173320131733 24048155 10.1098/rspb.2013.1733 PMC3779330

[5] : Calibrated tree priors for relaxed phylogenetics and divergence time estimation. *Syst Biol* .2012;61(1) : 10.1093/sysbio/syr087138-49 21856631 10.1093/sysbio/syr087 PMC3243734

[6] : Phylogenetic Inference Using RevBayes. *Curr Protoc Bioinformatics* .2017;57: 10.1002/cpbi.226.16.1-6.16.34 28463399 10.1002/cpbi.22

[7] : Estimating the Effective Sample Size of Tree Topologies from Bayesian Phylogenetic Analyses. *Genome Biol Evol* .2016;8(8) : 10.1093/gbe/evw1712319-32 27435794 10.1093/gbe/evw171 PMC5010905

[8] : Impact of K-Pg Mass Extinction Event on Crocodylomorpha Inferred from Phylogeny of Extinct and Extant Taxa. *bioRxiv* .2021; 10.1101/2021.01.14.426715 10.1101/2021.01.14.426715

[9] : Five palaeobiological laws needed to understand the evolution of the living biota. *Nat Ecol Evol* .2017;1(6) : 10.1038/s41559-017-0165165 28812640 10.1038/s41559-017-0165

[10] : Bayesian Phylogenetic Estimation of Clade Ages Supports Trans-Atlantic Dispersal of Cichlid Fishes. *Syst Biol* .2017;66(1) : 10.1093/sysbio/syw0763-22 28173588 10.1093/sysbio/syw076

[11] : A Broad Look at Tree-Building. *Science* .2004;303(5659) : 10.1126/science.1094283767-768 10.1126/science.1094283

[12] : Comment on "Phylogenetic MCMC algorithms are misleading on mixtures of trees". *Science* .2006;312(5772) : 10.1126/science.1123622367; author reply 367 16627724 10.1126/science.1123622

[13] : Dating primate divergences through an integrated analysis of palaeontological and molecular data. *Syst Biol* .2011;60(1) : 10.1093/sysbio/syq05416-31 21051775 10.1093/sysbio/syq054 PMC2997628

# Response

**Published:** 2025-12-09

**Authors:** Joëlle Barido-Sottani

**Affiliations:** Institute of Biology, Ecole Normale Superieure, Paris, Île-de-France, France

**Thank you very much for the review and the suggestions! Please find our detailed response to your comments below (in bold).**

 Comment: Overall, the manuscript retreads a lot of familiar ground (Fig. 4, showing traces produced by MCMC runs with moves that are too small or too large, feels like it has been recreated in the literature dozens of times now), but there is enough useful detail (for example, about differences between major software packages) to make it worth publishing. I have a lot of minor comments that are listed below, and some of these exemplify what I would consider to be two larger problems. First, the manuscript is not structured as well as it could be, which negatively affects the flow of the text. A lot of this has to do with jumping between different levels of abstraction. I would recommend explaining models first (highest level), then Bayesian inference of model parameters and how it differs from maximum likelihood, followed by concerns that are specific to phylogenetics (e.g., differences between dated and undated trees). Only then would I dive into an explanation of the phylogenetic MCMC, and give tips and recommendations about its use. As it stands, the authors start describing the MCMC before they even explain the meaning of the term “likelihood”, which just doesn’t work very well. There are several passages interspersed throughout the text that anticipate later sections of the manuscript, but look out of place when the reader first encounters them.

**Response: We are happy to hear that our work provides worthwhile information and appreciate the thoughtful suggestion for what might be an effective alternative structure for this paper, which would probably resonate well with some readers. However, after some consideration, we have decided against a major restructuring, for a couple of reasons. For one, while your suggested alternative structure makes sense conceptually and logically, we believe the current structure is also reasonable and approachable. While there are, as you noted, various instances where the text mentions topics that are only detailed further later on, that primarily happens during the first section, where it serves the purpose to give an overview of the general problem and the relevant concepts - which is something we expect at least some readers to find helpful. And while it is true that we end up jumping between the levels of abstraction, we think that reflects the route one takes when setting up or troubleshooting such models - a process which is usually not following a linear path up or down the levels of abstraction either. It is also not necessarily the intention of a paper like this to only be read from start to end, and so we expect readers to navigate back and forth between the sections relevant to their interest in any case. We did however add some sentences that make more clear to the reader that the first section provides more of an overview over the central issues, while the different elements of it are treated in more detail later on (in addition to the existing references to other relevant sections).**

 Comment: My second concern has to do with several problematic instances where the authors seemingly misunderstood the very concepts they are trying to explain – these are likely due to pedagogical concerns about keeping things simple, or just sloppy use of terminology. For example, the authors suggest in several places that the MCMC optimizes parameter values or even searches for the true values (it does no such thing), they confuse the acceptance ratio with the Hastings ratio (while leaving the actual Hastings ratio unmentioned and unexplained), and the prior distribution with the proposal distribution. I have absolutely no doubt about the authors’ expertise, but such relatively basic errors are worrisome, and a manuscript that contains them cannot do the topic justice. Conditional on these errors being fixed, however, I can see the manuscript being useful to many biologists who want to learn more about the topic but are unsure where to start.

**Response: We did indeed try and keep the language simple and approachable, and it seems this made us fall into the trap of employing commonly used, but technically incorrect phrasing (e.g., referring to parameters being optimised by the MCMC). Using these is probably hindering proper understanding of the topic in any case, but we agree that it is even more crucial to avoid this in an article like this. We replaced such instances with more correct wording and added a note on the misconception that the MCMC optimises parameters earlier in the text.**

 Comment: Throughout the paper:

 (1) Inconsistent American vs. British spellings (behavior vs. behaviour, optimized vs. optimised).

**Response:** **Fixed.**

 Abstract

  (2) I would take issue with the statement that “[m]odern statistical methods” employ models “involving fossil record information”. Sure, some do, but most active areas of phylogenetic research have nothing to do with analyzing fossil data. I understand the FBD model is of particular interest to the authors, but here they are perhaps getting a little ahead of themselves.

**Response: We have edited the abstract to clarify that this is just one example of phylogenetic applications.**

 (3) It seems backwards to mention the MCMC and posterior distributions before mentioning Bayesian analysis.

**​​​​​​​Response: We have restructured the abstract to introduce concepts in a more natural order.**

 Section 1

 (4) The first example of a model chosen by the authors is perhaps a bit unfortunate: the typical parameterization involves a transversion/transition *ratio*, rather than a separate parameter for transitions and another one for transversions.

**​​​​​​​Response: This was meant as a hypothetical, but yes, due to the resemblance to the actual K2P model, we’ve changed this.**

 (5) The authors again seem to be getting ahead of themselves when they describe model parameters as random variables. That’s indeed how they are treated in Bayesian inference, but in maximum-likelihood estimation (and frequentist statistics more generally), every parameter is assumed to have a fixed but unknown value. Since the differences between these two frameworks have not yet been discussed at this point, the authors need to be more careful about not attaching interpretations to model parameters that are only valid under one of them.

**​​​​​​​Response: We’ve edited this to make clear we’re discussing Bayesian models.**

 (6) “Bayesian inference thus offers a more complete picture of the results, integrating uncertainty in the results as well as existing information from previous studies” – Though I use and have developed Bayesian phylogenetic methods myself, I find this statement to be overselling things in an unacceptable way. First of all, most of the time, we as Bayesian phylogeneticists are desperate to find “flat” or “uninformative” priors (this includes seemingly mechanistic priors like the birth-death process, whose big attraction is actually the fact that it is flat over labeled histories), so the need to incorporate prior information is more of a burden than an advantage. Second, even when we do pick informative priors (and often it’s impossible not to), they are almost never based on previous studies. Third, the statement completely ignores the old but important objection that priors are subjective.

**​​​​​​​Response: We think this point represents a difference in schools of thought between ourselves and the reviewer. We no doubt agree that the use of priors as we’ve outlined is subjective: at no point do we argue for empirical Bayes or similar. However, we strongly feel that the ability to quantify our subjective prior intuition as a part of the model does indeed present a more complete picture of of the results. In no science is there true objectivity. Down to the process of hypothesis generation and data collection, we are codifying our biases into the research, but are doing so in such a way as to be unquantifiable. As such, uninformative priors, to the extent that they are possible (often it is not, see Zwickl and Holder 2004), still represent a statement that is in its core subjective.**

 (7) “true distribution of plausible parameters” – The authors mean “posterior”, not “true”. I’m not sure the notion of a “true distribution” even makes sense to me, since at the end of the day, I do believe there was just one true evolutionary history that generated my data, not a distribution of them (so I’m perhaps a likelihoodist at heart after all).

**​​​​​​​Response: You’re right. Changed.**

 Section 2.1

 (8) Once again, I have to point out that introducing the MCMC before Bayesian analysis seems to me like putting the cart before the horse. It’s really confusing that “likelihood” is only explained several paragraphs after bringing up “maximum likelihood”! I would strongly recommend rearranging this part of the MS. 

**​​​​​​​Response: While we can appreciate this point in general, we disagree with it in this particular instance. In a class-setting, one would present Bayesian analysis first and then introduce MCMC as an associated tool. But the MCMC and its workings are the main topic of this article, hence we start with it and then back up to cover basics of the Bayesian analyses it is used for. Furthermore, the purpose of this paper is primarily to advise on diagnosing and troubleshooting MCMC problems, rather than introducing the approach from scratch (we recapitulate them as a refresher and to ensure the readers and us are on the same page). So arguably, our intended audience, while novices, are not entirely new to those concepts. We are thus confident that the confusion caused by this should be minimal at best.**

 (9) 2.1.1 – Mentioning clock models and rates only makes sense in the context of inferring time trees; what about time-free / undated trees, where rates and times are confounded as branch lengths? This is yet another instance of concepts not being explained at a high enough level of abstraction when they are first introduced. 

**​​​​​​​Response: We now mention that clock models are only necessary when estimating a time tree. However, Bayesian inference is usually applied to time trees, so we feel like we do not lose in abstraction by introducing the clock model early.**

 (10) Also 2.1.1 – It may be worth pointing out that the single parameter of JC69 and Mk is not free, so there’s actually nothing for us to estimate in these models.

**​​​​​​​Response: We now mention that substitution models are normalized in a substitution-clock inference, and thus the JC69 and Mk parameters are fixed.**

 (11) 2.1.2 – “a rate that is very high is believed to be less likely than one that is very short” – This sentence doesn’t work; rates can’t be “short”. **Fixed.**

 (12) Also 2.1.2 – I expect many readers to not know what it means for parameters to not be identifiable.

**Good catch. Added a short explanation of this term.**

 (13) 2.1.5 – The nomenclature I’m familiar with is that the acceptance ratio is the product of the posterior odds ratio and the Hastings ratio, so I find the authors’ attempt to conflate these two confusing. The actual Hastings ratio – i.e., p(theta — theta’) / p(theta’ — theta) – doesn’t make an appearance in the authors’ explanation, so what they are describing is actually the Metropolis algorithm rather than the more general Metropolis-Hastings algorithm.

**Thank you for pointing this out! We have fixed this (see also our response to a similar comment from reviewer 5).**

 (14) 2.1.6 – The word “subclade” seems unnecessary here. I would just use “clade”. **Fixed.**

 Section 2.2

 (15) Subsection 2.2.1 seems both misplaced and misnamed, since the authors focus on the difference between dated and undated (rather than rooted and unrooted) trees.

**We agree that beyond the initial mention we don’t cover unrooted trees much and focus on dated vs not - we thus changed the subsection’s title to ’Types of trees’, which covers the whole content of the subsection. However, we disagree that the subsection is misplaced - while it admittedly isn’t strictly speaking covering the implementation of the MCMC, it is still crucial background to cover ahead of the following sections.**

 (16) Also 2.2.1 – In addition to fossils, it may be good to mention the other common type of time-stamped data: serially sampled viral sequences. **Good point, we added those.**

 (17) 2.2.2 – This description is accurate but somewhat confusing, since it seems to conflate two kinds of modularity. One is a property of the models used, the other is a feature of software design.

**We have clarified here that we are discussing the implementation of the frameworks, but that this implementation is made easier by the modularity of the models implemented.**

 (18) 2.2.3 – While this is the RevBayes default and as such perhaps the only thing the reader needs to know about the program, RevBayes *can* be made to count iterations the MrBayes/BEAST way (Höhna et al. 2017).

**Thanks for reminding us of this option. We have added a note about it, as some readers might find it useful.**

 (19) Also 2.2.3 – It may be useful to point out that some special moves such as the slice move (Neal 2003) are supposed to have very high acceptance ratios.

**Indeed, some moves are based on a Gibbs sampler which has an acceptance ratio of 1. This has been added in the paragraph.**

 Section 3

 (20) “This can raise serious performance issues for our MCMC inference, either when exploring the tree space or when calculating the posterior probabilities” – Those are not two separate issues, but two ways of describing the same issue. If you are not doing a good job of exploring the tree space, you are not accurately approximating the posterior probabilities. **Fixed.**

 (21) 3.1 – The second paragraph tries to reintroduce the topic of tree moves from scratch, despite the fact that they were already described (even including specific examples) several paragraphs above. Here, I would maybe recommend removing the earlier mention and merging it with this latter one to better preserve the flow of the exposition.

**We added a little more bridge between these two sections. In the first, we explain how these moved work. But the old moves aren’t sufficient for new tree types, such as networks, for example. This remains an ongoing problem.**

 (22) 3.2 – “the parameters of the gamma distribution representing among-site rate heterogeneity” – Usually, this distribution only has one free parameter, since its mean needs to equal 1. **Changed to singular.**

 (23) 3.3 – The point about tip calibrations being less subjective than node calibrations is an interesting one that made me think. Is this actually the case, or are we just more comfortable placing uniform priors on tip ages compared to node ages? Node calibrations inherently involve more sources of uncertainty, but uncertainty and subjectivity are two different things, and many attempts have been made to design node calibrations in a more objective way (Wilkinson et al. 2011; Friedman et al. 2013; Matschiner et al. 2017; Claramunt 2022).

**Good point. We have clarified that the uncertainty we are referring to here is mainly about the placement of fossil taxa in the tree and added a note with the recommended citations.**

 (24) Also 3.3 – “additional information could be used to construct more informative non-uniform priors on fossil ages” – Yes, one could argue a non-uniform distribution would often be a better representation of our prior knowledge. What if a fossil comes from a Maastrichtian formation, but specifically from the base of the middle member of that formation? What if it is sandwiched between two radiometrically dated horizons, but is closer to one than the other? Etc. But that leads to two problems: (1) the subjectivity is back, big time; and (2) some implementations are not flexible enough to handle non-uniform tip age priors. MrBayes is, and BEAST 2 might be too, but while I don’t consider myself a RevBayes beginner, I’m not sure how I’d torture the program to allow them. Here, it might be worth distinguishing between what’s possible in principle and what’s possible in practice.

**We have added a note with these caveats.**

 Section 4

 ​​​​​​​(25) What I miss from the discussion of effective sample sizes is a clear statement that they only quantify the precision of the inference. They tell you whether you have enough samples to achieve a low standard error of the mean, but as the authors themselves point out, in and of themselves they say nothing about whether these samples come from the posterior distribution. With multiple chains, one would definitely hope that the only way to achieve high ESS values is for these chains to converge on the posterior, but even in that scenario there are certain caveats (e.g., it’s not impossible for multiple chains to get stuck in the same region of the parameter space if the posterior is very rugged and their starting values are similar or even the same, as can happen when the user supplies their own initial tree).

**Pointing this out more explicitly would indeed be an improvement. We have done that in the paragraph where we already pointed out that issue and reorganised it accordingly.**

 (26) 4.1 – This is very pedantic, but the qualifier “typically” in the 3rd sentence of the 1st paragraph is unnecessary. In the context of MCMC, convergence is short for “convergence to the stationary distribution”; there is no other kind. **Fixed.**

 (27) 4.1 (1st paragraph) – I would take the opposite view with regard to the relative dangers of setting the burnin too low/high. If you set it too low, you can always discard extra samples after the fact, but if you set it too high, you’ve wasted time and CPU cycles and there is nothing you can do.

**Agreed. It depends on whether we are only concerned with the effect on the posterior vs. the time-efficiency of our analyses too (and whether the burnin is auto-discarded or not). We have updated the paragraph to reflect these points too.**

 (28) 4.1 (3rd paragraph) – Is N the number of generations, or the number of moves? This might be a distinction worth drawing given the previous caveat about how different programs count generations in different ways.

**We have clarified that the ESS is calculated based on the number of posterior samples recorded and so does not depend on the implementation chosen for the generations.**

 (29) 4.1 (4th paragraph) – “the individual ESS of each optimized parameter” – Wrong nomenclature. MCMC doesn’t optimize; it samples. **Changed to “estimated” parameter.**

 (30) 4.1 (4th paragraph) – The point about branch rate ESS values being meaningless is an important one, but I would perhaps phrase it in a different way. It’s not that these are parameters that are unlikely to converge, it’s that our diagnostic tools conflate different parameters by giving them the same name (e.g., branch rates[63] on one topology is a different parameter from branch rates[63] on another topology). In principle, one could develop tools that do not do this. We could instead extract all unique branches present in the MCMC sample, and compute the ESS of each one’s rate.

**Thank you for this comment. We have clarified that this is not an issue with the parameters themselves, but rather how we represent them in the trace.**

 (31) 4.1 (5th paragraph) – “are guaranteed to converge” – I would qualify this statement with “when run for long enough”. **Fixed.**

 (32) 4.1 (5th paragraph) – “in a local optima” – The word “optima” is plural. The corresponding singular is “optimum”. **Fixed.**

 (33) Caption to Figure 4 – “is doing baby steps” – I’d prefer a more formal phrasing.

**This has been changed accordingly.**

 (34) 4.2.2 – I would change the punctuation from “[. . . ] of the phylogeny, however it is preferable [. . . ]” to “[. . . ] of the phylogeny; however, it is preferable [. . . ]”. **Fixed.**

 (35) Also 4.2.2 – I would also cite Lanfear et al. (2016) here, who first showed how to approximate topological ESS (with RWTY providing an implementation of their algorithms). **Added.**  

 (36) 4.2.3 – “This is likely due to one or both chains not having converged yet” – The qualifier “likely” is not needed. The stationary distribution of a correctly constructed MCMC sampler is the posterior distribution of the model, so if it is sampling from a distribution other than the posterior, it is by definition because it has not yet converged to its stationary distribution. The scenario where different chains get stuck at different local optima is not a different type of failure (as implied by the word “also”); rather, it is one possible reason why the chains have not converged to their stationary distribution.

**We have edited this section as suggested.**

 Section 5

 (37) 5.1.1 – “Each parameter that is supposed to be estimated by the analysis needs to have at least one operator associated to it, in order to be optimised” – Again, the MCMC doesn’t optimize. (It is closely related to an optimization technique called simulated annealing, but that’s beside the point here.) I’m surprised the authors would make such a basic mistake after explaining the difference between maximum likelihood and Bayesian inference.

**Good point, edited to sampled.**

 (38) Also 5.1.1 – “so that more moves are being proposed each generation for them” – Putting it this way presupposes the default RevBayes setup with multiple moves per generation; it doesn’t accurately describe what increasing the weight does in MrBayes or BEAST.

**Changed wording to apply for either case.**

 (39) 5.1.2 (2nd paragraph) – I can think of absolutely no biological justification for setting the extinction rate at 1/10 of the speciation rate. In fact, paleobiology tells us that on average, speciation and extinction rates tend to be very nearly equal (Marshall 2017). This might not make for the best starting values, but in that case, let’s not pretend our starting values are motivated biologically rather than by computational convenience.

**Agreed, the ’biological meaning’ argument was referring to λ, while μ = λ/10 is merely reporting what is common practice. We have clarified that accordingly now.**

 (40) 5.1.2 (3rd paragraph) – “starting values do not have to be spot-on estimates of where the actual true values lie, because after all, the MCMC is expected to go find those” – No, the MCMC is not expected to find the true values. This is a fundamental misunderstanding of what it does, and one that I find quite disturbing in a paper that takes it upon itself to introduce the technique to others. **Fixed.**

 (41) 5.1.2 (3rd paragraph) – “instead allow it to start exploring feasible solutions instead” – There is one more “instead” in this sentence than needed. **Fixed.**

 (42) 5.1.3 – “each run could potentially be sampling different local optima” – Here, I’d use the singular: “each run could potentially be sampling a different local optimum”. **Fixed.**

 (43) 5.2 – “the probability of the tree under the FBD process given the fossil ages is considered part of the prior by BEAST2 and RevBayes” – That’s correct for BEAST 2 but not for RevBayes. In the latter, if you run your analysis with the underPrior=TRUE option (available up to v1.2.4) or with the .ignoreAllData() option (which supplanted the former in v1.2.5), fossil ages will be discarded along with the character data. You never want this in an FBD analysis, since you’re not learning anything useful by pretending you don’t know if there were bears in the Jurassic or trilobites in the Miocene.

**We have removed the specific mention of the software as this is implementation-specific, and as the reviewer notes, can change with different versions. Instead we now explain this issue in general terms.**

 (44) 5.2.1 (2nd paragraph) – “Overall, improper priors are statistically problematic and should be avoided if possible” – This is a bit of a controversial statement; authors from the objective Bayesian school (e.g., Jaynes 2003), including some phylogeneticists (Ronquist 2004), do not consider improper priors inherently problematic. There are, after all, improper priors that allow for proper posteriors, and have a clear meaning as the limit of a sequence of proper priors.

**We have now added more information on improper priors and their potential issues. As we mentioned earlier, we take position here for subjective priors, which should be as informative as possible, whereas the goal of improper priors in objective Bayesian analysis is to be as uninformative as possible.**

 (45) 5.2.1. (2nd paragraph) – I’m not sure I understand the authors’ point when they say the behavior of a model can only be explored once a new model has been developed. This could use some elaboration, or rephrasing.

**The point of this section was that models are frequently used for applications or in scenarios beyond what the original developers anticipated, which is another reason relying on default setups can be an issue. We have edited this part to make our reasoning clearer.**

 (46) 5.2.1 (3rd paragraph) – “The key is to remember that the prior distribution of a parameter represents the probability of those values being proposed during the MCMC” – No, the proposal distribution is generally not identical to the prior distribution. It can be (in which case the acceptance ratio reduces to the likelihood ratio), but that’s a rather crude way of setting up the MCMC that wouldn’t get us very far in phylogenetics. This is yet another worrying instance of the paper seemingly not having a firm grasp of the topic it tries to explain.

**You are right, we apologize for this mistake and have removed it from the article. We now provide more information on the proposal distribution in the Move section.**

 (47) 5.2.1 (penultimate paragraph) – “The effective prior on a node age in an inference will be the product of the prior set by the tree model, and of all additional calibration times set for the tree” – This is true of BEAST and MrBayes, but not of other Bayesian phylogenetic software such as MCMCTree or RevBayes. Which is a good thing, because this “multiplicative construction” (Heled & Drummond 2012) violates basic rules of probability theory. The main point we wanted to make in this paragraph is that users can and should visually inspect the effect priors, which can diverge from the user specified priors even without the multiplicative construction, i.e., see Warnock et al. 2015, PRSB.

**We have slightly modified the sentence to avoid implying that all software uses the multiplicative approach and added further citations to this paragraph for any readers interested in exploring the topic further. We wished to avoid mentioning specific software implementations here, as the options available across different software are quite complicated and subject to change. ** (48) 5.2.1 (last paragraph) – “If different priors yield clearly different results, further investigation is needed to determine what conclusions, if any, can be drawn from the inference. Possible next steps could include running the analysis under both sets of priors to test whether either setup is strongly biasing the result” – So when we find that running the analysis under two sets of priors gives us different results, we should. . . run the analysis under two sets of priors? Again? Surely this doesn’t make sense. 

**In this case, we’re specifically addressing the circumstance under which you’ve run the analysis with a few different configurations of priors, and are getting conflicting results. In that case, it’s worth looking at what the priors tell you without the data involved. We have edited to make it clearer that we’re referring to running under the prior here.**

 (49) 5.2.2 (1st paragraph) – “considered as part of the prior” – The word “as” is unnecessary. **Fixed.**

 (50) 5.2.2 (1st paragraph) – The passage about using the PBDB to place priors on FBD parameters could definitely be expanded on, especially in light of the previously mentioned caveat that the origination and extinction rates inferred by traditional paleobiology don’t coincide with the birth and death rates of the FBD model. 

**We have added a note about differences in rates parameters between fossils versus trees but feel that a much longer discussion about this is beyond the scope of this paper.**

 (51) 5.2.2 (2nd paragraph) – “thereby making convergence around an optimal solution impossible” – I think that’s the wrong way of putting it. The posterior would be bimodal in such a scenario, but that doesn’t in itself mean that the MCMC will have trouble converging on it. For an enlightening exploration of MCMC performance on tree mixtures, see Ronquist et al. (2006).

**While the MCMC can in theory recover bimodal and multimodal posterior distributions, in our experience this has often not been the case, unless mixture models are explicitly accounted for in the analysis. We have edited this section to make it clear that convergence is not impossible, but is likely to encounter issues. ** (52) 5.2.2 (3rd paragraph) – “A good way to circumvent this would be to try and simulate datasets from scratch, based on more or less comparable parameters to the empirical data” – I have no idea what this means. Parameters can’t be “compared” to the data. If the alpha parameter of my gamma ASRV model is 0.234 and my sequences look like this: ACCGCGT.. . , ACTGGGT.. . , what sort of comparison am I supposed to make, and how did I get that alpha in the first place?

**We agree that the original wording may be a bit confusing to some, so we edited that paragraph for clarity, leading with the mention of ABC approaches instead.**

 (53) 5.3 – Surely the number of characters needed is a function of tree size? 300 characters will be overkill for 10 tips, but insufficient for 250 tips.

**We have added that the referenced study used trees of 200 tips and now mention that this number is likely to vary based on tree size and dataset uncertainty.**

 (54) 5.3 (2nd paragraph) – Is FBD with diversified extant sampling available in any package other than MrBayes? If not, this might be worth mentioning.

**The availability of diversified sampling has been added to the end of the paragraph.**

 (55) 5.3 (3rd paragraph) – “assigning all fossils to the root” – To me, that would be an extremely narrow constraint, not a broad one! I guess the authors mean allowing fossils to attach anywhere in the tree (i.e., anywhere within the clade originating with the root node), rather than just along the root branch, but in that case, the description should be rephrased. **Fixed.**

 (56) 5.3 (4th paragraph) – The piecewise-constant (“skyline”) fossil sampling rates described in the studies cited by the authors are actually not a great way of accommodating what we see in the fossil record, which often consists of a few Lagerst¨atten punctuated by long periods with almost no samples. The sampling burst model proposed by Magee & Höhna (2021) and implemented in RevBayes is much more promising, though underexplored and underutilized. 

**This would depend on the dataset - the importance of Lagerstätten varies. It might be important for groups such as insects or some vertebrates, but less important for some well preserved marine invertebrates like bivalves or brachiopods. Therefore, we do not wish to say that the skyline model is not useful. However, we have added the recommended citation and added a description of this model.**

 (57) 5.4 – I would speak of searching for “high-probability” rather than “optimal” values, to make it clear that the MCMC is not an optimization algorithm. **Changed.**

 (58) 5.4 (4th paragraph) – The recommendation of jModelTest feels like a blast from the past given the fact that the substitution model is rarely the biggest contributor to overall model complexity (especially in the case of hierarchical models like FBD analyses with relaxed clocks), and in light of recent studies such as Abadi et al. (2019) and Fabreti & Höhna (2023) which show that the choice of the substitution model often doesn’t matter very much.

**Thank you for bringing up those studies. Indeed, testing for substitution models was merely meant to be one straightforward example for the idea of running preliminary tests on model components (as it used to be common practice), rather than being an explicit recommendation that this should be the most important test to run (as will surely become clear to readers when we highlight partitioning and adequacy testing in the following paragraphs). But we agree that the relative (un)importance of substitution models in phylogenetic inference should be clarified, so we now do so and cite those two papers. However, we still chose to keep the mention of jModelTest, so the paper can act as a guide to tools and resources which are commonly used in the community, even if some inferences may not benefit from its use.**

 Section 6

 (59) Please note that the RevBayes user forum has moved from https://groups.google.com/g/revbayes-users to https://github.com/revbayes/revbayes/discussions. **Fixed.**

 References

 (60) The title of Kolmogorov’s 1933 paper is misspelled; it should end with “di distribuzione” (two separate words). **Fixed.**

# Reviewer report: Reviewer response for version 3

**DOI:** 10.21956/openreseurope.19757.r46198

**Published:** 2024-12-17

**Authors:** Peter Beerli

**Affiliations:** 1 Florida State University, Tallahassee, Florida, USA

Review of Practical guidelines for Bayesian phylogenetic Inference using Markov chain Monte Carlo (MCMC) [version 3]

 I enjoyed reading the 'Practical Guidelines'. It offers a good overview of the field of Bayesian inference in phylogenetics and gives good advice on potential problems with complex analyses, highlighting possible ways to find good solutions. I have a few suggestions for improving clarity in some sections. I have added several citations that may better trace the history of the method or give credit to researchers who did similar/same analyses earlier. My biggest concern was Figure 1 and the section describing the MCMC decision ratio, the Metropolis-Hastings ratio. I hope the citations and guidance will help improve this section. All my comments are suggestions, and you should decide whether to incorporate them or not.

 Detailed comments with page and line references:

 Abstract:

 3rd line: 'solutions' seems vague

 line 5: 'evaluated' I would prefer 'estimated' or 'found'

 line 7 from bottom: I would prefer direct wording: We offer an overview...

 Page 4: Introduction: for the 34 million trees you cite Degnan and Salter 2005,

 which is a fine citation, but there were earlier citations, for example, Felsenstein 1978 (there may be even earlier ones, but I do not know them; the groundwork for counting trees was given by A. Cayley in 1889, but he does not elaborate on specific numbers.

 Felsenstein, J. (1978) [Ref-1].

 Cayley, A. (1889) [Ref-2]

 the next sentence: 'It is intractable to evaluate all of them.' This statement is incorrect, evaluating 34 million items is certainly in the realm of current computers (for example, Swoffords's PAUP* can evaluate and enumerate all 10-tip (perhaps up to 12 tips) trees in a parsimony context. It is certainly tedious. How about: 'Evaluating all phylogenies with a number of samples larger than 10 become quickly intractable.'

 "That technique is Markov chain Monte Carlo (MCMC)," the next sentences show potential alternatives with citations, it seems odd that you do not cite MCMC here.

 Some reviewers suggested to show that alternatives exist. I find showing these "alternatives" that are 'variants' of MCMC in the introduction unnecessary but

 could be discussed later towards the end of the tutorial. I suggest to cite Metropolis et al. here. Concerning the citation of the alternative methods given that this is early in the introduction, readers may want to have a citation of the method and not the application in phylogenies, for example, SMC: Doucet et al. 2001 [or perhaps Gordon 1998 associated with introduction of SMC), HMC: Duane et al. [I would remove the other methods and move them somewhere else but still cite Metropolis et al.]

 Arnaud Doucet, Nando de Freitas, and Neil Gordon. (2001) [Ref 12]

 ****Figure 1 and text discussing figure 1: MCMC was introduced by Metropolis et al. and the outline in Figure 2 follows the 'Metropolis algorithm' and should be

 labeled the 'Metropolis ratio' but see below: Hastings (1970) generalized the Metropolis algorithm because the original ratio assumes that the proposal distributions for the newly proposed parameter and the old parameters are symmetric, i.e., g(x|y) = g(y|x). In phylogenetics, we do not necessarily guarantee this condition. Modern implementations use the Metropolis-Hastings algorithm to allow for potential asymmetries in the proposal. Figure 1 is labeled 'Hastings ratio,' and a reader needs to know why this is called 'Hastings ratio' because Hastings is not mentioned in the text. I suggest revising the text to include Hastings (1970) and relabeling the 'Hastings ratio' to 'Metropolis-Hastings ratio.'

 I also suggest adding context after the Metropolis et al. citation on page 5, along these thoughts: "The original algorithm assumed that the moves in the parameter space are symmetric, going from parameter value a to parameter value b has the same probability as  going from b to a. Hastings (1970) introduced an additional term to

 balance potentially asymmetric proposals; this led to a general version: the Metropolis-Hastings algorithm. I would remove the sentence, "In this case, all the values ... phylogenetics. And then, in figure 1, use 'Metropolis-Hastings ratio': with minimal change to the figure to resolve the issue, one could simply add in the figure legend: "We left out the Hastings part of the ratio for clarity; it would add g(old|new)/g(new|old) to R."

 Citation: W. K. Hastings, 1970. [Ref-4])

 page 6 last 3 lines of 2.1.2 use high vs low (not high vs short)

 page 6 first column, bottom and second column top: I suggest to cite the authors who defined the methods: path sampling in phylogenetics (Lartillot and Phillipe 2006), Stepping Stone (Xie et al. 2010), nested sampling (Russel et al. 2018 [applies Skilling 2006 to phylogenetics] and very recently, a Partition-Weighted Kernel method (Wang et al., 2023).

 Nicolas Lartillot and Herve, 2006 [Ref-5]

 Wangang Xie, 2011. [Ref-6].

 Yu-Bo Wang, et al., 2023. [Ref-7].

 page 6 second column: For instance, reversible jump can be used to infer the most adequate substitution model for an alignment (Huelsenbeck et al. 2004) [they estimate

 the best substitution model among 200+ models 13 years before Bouchaert and Drummond]

 John P. Huelsenbeck, et al., 2004. [Ref-8].

 page 9, second column: Algorithms for efficiently sampling phylogenetic trees … (citations, see below) this would be a good location to show the early Bayesian phylogenetic methods because they all had to solve the tree-changing problem and this will give a good way to give credit to early work on Bayesian phylogenetics, for example, Li often gets forgotten.

 Shuying Li. Phylogenetic tree construction using Markov chain Monte Carlo. Journal of Computational Biology, 3(3):463–474, 1996. doi: 10.1089/cmb.1996.3.463.[Ref - 13]

 Bob Mau and M. A. Newton., 1997.[Ref-9].

 Bruce Rannala and Ziheng Yang, 1996. [Ref 10].

 Bret Larget and Donald L. Simon, 1999. [Ref- 11].

 page 11 "(e.g., in tracer)" You have a section later on Tracer; I would simply remove this () because, at this point, the reader does not know that you mean the tracer application.

 page 16 left bottom: The sentence may confuse the reader by using 'temperature' from 1 to 0. Usually, we would consider the Metropolis-Hastings ratio powered with 1/T, where we would set the cold chain at temperature T=1 and also consider hotter chains with higher T, for example, a T=1,000,000 would set that exponent to approximately zero so that all moves will be accepted; any intermediate value will accept more often than the cold chain.

 page 16 right 5.2. choice of models and priors "This means removing the likelihood..." A user will not be able to do that, so perhaps suggest that replacing the DNA nucleotide data with all '?' has the same effect as removing the likelihood or setting the log-likelihood to zero.

 page 17 top left: this paragraph is correct but also reflects the position of a subjective Bayesian, there are empirical Bayes approaches that estimate priors from the data.

Is the rationale for developing the new method (or application) clearly explained?

Yes

Is the description of the method technically sound?

Yes

Are the conclusions about the method and its performance adequately supported by the findings presented in the article?

Yes

If any results are presented, are all the source data underlying the results available to ensure full reproducibility?

No source data required

Are sufficient details provided to allow replication of the method development and its use by others?

Yes

Reviewer Expertise:

I develop Bayesian inference methods in population genetics [population genetics, MCMC, Bayesian inference]

I confirm that I have read this submission and believe that I have an appropriate level of expertise to confirm that it is of an acceptable scientific standard, however I have significant reservations, as outlined above.

## References

[1] : The Number of Evolutionary Trees. *Systematic Zoology* .1978;27(1) : 10.2307/2412810 10.2307/2412810

[2] : A Theorem on Trees. *Paris: The Quarterly Journal of Mathematics, 23, 376-378* .1889;

[3] : Hybrid Monte Carlo. *Physics Letters B* .1987;195(2) : 10.1016/0370-2693(87)91197-X216-222 10.1016/0370-2693(87)91197-X

[4] : Monte Carlo Sampling Methods Using Markov Chains and Their Applications. *Biometrika* .1970;57(1) : 10.2307/2334940 10.2307/2334940

[5] : Computing Bayes factors using thermodynamic integration. *Syst Biol* .2006;55(2) : 10.1080/10635150500433722195-207 16522570 10.1080/10635150500433722

[6] : Improving marginal likelihood estimation for Bayesian phylogenetic model selection. *Syst Biol* .2011;60(2) : 10.1093/sysbio/syq085150-60 21187451 10.1093/sysbio/syq085 PMC3038348

[7] : LoRaD: Marginal likelihood estimation with haste (but no waste). *Syst Biol* .2023;72(3) : 10.1093/sysbio/syad007639-648 36856704 10.1093/sysbio/syad007

[8] : Bayesian phylogenetic model selection using reversible jump Markov chain Monte Carlo. *Mol Biol Evol* .2004;21(6) : 10.1093/molbev/msh1231123-33 15034130 10.1093/molbev/msh123

[9] : Phylogenetic Inference for Binary Data on Dendograms Using Markov Chain Monte Carlo. *Journal of Computational and Graphical Statistics* .1997;6(1) : 10.1080/10618600.1997.10474731122-131 10.1080/10618600.1997.10474731

[10] : Probability distribution of molecular evolutionary trees: a new method of phylogenetic inference. *J Mol Evol* .1996;43(3) : 10.1007/BF02338839304-11 8703097 10.1007/BF02338839

[11] : Markov Chasin Monte Carlo Algorithms for the Bayesian Analysis of Phylogenetic Trees. *Molecular Biology and Evolution* .1999;16(6) : 10.1093/oxfordjournals.molbev.a026160750-759 10.1093/oxfordjournals.molbev.a026160

[12] Sequential Monte Carlo Methods in Practice.2001; 10.1007/978-1-4757-3437-9 10.1007/978-1-4757-3437-9

[13] : Phylogenetic tree construction using Markov chain Monte Carlo /, Ph.D. thesis,The Ohio State University, The Ohio State University.1996;

# Response

**Published:** 2025-12-09

**Authors:** Joëlle Barido-Sottani

**Affiliations:** Institute of Biology, Ecole Normale Superieure, Paris, Île-de-France, France

**Thank you very much for the review and the suggestions! Please find our detailed response to your comments below (in bold).**

 Abstract:

 3rd line: ’solutions’ seems vague **Changed as the whole sentence was restructured.**

 line 5: ’evaluated’ I would prefer ’estimated’ or ’found’ **Agreed, changed to ’estimated’.**

 line 7 from bottom: I would prefer direct wording: We offer an overview... **Changed to the more direct ”We thus offer an overview...”**

 Page 4: Introduction: for the 34 million trees you cite Degnan and Salter 2005, which is a fine citation, but there were earlier citations, for example, Felsenstein 1978 (there may be even earlier ones, but I do not know them; the groundwork for counting trees was given by A. Cayley in 1889, but he does not elaborate on specific numbers. **Added.**

 the next sentence: ’It is intractable to evaluate all of them.’ This statement is incorrect, evaluating 34 million items is certainly in the realm of current computers (for example, Swoffords’s PAUP* can evaluate and enumerate all 10-tip (perhaps up to 12 tips) trees in a parsimony context. It is certainly tedious. How about: ’Evaluating all phylogenies with a number of samples larger than 10 become quickly intractable.’

**We changed this sentence following the suggestion.**

 ”That technique is Markov chain Monte Carlo (MCMC),” the next sentences show potential alternatives with citations, it seems odd that you do not cite MCMC here. Some reviewers suggested to show that alternatives exist. I find showing these ”alternatives” that are ’variants’ of MCMC in the introduction unnecessary but could be discussed later towards the end of the tutorial. I suggest to cite Metropolis et al. here. Concerning the citation of the alternative methods given that this is early in the introduction, readers may want to have a citation of the method and not the application in phylogenies, for example, SMC: Doucet et al. 2001 [or perhaps Gordon 1998 associated with introduction of SMC), HMC: Duane et al. [I would remove the other methods and move them somewhere else but still cite Metropolis et al.]

**We’ve added this citation and moved the discussion of other algorithms to figure 2.**

 ****Figure 1 and text discussing figure 1: MCMC was introduced by Metropolis et al. and the outline in Figure 2 follows the ’Metropolis algorithm’ and should be labeled the ’Metropolis ratio’ but see below: Hastings (1970) generalized the Metropolis algorithm because the original ratio assumes that the proposal distributions for the newly proposed parameter and the old parameters are symmetric, i.e., g(x—y) = g(y—x). In phylogenetics, we do not necessarily guarantee this condition. Modern implementations use the Metropolis-Hastings algorithm to allow for potential asymmetries in the proposal. Figure 1 is labeled ’Hastings ratio,’ and a reader needs to know why this is called ’Hastings ratio’ because Hastings is not mentioned in the text. I suggest revising the text to include Hastings (1970) and relabeling the ’Hastings ratio’ to ’Metropolis-Hastings ratio.’

**We have added more context on the ratio as suggested here and in the following legend. We also now discuss the**

**proposal distribution in more details in the move section.**

 I also suggest adding context after the Metropolis et al. citation on page 5, along these thoughts: ”The original algorithm assumed that the moves in the parameter space are symmetric, going from parameter value a to parameter value b has the same probability as going from b to a. Hastings (1970) introduced an additional term to balance potentially asymmetric proposals; this led to a general version: the Metropolis-Hastings algorithm. I would remove the sentence, ”In this case, all the values ... phylogenetics.

**We have edited this part according to your suggestion.**

 And then, in figure 1, use ’Metropolis-Hastings ratio’: with minimal change to the figure to resolve the issue, one could simply add in the figure legend: ”We left out the Hastings part of the ratio for clarity; it would add g(old—new)/g(new—old) to R.” 

**Thank you for pointing this out and for providing such a clean solution. We have implemented these suggestions.**

   page 6 last 3 lines of 2.1.2 use high vs low (not high vs short) **Fixed.**

 page 6 first column, bottom and second column top: I suggest to cite the authors who defined the methods: path sampling in phylogenetics (Lartillot and Phillipe 2006), Stepping Stone (Xie et al. 2010), nested sampling (Russel et al. 2018 [applies Skilling 2006 to phylogenetics] and very recently, a Partition-Weighted Kernel method (Wang et al., 2023).

**Thank you for bringing this to our attention. We have added more details to this section and now mention the PWK method.**

 page 6 second column: For instance, reversible jump can be used to infer the most adequate substitution model for an alignment (Huelsenbeck et al. 2004) [they estimate the best substitution model among 200+ models 13 years before Bouchaert and Drummond] **Fixed.**

 page 9, second column: Algorithms for efficiently sampling phylogenetic trees . . . (citations, see below) this would be a good location to show the early Bayesian phylogenetic methods because they all had to solve the tree-changing problem and this will give a good way to give credit to early work on Bayesian phylogenetics, for example, Li often gets forgotten.

**Thank you for the suggestion, we have added these references.**

 page 11 ”(e.g., in tracer)” You have a section later on Tracer; I would simply remove this () because, at this point, the reader does not know that you mean the tracer application. **Fixed.**

 page 16 left bottom: The sentence may confuse the reader by using ’temperature’ from 1 to 0. Usually, we would consider the Metropolis-Hastings ratio powered with 1/T, where we would set the cold chain at temperature T=1 and also consider hotter chains with higher T, for example, a T=1,000,000 would set that exponent to approximately zero so that all moves will be accepted; any intermediate value will accept more often than the cold chain. 

**This has been refined as suggested.**

 page 16 right 5.2. choice of models and priors ”This means removing the likelihood...” A user will not be able to do that, so perhaps suggest that replacing the DNA nucleotide data with all ’ ?’ has the same effect as removing the likelihood or setting the log-likelihood to zero.

**This has been refined as suggested.**

 page 17 top left: this paragraph is correct but also reflects the position of a subjective Bayesian, there are empirical Bayes approaches that estimate priors from the data.

**This is a good point, added in the introduction to the prior section: The above represents a subjective Bayesian perspective, in which a researcher is interpreting the probability of different hypotheses, and updating their belief to the posterior probability using data. Other approaches, such as empirical Bayes in which the prior may be estimated from the data, are also possible. It should be noted that most Bayesian practitioners will use some tools from both schools of thought.**

# Reviewer report: Reviewer response for version 3

**DOI:** 10.21956/openreseurope.19757.r46205

**Published:** 2024-12-02

**Authors:** Bruna M. Farina

**Affiliations:** 1 Swiss Institute of Bioinformatics, Lausanne, Vaud, Switzerland; 2 University of Fribourg, Fribourg, Switzerland

I appreciate the opportunity to review this manuscript. In this work, the authors provide detailed instructions and guidance on how to better perform Bayesian phylogenetic inference using Markov chain Monte Carlo (MCMC). I really enjoyed how exhaustively (in a good way!) the authors explored the topic, providing very useful tips for both beginners and experts in Bayesian analyses, while keeping the explanations clear and easy to understand.

 I only have a few minor comments, listed below, but I want to highlight that these are merely suggestions, and the authors are free to incorporate them or not.

  

1. **Page 2:** I would suggest including “RevBayes” in the keywords as well.
2. **Page 4:** *“The algorithm for MCMC sampling most frequently employed in phylogenetic studies is known as the Metropolis-Hastings (MH) algorithm (Figure 2), though others exist.”. *I recommend mentioning other algorithms and stating—either here or in Section 2.1.5, wherever it flows better—why MH is the most commonly used algorithm in phylogenetics compared to alternatives, like Gibbs sampling.
3. **Page 6: Section 2.1.5 – **see comment 2 above.
4. **Page 8: Section 2.2.2 – **in this section you present the three softwares (BEAST2, RevBayes, and MrBayes). It might be helpful to include a more explicit comparison between them, discussing their advantages or why one might prefer one over the others. For instance, RevBayes is highly flexible and powerful, but it requires some scripting (although there are many tutorials available to help users).
5. **Page 16: Section 5.1.3 –** *“…* *poorly chosen temperatures can lead to inefficient mixing and other issues, and should be looked out for (Brown & Thomson, 2018).” *I think it is worth briefly mentioning other potential issues might be useful since MCMCMC is MrBayes’ default (and widely used).
6. **Page 16 – 18: Section 5.2.1–** Consider defining “hyperprior” as well. The user may encounter this term when running their analyses, so providing a brief definition, along with advice and explanations on why hyperpriors are used in hierarchical models, could be beneficial.
7. Not necessary for understanding the text (it’s already super clear), but it would be nice to include a figure illustrating how an informative prior can narrow down the parameter space compared to a vague, uninformative prior.

Is the rationale for developing the new method (or application) clearly explained?

Yes

Is the description of the method technically sound?

Yes

Are the conclusions about the method and its performance adequately supported by the findings presented in the article?

Yes

If any results are presented, are all the source data underlying the results available to ensure full reproducibility?

No source data required

Are sufficient details provided to allow replication of the method development and its use by others?

Yes

Reviewer Expertise:

Macroevolution, paleobiology

I confirm that I have read this submission and believe that I have an appropriate level of expertise to confirm that it is of an acceptable scientific standard.

# Response

**Published:** 2025-12-09

**Authors:** Joëlle Barido-Sottani

**Affiliations:** Institute of Biology, Ecole Normale Superieure, Paris, Île-de-France, France

**Thank you very much for the review and the comments! Please find our detailed response to your comments below (in bold).**

 1. Page 2: I would suggest including “RevBayes” in the keywords as well. **Added**

 2. Page 4: “The algorithm for MCMC sampling most frequently employed in phylogenetic studies is known as the Metropolis-Hastings (MH) algorithm (Figure 2), though others exist.”. I recommend mentioning other algorithms and stating—either here or in Section 2.1.5, wherever it flows better—why MH is the most commonly used algorithm in phylogenetics compared to alternatives, like Gibbs sampling.

**We now mention slice sampling as an alternative algorithm, but we found very little discussion of why MH is so popular so we left the rest of the text as is.**

 3. Page 6: Section 2.1.5 – see comment 2 above. **Please see our response above.**

 4. Page 8: Section 2.2.2 – in this section you present the three softwares (BEAST2, RevBayes, and MrBayes). It might be helpful to include a more explicit comparison between them, discussing their advantages or why one might prefer one over the others. For instance, RevBayes is highly flexible and powerful, but it requires some scripting (although there are many tutorials available to help users).

**Thank you for the suggestion. We have added some information on the different frameworks and what type of use they are best suited for.**

 5. Page 16: Section 5.1.3 – “. . . poorly chosen temperatures can lead to inefficient mixing and other issues, and should be looked out for (Brown & Thomson, 2018).” I think it is worth briefly mentioning other potential issues might be useful since MCMCMC is MrBayes’ default (and widely used).

**We have added a note for the computing time of MC3 and a recommendation of using the parallel version of the program.**

 6. Page 16 – 18: Section 5.2.1– Consider defining “hyperprior” as well. The user may encounter this term when running their analyses, so providing a brief definition, along with advice and explanations on why hyperpriors are used in hierarchical models, could be beneficial. 

**Thank you for suggesting this addition. We now include a short paragraph explaining what hyperpriors are and how they are used.**

 7. Not necessary for understanding the text (it’s already super clear), but it would be nice to include a figure illustrating how an informative prior can narrow down the parameter space compared to a vague, uninformative prior.

**Good point, but instead of adding another figure to this paper, we decided to make use of already existing visualizations, and thus now refer to Figure 1 in dos Reis et al. 2016 in Section 3.3 and to Figure 1 in dos Reis et al. 2015 in Section 5.2.1.**

# Reviewer report: Reviewer response for version 2

**DOI:** 10.21956/openreseurope.19285.r41769

**Published:** 2024-06-29

**Authors:** Daniel Casali

**Affiliations:** 1 Universidade de São Paulo, São Paulo, Brazil

I do not have any further comments.

Is the rationale for developing the new method (or application) clearly explained?

Yes

Is the description of the method technically sound?

Yes

Are the conclusions about the method and its performance adequately supported by the findings presented in the article?

Yes

If any results are presented, are all the source data underlying the results available to ensure full reproducibility?

No source data required

Are sufficient details provided to allow replication of the method development and its use by others?

Yes

Reviewer Expertise:

Systematics (phylogenetics and taxonomy), morphology, and evolutionary biology.

I confirm that I have read this submission and believe that I have an appropriate level of expertise to confirm that it is of an acceptable scientific standard.

# Reviewer report: Reviewer response for version 1

**DOI:** 10.21956/openreseurope.18012.r36509

**Published:** 2024-06-03

**Authors:** Sungsik Kong

**Affiliations:** 1 University of Wisconsin-Madison, Madison, Wisconsin, USA

Title: Practical guidelines for Bayesian phylogenetic inference using Markov Chain Monte Carlo (MCMC)

 Authors: Barido-Sottani J, Schwery O, Warnock RCM, Zhang C, and Wright AM

 Summary:

 The manuscript by Barrido-Sottani et al. offers practical guidelines for biologists conducting phylogenetic analysis using softwares that implement Bayesian frameworks, namely MrBayes, RevBayes, and BEAST2. The authors focus on Markov chain Monte Carlo (MCMC) that is used in Bayesian phylogenetic inference, and present how to diagnose some common problems and how to troubleshoot them, how to fine-tune parameters for to achieve convergence, and unique challenges when fossil information is incorporated. Overall, the manuscript is in a great shape, very well explained without using unnecessary jargons. I believe this manuscript is well-suited for introductory read for understanding MCMC in Bayesian phylogenetic inference to general audience. Below, I tried to point out any place that can be clarified where a novice to Bayesian phylogenetics (or phylogenetics in general) can possibly get confused.

 Comments:

 [1] MCMC: Markov Chain Monte Carlo → Markov chain Monte Carlo, unless intended.

 [2] Keywords: May be include RevBayes as well?

 [3] Abstract: (1) It would be more accurate to say that “estimating a phylogenetic tree involves evaluating many possible hypotheses”, instead of solutions? If the authors meant parameter estimates by the `solutions', it should be clarified.

 [4] Introduction to MCMC Paragraph 1: If possible, providing some numbers to demonstrate the vastly large number of possible topologies for given number of taxa n would help readers to admit that it is not feasible to evaluate all possible topologies (even when n is not large). For example, there are < 34 million topologies when n = 10 (Degnan and Salter, 2005, Evolution, 59(1), p.34).

 [5] Introduction to MCMC P3: (1) Try to avoid begin a sentence with an abbreviation (i.e., ML, sentence 2). (2) Plus, it would be good idea to provide some references of the mathematical techniques in sentence 2. (3) Also, the concept of prior distribution is briefly introduced here, and it would be nice to direct reader that the concept is described in more detail in the coming section (i.e., 2.1.2?.)

 [6] Introduction to MCMC P4: (1) Please mention some examples (or references) of MCMC sampling algorithms other than MH. (2) The authors mention that the one or more model parameters are perturbed, like making a number a little bigger. It would be clearer if stated as “a value of the parameter is increased''? (3) The connection between Monte Carlo and pseudorandom parameter perturbation is not obvious. (4) The concept of ‘parent solution’ should have been introduced, possibly when a ‘starting set of values’ was introduced in the beginning of this paragraph?

 [7] Introduction to MCMC P5: Provide reference for the original MCMC in early 1950s?

 [8] Section 2.1: I like “The Bayesics.” Just a suggestion, but unless intended, how about listing the three quantities in the order of apparance in the text (i.e., the likelihood, the prior, and the marginal probability)?

 [9] Figure 1: The cartoon illustration of Bayes’ theorem looks great. (1) Just make sure to keep the capitalization consistent (Bayes’ theorem vs. Bayes Theorem (in text)). (2) It would be more helpful if both top and bottom (i.e., not the second panel) are mentioned in the bolded portion of the caption. (3) Mention the asterisk (∗) represents new parameter values in the caption. (3) I think using either one of Hastings ratio or the posterior odds ratio in the figure itself would remove unnecessary confusion, and mention that both refer to the same thing in the caption.

 [10] Section 2.1.1 P1: ML for maximum likelihood is already introduced previously. No need to redo it here.

 [11] Section 2.1.2 P1: (1)Use ML since it has been introduced previously. (2) “...than one that is very short → low?”

 [12] Section 2.1.4 P2: Provide reference for reversible MCMC. Possibly provide an example for its application (e.g., phylogenetic network inference?).

 [13] Section 2.1.5 P2: There is no section called Introduction, but Introduction to MCMC.

 [14] Figure 2: I am not sure it it is necessary to include both flowchart and pseudocode. I feel like they are redundant and either one of them should be enough to explain MH. It is up to the authors to choose which one. Furthermore, in the flowchart, it would be more clear to say that the changes that decrease the posterior is accepted at probability of R, instead of vague ’Occasionally’. In pseudocode, possibly add a line somewhere that explains the variable j.

 [15] Section 2.2.1 P1: (1) The definition of unrooted trees should be more explicit by including the concept of lack of evolutionary directionality (i.e., unable to identify the ancestor–descent relationship between the nodes). (2) It would also be important to specify the trees where branch lengths represent genetic distance are dated only if the constant molecular clock assumption is met.

 [16] Section 2.2.1 P2: (1) Could you define outgroup samples in simple terms? (2) May be use the term ‘ingroup’ instead of “main clade”, because the main clade can be a portion of ingroup taxa.

 [17] Section 2.2.3 P1: (1) Are the terms 'moves' and 'operators' synonyms? They are being used interchangeably. If so, it might be a good idea to state that they are the same thing upfront (i.e., move the second last sentence to the top) and stick with one of them throughout the section. (2) I don’t think “Scaling move” is not explained in the text at this point, or direct readers to Figure 3.

 [18] Section 2.2.3 P2: The last sentence of this paragraph that begins with “This is also the reason...” seems to be very important for the readers when comparing results from different implementations. Could you add a couple sentences with an example?

 [19] Section 2.2.3 P3: Two abbreviations STX and SPR were never defined in text. Also, it would be useful to direct readers to some literature where the details of these tree moves are explained.

 [20] Section 2.2.3 P4: Please explain what it means by “satisfying” results. This may lead to the subjectivity issue.

 [21] Figure 3: (1) Please explicitly define, at least in caption, the notations used in the figure: a, s, d, etc., in scaling and sliding moves, for example. U for Uniform distribution? (2) I thought subtree exchange moves swaps the two points in two different branches in the tree (Vaughan et al,2014) [Ref 1]. Currently, it looks like as if two leaves, blue and orange, are swapped and no branch plays a role in this move. Is this what STX really does? Otherwise, the graphical representation may be misleading and confusing. Similar issue in SPR move.

 [22] Section 3: “Posterior values” → "posterior probabilities" for a set of parameter values?

 [23] Section 3.1 P1: Refer to Figure 3 when explaining the moves involving numerical change.

 [24] Section 3.1 P2: (1) “networks” → phylogenetic networks; It would be good idea to define phylogenetic networks or provide citations where the networks are defined and distinction between the trees and networks (e.g., Kong S, Et al, 2022 [Ref2] In the last sentence, it might be more accurate to say ‘topology moves’ rather than “tree moves” since the concept of networks is introduced.

 [25] Section 3.2 P1: I feel like the first sentence (“Biology is complex...”) is repeating what has been said previously.

 [26] Section 3.3: This section is very clear and informative. Beautifully written!

 [27] Section 4.1 P1: While the proportion of burn-in might not (or might) directly influence MCMC inference result (particularly if ran long enough), could you elaborate on the consequences of setting the proportion of burn-in either too small or large? For starters, setting this value may seem arbitrary and difficult to decide.

 [28] Section 4.3 P1: May be I missed, what do you mean by the autocorrelation time τ? How is it measured?

 [29] Figure 4: The description of left and right panels are well explained in the caption. However, it would be great what the different rows represent is described. (i.e., top row being not coverged vs. bottom row being the converged “hairy caterpillar”?

 [30] Section 5.1.1 P4: The term “acceptance ratios” was used. Is this synonymous to “acceptance proportion” mentioned in section 2? If so, please mention it or keep these terms consistent.

 [31] Section 5.1.1 P7: The abbreviation “DAG” is introduced, but never used again in the manuscript. May be unnecessary.

 [32] Section 5.2.2 P2: “...help a researcher determine” → ‘...help a researcher to determine’?

Is the rationale for developing the new method (or application) clearly explained?

Partly

Is the description of the method technically sound?

Yes

Are the conclusions about the method and its performance adequately supported by the findings presented in the article?

Yes

If any results are presented, are all the source data underlying the results available to ensure full reproducibility?

No source data required

Are sufficient details provided to allow replication of the method development and its use by others?

Yes

Reviewer Expertise:

Phylogenetic method development; phylogenetic networks; hybridization

I confirm that I have read this submission and believe that I have an appropriate level of expertise to confirm that it is of an acceptable scientific standard.

## References

[1] : Efficient Bayesian inference under the structured coalescent. *Bioinformatics* .2014;30(16) : 10.1093/bioinformatics/btu2012272-9 24753484 10.1093/bioinformatics/btu201 PMC4207426

[2] : Classes of explicit phylogenetic networks and their biological and mathematical significance. *J Math Biol* .2022;84(6) : 10.1007/s00285-022-01746-y47 35503141 10.1007/s00285-022-01746-y

# Response

**Published:** 2024-07-29

**Authors:** Joëlle Barido-Sottani

**Affiliations:** Institute of Biology, Ecole Normale Superieure, Paris, Île-de-France, France

Thank you very much for the review and the comments! Please find our detailed response (bold text) below.

1. MCMC: Markov Chain Monte Carlo → Markov chain Monte Carlo, unless intended. - **Fixed.**
2. Keywords: May be include RevBayes as well? - **Fixed.**
3. Abstract: (1) It would be more accurate to say that “estimating a phylogenetic tree involves evaluating many possible hypotheses”, instead of solutions? If the authors meant parameter estimates by the ‘solutions’, it should be clarified. - **We have clarified this sentence.**
4. Introduction to MCMC Paragraph 1: If possible, providing some numbers to demonstrate the vastly large number of possible topologies for given number of taxa n would help readers to admit that it is not feasible to evaluate all possible topologies (even when n is not large). For example, there are > 34 million topologies when n = 10 (Degnan and Salter, 2005, Evolution, 59(1), p.34). - **We have added the suggested example.**
5. Introduction to MCMC P3: (1) Try to avoid begin a sentence with an abbreviation (i.e., ML, sentence 2). - **Fixed.**
  (2) Plus, it would be good idea to provide some references of the mathematical techniques in sentence 2. - **Added.**
  (3) Also, the concept of prior distribution is briefly introduced here, and it would be nice to direct reader that the concept is described in more detail in the coming section (i.e., 2.1.2?.) - **As suggested, we now mention that priors are discussed in details in the following sections.**
6. Introduction to MCMC P4: (1) Please mention some examples (or references) of MCMC sampling algorithms other than MH. - **We have added information on slice sampling in this paragraph. As suggested by another reviewer, we also now give examples of alternatives to MCMC in the beginning of the section.**
  (2) The authors mention that the one or more model parameters are perturbed, like making a number a little bigger. It would be clearer if stated as “a value of the parameter is increased”? - **We have edited as suggested.**
  (3) The connection between Monte Carlo and pseudorandom parameter perturbation is not obvious. - **We have edited this part to clarify the connection.**
  (4) The concept of ‘parent solution’ should have been introduced, possibly when a ‘starting set of values’ was introduced in the beginning of this paragraph? - **As suggested by another reviewer, we replaced all mentions of ’parent solution’ by ’current solution’, which we hope makes this part clearer.**
7. Introduction to MCMC P5: Provide reference for the original MCMC in early 1950s? - **We have added this reference.**
8. Section 2.1: I like “The Bayesics.” Just a suggestion, but unless intended, how about listing the three quantities in the order of appearance in the text (i.e., the likelihood, the prior, and the marginal probability)?  - **The order of quantities has been changed as suggested.**
9. Figure 1: The cartoon illustration of Bayes’ theorem looks great. (1) Just make sure to keep the capitalization consistent (Bayes’ theorem vs. Bayes Theorem (in text)). - **The text now consistently uses Bayes’ theorem.**
  (2) It would be more helpful if both top and bottom (i.e., not the second panel) are mentioned in the bolded portion of the caption. - **We have added a global title for the figure in the caption.**
  (3) Mention the asterisk (*) represents new parameter values in the caption. - **Added.**
  (4) I think using either one of Hastings ratio or the posterior odds ratio in the figure itself would remove unnecessary confusion, and mention that both refer to the same thing in the caption. - **We have removed the mention of posterior odds ratio from the figure.**
10. Section 2.1.1 P1: ML for maximum likelihood is already introduced previously. No need to redo it here. - **Fixed.**
11. Section 2.1.2 P1: (1)Use ML since it has been introduced previously. (2) “...than one that is very short → low?” - **Fixed.**
12. Section 2.1.4 P2: Provide reference for reversible MCMC. Possibly provide an example for its application (e.g., phylogenetic network inference?). - **We have added a reference and an example of application to the selection of substitution models.**
13. Section 2.1.5 P2: There is no section called Introduction, but Introduction to MCMC. - **Fixed.**
14. Figure 2: I am not sure it it is necessary to include both flowchart and pseudocode. I feel like they are redundant and either one of them should be enough to explain MH. It is up to the authors to choose which one. Furthermore, in the flowchart, it would be more clear to say that the changes that decrease the posterior is accepted at probability of R, instead of vague ’Occasionally’. In pseudocode, possibly add a line somewhere that explains the variable j. - **Our intention is that the flowchart presents the principle of the algorithm, whereas the pseudocode includes the technical details of the calculations performed at each step. As a result, we do not feel that these two parts are redundant and have chosen to leave them both in the figure. We now include some details on the variables in the caption.**
15. Section 2.2.1 P1: (1) The definition of unrooted trees should be more explicit by including the concept of lack of evolutionary directionality (i.e., unable to identify the ancestor–descent relationship between the nodes). - **As suggested, we have added this information about unrooted trees.**
  (2) It would also be important to specify the trees where branch lengths represent genetic distance are dated only if the constant molecular clock assumption is met. - **Here we define dated trees as trees with branch lengths in terms of time, so genetic distance trees are undated by definition.**
16. Section 2.2.1 P2: (1) Could you define outgroup samples in simple terms? - **We have added a definition and some examples for outgroup samples.**
  (2) Maybe use the term ‘ingroup’ instead of “main clade”, because the main clade can be a portion of ingroup taxa. - **We have removed the references to the “main clade” from this section.**
17. Section 2.2.3 P1: (1) Are the terms ’moves’ and ’operators’ synonyms? They are being used interchangeably. If so, it might be a good idea to state that they are the same thing upfront (i.e., move the second last sentence to the top) and stick with one of them throughout the section.  - ​​​​​​​ **Yes, the terms “moves”, “operators” and “proposals” generally refer to the same components, but are used in different software communities. We have edited the text to make this clearer.**
  (2) I don’t think “Scaling move” is not explained in the text at this point, or direct readers to Figure 3. - **This reference to scaling moves has been removed.**
18. Section 2.2.3 P2: The last sentence of this paragraph that begins with “This is also the reason...” seems to be very important for the readers when comparing results from different implementations. Could you add a couple sentences with an example? - **We have added more details on why numbers of iterations cannot be directly compared between implementations.**
19. Section 2.2.3 P3: Two abbreviations STX and SPR were never defined in text. Also, it would be useful to direct readers to some literature where the details of these tree moves are explained. - **The abbreviations are now spelled out in the text. We have also added a reference to the original description of these moves in the caption to Figure 3.**
20. Section 2.2.3 P4: Please explain what it means by “satisfying” results. This may lead to the subjectivity issue. - **We have added more details in this sentence.**
21. Figure 3: (1) Please explicitly define, at least in caption, the notations used in the figure: a, s, d, etc., in scaling and sliding moves, for example. U for Uniform distribution? - **We have added these definitions in the caption.**
  (2) I thought subtree exchange moves swaps the two points in two different branches in the tree (Vaughan et al,2014) [Ref 1]. Currently, it looks like as if two leaves, blue and orange, are swapped and no branch plays a role in this move. Is this what STX really does? Otherwise, the graphical representation may be misleading and confusing. Similar issue in SPR move. - **The coloured triangles were intended to represent subclades with any number of branches and leaves, rather than just leaves alone. We now make this clear in the figure caption.**
22. Section 3: “Posterior values” → ”posterior probabilities” for a set of parameter values? - **Fixed.**
23. Section 3.1 P1: Refer to Figure 3 when explaining the moves involving numerical change. - **Added.**
24. Section 3.1 P2: (1) “networks” → phylogenetic networks; It would be good idea to define phylogenetic networks or provide citations where the networks are defined and distinction between the trees and networks (e.g., Kong S, Et al, 2022 [Ref2]) - ​​​​​​​ **We have edited as suggested and added the reference.**
  (2) In the last sentence, it might be more accurate to say ‘topology moves’ rather than “tree moves” since the concept of networks is introduced. - **Fixed.**
25. Section 3.2 P1: I feel like the first sentence (“Biology is complex...”) is repeating what has been said previously. - **We have edited this section to remove the repetition.**
26. Section 3.3: This section is very clear and informative. Beautifully written! - **Thank you !**
27. Section 4.1 P1: While the proportion of burn-in might not (or might) directly influence MCMC inference result (particularly if ran long enough), could you elaborate on the consequences of setting the proportion of burn-in either too small or large? For starters, setting this value may seem arbitrary and difficult to decide. - **We have added some details in this section.**
28. Section 4.3 P1: Maybe I missed, what do you mean by the autocorrelation time τ? How is it measured? - **We have added a definition of τ and information on how it is calculated.**
29. Figure 4: The description of left and right panels are well explained in the caption. However, it would be great what the different rows represent is described. (i.e., top row being not converged vs. bottom row being the converged “hairy caterpillar”)  - **We have expanded the caption to Figure 4 to better describe the different scenarios.**
30. Section 5.1.1 P4: The term “acceptance ratios” was used. Is this synonymous to “acceptance proportion” mentioned in section 2? If so, please mention it or keep these terms consistent. - **We have changed the word ratio to rate, and we now clarify that acceptance proportion and acceptance ratio are used interchangeably in the literature.**
31. Section 5.1.1 P7: The abbreviation “DAG” is introduced, but never used again in the manuscript. May be unnecessary. - **We have chosen to keep the abbreviation as it is used frequently in other articles discussing RevBayes.**
32. Section 5.2.2 P2: “...help a researcher determine” → ‘...help a researcher to determine’? - **Fixed.**

# Reviewer report: Reviewer response for version 1

**DOI:** 10.21956/openreseurope.18012.r37734

**Published:** 2024-03-12

**Authors:** Grégoire Clarté

**Affiliations:** 1 University of Helsinki, Helsinki, Finland

This paper summarises the current knowledge about Bayesian phylogenetic inference with a particular focus on numerical methods. The paper is particularly welcome as this problem is particularly complex and frequent in numerous applications. No new method is presented, but the article offers a nice presentation of the most common method and their implementation. It contains also a general workflow for carrying Bayesian Phylogenetical Inference.

 Overall, I think some choices are strange (for example the strange notations for parameters and observations in Fig. 1). These choices must be explained by the readers having little mathematical background. Nevertheless, I think this is not a good idea, as the article should aim at giving the mathematical tools needed to the reader, and I don't think ideographic representation gives better understanding than proper definitions (I needed some time to understand the images). Introducing proper notations would also allow to discuss the notion of ``non informative priors'' and more importantly conjugated priors that would help the choice of priors. It can also be beneficial to allow the users to communicate with the computational statisticians that developed the tools.

 For example, in 2.1.6. I think the article would benefit from mentioning the standard results of Markov Chain theory (that is ergodicity of the chain). This in return would be of interest when mentioning the different moves that can be used in MCMC algorithm, and could give insight as for possible reasons of failures.

 The part about the different problems of MCMC methods is well written and contains all the important information in my opinion.

 I disagree with section 5.1.2, or I think the phrasing is strange: the choice of a fixed initial value can be misleading (see later the case of poorly ergodic chains because of multimodality). I think the choice of a random initialisation from the prior along with repeated runs should be the correct recommendation (along with more careful observation of convergence).

 I also have some doubts on the diagnostic section: this problem is one of the most difficult in phylogenetics, for the reason that the parameters of the model (that include the topology) cannot be all summarised by one ESS. Overall all the existing methods are inefficient (but once again that's a review of the numerical methods that exists, and these inefficient methods are the only ones implemented). It would be interesting to the reader to see what are the possible issues that arises from using the ESS in the phylogenetic case (which itself has to be defined, as the author mention). Nevertheless, I am happy to see the part about the trace observation (which should be the first check run by any user of an MCMC method).

 I am a little surprised the authors do not mention earlier the most simple method to detect multimodality: running several chains in parallel. If the chains converge to different trees, that indicates that further study is required. In particular the use of standard MCMC such as the ones presented in the paper (MrBeast, etc.) will not be able to handle these problems. In my field this is a very frequent case of failure of the MCMC. This is mentioned later in the paper but it would be beneficial to discuss that earlier. On the question of detecting convergence, the remarks of 5.4 can have links with the results on MCMC convergence on trees developed following the works of Biswas, N., Jacob, P. E. and Vanetti, P. (2019) *Estimating convergence of Markov chains with L-lag couplings* (NeurIPS 7389–7399). These methods are not implemented in the current software but they have the advantage of being theoretically sound, and have been applied to phylogenetics. Of course, this is out of the scope of the article if we consider that the users will only make use of the already implemented tools.

 A problem I can see, is that the article mentions problems that cannot be solved in most of the software mentioned unless the user has a good knowledge of them. For example, if there are mixing problems for some parameters, this can be a problem of correlation between the parameters, and a move in the joint space would be recommended, which is unfortunately not possible to implement easily in most cases.

 Finally, I am wondering if mentioning more complex but more efficient methods such as SMC for trees (for example the works from Bouchard-Côté) can be interesting. My idea is that if there are problems with the standard methods, the user should move on to more complex numerical methods, although they are not implemented in a user friendly way.

 As a conclusion, I would say that the article is good but requires some polishing depending on the goals the authors have. It obviously is tailored for non-statisticians that are using Bayesian phylogenetics tools. It does a great job at presenting the problems, the tools, and the possible solutions (I am happy to see the recommendation to discuss complex problems on the forums) but I still think stronger maths would be beneficial.

 Minor comments:

- "2.1 The Bayesics" I'm not sure the pun is needed in this kind of article (also, it can be difficult to understand for non native english speakers that would pronounce Bayes differently).
- I have philosophical problems with the notion of "choosing" the prior, as the prior is not chosen by the user but just exists out of the general knowledge (as is described by the paper). I think the author could mention repeated experiments with different priors to ensure the prior effect is negligible with respect to the results.
- I use more often marginal likelihood than marginal probability to designate the integrate of the likelihood times the prior, I don't know what are the terms used in the phylogenetics community.

Is the rationale for developing the new method (or application) clearly explained?

Yes

Is the description of the method technically sound?

Yes

Are the conclusions about the method and its performance adequately supported by the findings presented in the article?

Yes

If any results are presented, are all the source data underlying the results available to ensure full reproducibility?

Yes

Are sufficient details provided to allow replication of the method development and its use by others?

Yes

Reviewer Expertise:

Computational bayesian phylogenetics, computational statistics, numerical methods in bayesian statistics.

I confirm that I have read this submission and believe that I have an appropriate level of expertise to confirm that it is of an acceptable scientific standard, however I have significant reservations, as outlined above.

# Response

**Published:** 2024-05-30

**Authors:** Joëlle Barido-Sottani

**Affiliations:** Institute of Biology, Ecole Normale Superieure, Paris, Île-de-France, France

**Thank you very much for the review and suggestions! Please find our detailed response below (in bold).** Overall, I think some choices are strange (for example the strange notations for parameters and observations in Fig. 1). These choices must be explained by the readers having little mathematical background. Nevertheless, I think this is not a good idea, as the article should aim at giving the mathematical tools needed to the reader, and I don’t think ideographic representation gives better understanding than proper definitions (I needed some time to understand the images). Introducing proper notations would also allow to discuss the notion of “non informative priors” and more importantly conjugated priors that would help the choice of priors. It can also be beneficial to

 allow the users to communicate with the computational statisticians that developed the tools.

**We have chosen to retain the pictures used in place of mathematical notation in Figure 1 (although, as described in response to reviewer 2, we have made the image clearer and we now include a figure legend with descriptions of the components). Our reasoning for this is that phylogenetics is perhaps unusual in that it draws a lot of users who have no formal training in mathematics, who often have limited practice or experience reading equations in any context. Formal notation appears in many fundamental textbooks and papers that introduce Bayesian phylogenetics and MCMC (e.g., Ziheng Yang’s (2006) Computational Molecular Evolution book or Joseph Felsenstein’s (2004) Inferring phylogenies book). These resources can be challenging at first for those unfamiliar with mathematical notation and new to phylogenetics. Our intention is to provide a reference that is both complementary to these resources and more accessible for beginners. This perspective is based on our experience as individual researchers**

**and our experience teaching Bayesian phylogenetics in a wide range of contexts (especially, as part of the RevBayes or BEAST2 workshops which are aimed at empirical researchers).**

**We have included some discussion on the use of non-informative or improper priors and the potential issues these can cause (section 5.2 Choice of model and priors). However, we chose not to discuss the use of conjugate priors, as it is not often possible to use these in the context of Bayesian phylogenetics. A regular phylogenetic software user would rarely (if ever) encounter this term.** For example, in 2.1.6. I think the article would benefit from mentioning the standard results of Markov Chain theory (that is ergodicity of the chain). This in return would be of interest when mentioning the different moves that can be used in MCMC algorithm, and could give insight as for possible reasons of failures.

**We have added some information on ergodicity and its implications on the MCMC moves.** The part about the different problems of MCMC methods is well written and contains all the important information in my opinion.

 I disagree with section 5.1.2, or I think the phrasing is strange: the choice of a fixed initial value can be misleading (see later the case of poorly ergodic chains because of multimodality). I think the choice of a random initialisation from the prior along with repeated runs should be the correct recommendation (along with more careful observation of convergence).

**We have edited this to make it clearer. We aren’t trying to argue for one method of doing this over another, simply to state the major classes of solutions found in the literature. To this section, we’ve added some explanation about how starting point may affect the inference, and how to detect sensitivity to starting point.** I also have some doubts on the diagnostic section: this problem is one of the most difficult in phylogenetics, for the reason that the parameters of the model (that include the topology) cannot be all summarised by one ESS. Overall all the existing methods are inefficient (but once again that’s a review of the numerical methods that exists, and

 these inefficient methods are the only ones implemented). It would be interesting to the reader to see what are the possible issues that arises from using the ESS in the phylogenetic case (which itself has to be defined, as the author mention). Nevertheless, I am happy to see the part about the trace observation (which should be the first check run by any user of an MCMC method).

**Standard convergence diagnostics in phylogenetics involve calculating one ESS per parameter that is involved in the analysis, rather than one single ESS for the whole model, as the initial text might have implied. Some of the confusion may have resulted from the later mention of the topology ESS as calculated by Convenience, since that comes closest to the idea of one single ESS for the whole analysis. We have thus edited the text to make that clearer. We have furthermore added a brief section detailing which ESS to check and what to pay attention to.** I am a little surprised the authors do not mention earlier the most simple method to detect multimodality: running several chains in parallel. If the chains converge to different trees, that indicates that further study is required. In particular the use of standard MCMC such as the ones presented in the paper (MrBeast, etc.) will not be able to handle these problems. In my field this is a very frequent case of failure of the MCMC. This is mentioned later in the paper but it would be beneficial to discuss that earlier.

**We now mention the use of multiple chains in the convergence assessment section. We have edited this part to emphasize that certain issues such as multimodality can only be detected through this method.** On the question of detecting convergence, the remarks of 5.4 can have links with the results on MCMC convergence on trees developed following the works of Biswas, N., Jacob, P. E. and Vanetti, P. (2019) Estimating convergence of Markov chains with L-lag couplings (NeurIPS 7389–7399). These methods are not implemented in the current

 software but they have the advantage of being theoretically sound, and have been applied to phylogenetics. Of course, this is out of the scope of the article if we consider that the users will only make use of the already implemented tools.

**We agree that while this may be a nascent method, it might be useful for readers to be aware of it and explore the possibilities of its use. We have thus added a mention of it to the convergence section, along with two papers by Kelly et al, who already apply the method of Biswas et al. in a phylogenetic context.** A problem I can see, is that the article mentions problems that cannot be solved in most of the software mentioned unless the user has a good knowledge of them. For example, if there are mixing problems for some parameters, this can be a problem of correlation between the parameters, and a move in the joint space would be recommended, which is unfortunately not possible to implement easily in most cases.

**We have tried to cover as many as possible solutions available in the software that users can adjust or fine-tune across appropriate sections. We now clearly state that our focus in on solutions which can be implemented by the user without additional development, but that many issues do require changes in the software itself. The problem of correlation between parameters is particularly hard to deal with, typically requiring the developers to implement efficient MCMC moves to overcome it. Nevertheless, we now mention the up-down operator in BEAST2 which will scale both the branch lengths of the tree, and the clock rate simultaneously.** Finally, I am wondering if mentioning more complex but more efficient methods such as SMC for trees (for example the works from Bouchard-Côté) can be interesting. My idea is that if there are problems with the standard methods, the user should move on to more complex numerical methods, although they are not implemented in a user friendly

 way.

**We added references to other algorithms when introducing MCMC. These are very interesting and worth discussing, but out-of-scope for us.** As a conclusion, I would say that the article is good but requires some polishing depending on the goals the authors have. It obviously is tailored for non-statisticians that are using Bayesian phylogenetics tools. It does a great job at presenting the problems, the tools, and the possible solutions (I am happy to see the recommendation to discuss complex problems on the forums) but I still think stronger maths would be beneficial. Minor comments:

 “2.1 The Bayesics” I’m not sure the pun is needed in this kind of article (also, it can be difficult to understand for non native english speakers that would pronounce Bayes differently).

**Thank you for pointing out the valid concern about non-native speakers. In consultation with the non-native English speakers among the authors, we opted to remove the pun here.** I have philosophical problems with the notion of ”choosing” the prior, as the prior is not chosen by the user but just exists out of the general knowledge (as is described by the paper). I think the author could mention repeated experiments with different priors to ensure the prior effect is negligible with respect to the results.

**We disagree with the idea that the prior should be negligible. If the prior is reflecting sound empirical information, that information should be incorporated in the analysis. But more to the point, any analysis contains many choices. Whether it is a choice of methodology, or the choice of null and alternative hypotheses, these choices are often consequential. Thus, it’s our feeling that as with any other choice, consequentiality is less important than justifiability. We have kept the phrasing of “choice” when discussing the priors, but we now mention testing different priors to explore their impact on the results and conclusions. We also now emphasise that in some contexts in Bayesian phylogenetics the parameters of interest are not fully identifiable (this is especially true for divergence time estimation) and that as a result the priors need to very carefully considered (sections 2.1.2 and 3.3).** I use more often marginal likelihood than marginal probability to designate the integrate of the likelihood times the prior, I don’t know what are the terms used in the phylogenetics community.

**We edited the first mention of the term to state that both terms are used interchangeably.**

# Reviewer report: Reviewer response for version 1

**DOI:** 10.21956/openreseurope.18012.r37731

**Published:** 2024-03-12

**Authors:** Zhirui Hu

**Affiliations:** 1 Gladstone Institute of Data Science and Biotechnology, San Francisco, California, USA

The authors provide a very thorough introduction of Bayesian analysis, MCMC for posterior inference in Bayesian phylogenetics, common issues and troubleshooting of model choices and running MCMC. The article is well-structured, informative, easy to read and technically sound. In general, I think it would be more helpful to include more examples and figures to illustrate issues and troubleshooting techniques, and/or an example illustrating the entire process from model design, MCMC convergence diagnostics and model selection or update.

 Besides, there are a few minor issues in the article:

- page 4, “between the new and parent scores”, “current” is more commonly used than “parent” to indicate the current state in the Markov chain.
- Figure 1, the cartoon illustration for phylogeny and alignment etc. is interesting but too small to read. Also, summation sign instead of integral could be used for summing over discrete variables, i.e. phylogenetic trees.
- Page 7, “a minimum of 10,000 posterior samples”, I think choosing the number of MCMC samples should depend on how correlated samples are or ESS.
- Section 2.2.3, “Scaling move the components”, is it a typo?
- Pg 9, section 3.1, the author mentioned that traversing tree space was largely solved in the 1990s but later mentioned challenges in phylogenetic inference. I think the authors need to clarify in which situation the problem was solved.
- Section 3.2, adding a figure might be helpful to illustrate the posterior space. “when calculating the prior probability of the phylogeny…”, should it be “posterior probability”? Also, what is a diversification model? Maybe add some reference here.
- Figure 4 is good to illustrate different types of trace plot, but the authors could add more explanations on Figure 4. What are the problems of the first two trace plots?
- Section 5.1.1 tuning step size is very important in MCMC. The author could provide a table or list to summarize pros and cons of large/small step size and tuning step size.
- Pg 16, posterior predictive simulations is very useful and it would be helpful if the authors can provide a toy example of it. “If the model is adequate to describe/analyse the variation in the data,…”, is it a typo? Should it be “inadequate”?
- Pg 17, any reference for ABC?

Is the rationale for developing the new method (or application) clearly explained?

Yes

Is the description of the method technically sound?

Yes

Are the conclusions about the method and its performance adequately supported by the findings presented in the article?

Yes

If any results are presented, are all the source data underlying the results available to ensure full reproducibility?

No source data required

Are sufficient details provided to allow replication of the method development and its use by others?

Yes

Reviewer Expertise:

Bayesian statistics, phylogenetics

I confirm that I have read this submission and believe that I have an appropriate level of expertise to confirm that it is of an acceptable scientific standard.

# Response

**Published:** 2024-05-30

**Authors:** Joëlle Barido-Sottani

**Affiliations:** Institute of Biology, Ecole Normale Superieure, Paris, Île-de-France, France

**Thank you very much for the review and the comments! Please find our response to the other issues below (in bold).** In general, I think it would be more helpful to include more examples and figures to illustrate issues and troubleshooting techniques, and/or an example illustrating the

 entire process from model design, MCMC convergence diagnostics and model selection or update.

**We feel that specific examples of the troubleshooting process will be too software-dependent to be helpful in the manuscript, however we have written a RevBayes tutorial illustrating many of the issues and techniques outlined here. The tutorial is now mentioned in section 6. ** Besides, there are a few minor issues in the article: page 4, “between the new and parent scores”, “current” is more commonly used than “parent” to indicate the current state in the Markov chain. **Fixed.** Figure 1, the cartoon illustration for phylogeny and alignment etc. is interesting but too small to read. Also, summation sign instead of integral could be used for summing over discrete variables, i.e. phylogenetic trees.

**We have reorganised Figure 1 to make everything larger, added a legend, and added colour to distinguish the data vs. the model (+ tree). We have also expanded the legend to include more information. We opted not to add the summation for discrete variables, since the tree is also comprised of continuous**

**branch lengths. We believe the main point we are trying to make here − that the marginal probability of the data must take into account all possible parameter values and trees − is accurately conveyed without making this expression more complicated.** Page 7, “a minimum of 10,000 posterior samples”, I think choosing the number of MCMC samples should depend on how correlated samples are or ESS.

**We have edited this section to make it clearer that the number of recorded samples is due to space and memory constraints, and that a higher number of samples is not indicative of the convergence of the chain, since as you note samples are correlated.** Section 2.2.3, “Scaling move the components”, is it a typo?

**It was indeed a typo, thank you for pointing this out.** Pg 9, section 3.1, the author mentioned that traversing tree space was largely solved in the 1990s but later mentioned challenges in phylogenetic inference. I think the authors need to clarify in which situation the problem was solved.

**This was unclear indeed. This has been clarified to ”Algorithms for efficiently sampling phylogenetic tree space became available in the late 1990s“** Section 3.2, adding a figure might be helpful to illustrate the posterior space.

**We believe that adding a figure would make this section too long. However, we have added in the introduction links to several online tools which allow users to gain a better intuition for posterior spaces and how the MCMC algorithm works.** “when calculating the prior probability of the phylogeny. . . ”, should it be “posterior probability”?

**We have removed the word “prior” as the probability of the phylogeny can be part of the prior or the likelihood depending on the analysis.** Also, what is a diversification model? Maybe add some reference here.

**Following a suggestion by Reviewer 1, we now briefly introduce diversification models in section 2.1.1 and give a reference. We hope that this clarifies the text here.** Figure 4 is good to illustrate different types of trace plot, but the authors could add more explanations on Figure 4. What are the problems of the first two trace plots?

**We have added clarifying comments to the caption of this, pointing to diagnostic features of the problems shown.** Section 5.1.1 tuning step size is very important in MCMC. The author could provide a table or list to summarize pros and cons of large/small step size and tuning step size.

**We agree that these are important elements of running an MCMC, but feel that our treatment of the pros and cons of them in this section would be sufficient. We thought that adding an extra table only to summarize this information again would not add all that much clarity. Instead, since we are already demonstrating the outcomes of different stepsizes and tuning parameters in Figure 4, we have added a reference to the figure in the respective parts of this section.** Pg 16, posterior predictive simulations is very useful and it would be helpful if the authors can provide a toy example of it.

**We agree that toy examples would be helpful, but we believe that this would be covered better by software-specific tutorials. We now link to such a tutorial on the RevBayes website.** “If the model is adequate to describe/analyse the variation in the data,. . . ”, is it a typo? Should it be “inadequate”?

**This was indeed a typo and we have changed it to “inadequate”.** Pg 17, any reference for ABC? **Added.**

# Reviewer report: Reviewer response for version 1

**DOI:** 10.21956/openreseurope.18012.r36973

**Published:** 2024-02-21

**Authors:** Daniel Casali

**Affiliations:** 1 Universidade de São Paulo, São Paulo, Brazil

Dear editor and authors,

 The manuscript prepared by Barido-Sottani et al., entitled “Practical guidelines for Bayesian phylogenetic inference using Markov Chain Monte Carlo (MCMC)”, begins providing a concise, but also detailed, review of Markov Chain Monte Carlo (MCMC) sampling procedure, widely used in Bayesian phylogenetic inferences. The main focus of the text then moves to assessing the practical performance of this tool, particularly with respect to convergence issues commonly encountered by researchers.

 Despite being a common problem, failing to achieve convergence in Bayesian analyses is anything but a trivial matter, and, until now, to the best of my knowledge, there has been no study that delves so exhaustively and directly into this issue. The manuscript, therefore, constitutes an invaluable contribution to the field of study, useful not only to undergraduate and graduate students embarking on these analyses for the first time, but also to more experienced users.

 The article spans from more technical issues, such as improving the operators/movements used to propose new values during the MCMC sampling progression, to other practical aspects, such as defining well-behaved priors, selecting and critically evaluating models applied in inferences, and understanding dataset characteristics that can lead to performance issues. Useful guidelines are provided on how to initiate an analysis with good chances to converge, as well as how to address common issues like sampling from multiple optima, chain mixing problems, among others. In addition, it provides directions for asking for help in cases where all other solutions have failed. In sum, this article constitutes a very useful source of information for all those interested in the practical aspects of running phylogenetic Bayesian analyses.

 Below, I make a few minor suggestions that I believe could enhance the article's utility for its target audience. However, I emphasize that these are only small changes, and I leave it to the authors' discretion to incorporate all or any of them.

1. Page 4. “…this ensures that we explore the entire parameter space and do not stay stuck in a local optimum.” **A somewhat pedantic observation here: The *entire* parameter space cannot be ensured to be explored. Perhaps replace by something like: "we broadly explore the parameter space"?**
2. Page 4. “Invented in the early 1950’s, MCMC was originally used in physics to describe equilibrium between the liquid and gas phases of a chemical.” **I recommend a citation here, for readers interested in the history of the method itself.**
3. Page 5. “…a substitution model, which describes the relative rate of change from one character to another, and…”. **I suggest to add also: “...and the relative rate of change among character states.**
4. **P**age 5. “Under this prior, a rate that is very high is believed to be less likely than one that is very [- short] **low**.
5. Page 6. “Different estimation methods have been developed to approximate the marginal likelihood, such as path sampling (Baele et al., 2012)[Ref-7] or nested sampling (Russel et al., 2018), but they remain expensive”. **Even though, technically, stepping stones sampling is a kind of path sampling procedure, I would specifically mention stepping stones separately here as well, since is the most widely used method for estimate marginal likelihoods. Also, in the same sentence, I consider that it would be informative to indicate the term “marginal likelihood” is synonymous with the other term more consistently used in the paper, marginal probability.**
6. Page 7. “…We typically record the state of the chain with a frequency that results in a minimum of 10,000 posterior samples.”. **But probably less than that, if we perform many moves per generation, as in RevBayes?**
7. Page 7. “Another important feature of phylogenies is whether they are dated, i.e., whether their branch lengths are expressed in units of genetic/ **morphological **distance or in units of time.” and “Thus we mainly target this article at analyses which include a molecular **/morphological** clock…”.
8. Page 7. Estimating a dated phylogeny requires a model for the molecular or morphological clock, **a model of lineage diversification,** as well as time information to calibrate the tree.
9. Page 7. Thus, Scaling **moves** the components designed to advance the chain **and** are a core part of any MCMC inference software.
10. Page 10. “In practice, however, character data is not available or limited for most groups…” **As an alternative here, continuous morphological characters could be used in total-evidence analyses (e.g.,** **Álvarez-Carretero et al. (2019)[Ref-1], Zhang et al. (2021)[Ref-2]in press). These could be more readily available for some taxonomic groups, although the performance of including these characters need to be carefully considered (Varón-González et al. (2020)[Ref-3].**
11. Page 12 *. 5.1 Inference technical setup.* **Here I missed some mentioning of MCMCMC and the use of heated chains or adjusting chain temperature values to try to improve convergence. I guess this is a central topic in the current subject, that should be briefly mentioned by the authors.**
12. Page 12.” If an operator is missing…”. **Maybe the authors could emphasize here that this is a more relevant issue (as far as I can see) in RevBayes, in which we are "freer" to customize the inclusion of operators (or anything else, basically!).**
13. Page 14. “..and with added constraints such as [ - monophyletic] subclades.”. **Clades (or subclades) are monophyletic by definition.**
14. **P**age 15. *5.2.1 Priors*. **Here I missed some advice on avoiding the use of improper priors, as in some of the Beast2 default settings. These improper priors can also lead to convergence issues sometimes.**
15. Page 18. ” …partition morphological character data”. **Although only weakly related to the main theme of the paper,** **I think other methods of morphological data partitioning and what we know about their performances (which is little, if compared to dna…) could be briefly mentioned here, to give a broader picture to the reader (e.g., Clarke & Middleton (2008)[Ref-4], Rosa et al. (2019)[Ref-5], Casali et al. (2023)[Ref-6]).**

My best regards,

 Daniel Casali

 P.S. The first three questions presented in the peer review form (all answered “YES”), actually do not apply to this study, because no new method is proposed. The paper, although a methods paper, is more of a review and a practical guide for troubleshooting problems in Bayesian phylogenetic analyses.

Is the rationale for developing the new method (or application) clearly explained?

Yes

Is the description of the method technically sound?

Yes

Are the conclusions about the method and its performance adequately supported by the findings presented in the article?

Yes

If any results are presented, are all the source data underlying the results available to ensure full reproducibility?

No source data required

Are sufficient details provided to allow replication of the method development and its use by others?

Yes

Reviewer Expertise:

Systematics (phylogenetics and taxonomy), morphology, and evolutionary biology.

I confirm that I have read this submission and believe that I have an appropriate level of expertise to confirm that it is of an acceptable scientific standard.

## References

[1] : Bayesian Estimation of Species Divergence Times Using Correlated Quantitative Characters. *Syst Biol* .2019;68(6) : 10.1093/sysbio/syz015967-986 30816937 10.1093/sysbio/syz015

[2] : Fast Bayesian inference of phylogenies from multiple continuous characters. *bioRxiv* .2021; 10.1101/2021.04.21.440863 10.1101/2021.04.21.440863 PMC1112959638085256

[3] : Estimating Phylogenies from Shape and Similar Multidimensional Data: Why It Is Not Reliable. *Syst Biol* .2020;69(5) : 10.1093/sysbio/syaa003863-883 31985800 10.1093/sysbio/syaa003

[4] : Mosaicism, modules, and the evolution of birds: results from a Bayesian approach to the study of morphological evolution using discrete character data. *Syst Biol* .2008;57(2) : 10.1080/10635150802022231185-201 18404577 10.1080/10635150802022231

[5] : Homoplasy-Based Partitioning Outperforms Alternatives in Bayesian Analysis of Discrete Morphological Data. *Syst Biol* .2019;68(4) : 10.1093/sysbio/syz001657-671 30649562 10.1093/sysbio/syz001

[6] : Evaluating the Impact of Anatomical Partitioning on Summary Topologies Obtained with Bayesian Phylogenetic Analyses of Morphological Data. *Syst Biol* .2023;72(1) : 10.1093/sysbio/syac07662-77 36472372 10.1093/sysbio/syac076

[7] : Accurate model selection of relaxed molecular clocks in bayesian phylogenetics. *Mol Biol Evol* .2013;30(2) : 10.1093/molbev/mss243239-43 23090976 10.1093/molbev/mss243 PMC3548314

# Response

**Published:** 2024-05-30

**Authors:** Joëlle Barido-Sottani

**Affiliations:** Institute of Biology, Ecole Normale Superieure, Paris, Île-de-France, France

Thank you very much for the review and the comments! Please find our detailed response (bold text) below. 1. Page 4. “. . . this ensures that we explore the entire parameter space and do not stay stuck in a local optimum.” A somewhat pedantic observation here: The entire parameter space cannot be ensured to be explored. Perhaps replace by something like: ”we broadly explore the parameter space”? **Fixed.** 2. Page 4. “Invented in the early 1950’s, MCMC was originally used in physics to describe equilibrium between the liquid and gas phases of a chemical.” I recommend a citation here, for readers interested in the history of the method itself.

**Good point, we are now citing Metropolis et al. (1953).** 3. Page 5. “. . . a substitution model, which describes the relative rate of change from one character to another, and. . . ”. I suggest to add also: “...and the relative rate of change among character states.

**We have clarified this to: “substitution model, which describes the relative rate of change from one character state to another as well as the frequencies of each character state”, as in most substitution models, the relative rate is based on equilibrium frequencies.** 4. Page 5. “Under this prior, a rate that is very high is believed to be less likely than one that is very [- short] low. **Fixed.** 5. Page 6. “Different estimation methods have been developed to approximate the marginal likelihood, such as path sampling (Baele et al., 2012)[Ref-7] or nested sampling (Russel et al., 2018), but they remain expensive”. Even though, technically, stepping stones sampling is a kind of path sampling procedure, I would specifically

 mention stepping stones separately here as well, since is the most widely used method for estimate marginal likelihoods. Also, in the same sentence, I consider that it would be informative to indicate the term “marginal likelihood” is synonymous with the other term more consistently used in the paper, marginal probability.

**Thanks, we have changed the wording to now mention stepping stone sampling as a popular type of path sampling, and clarify that marginal probability and marginal likelihood are synonymous when introducing the concepts.** 6. Page 7. “. . . We typically record the state of the chain with a frequency that results in a minimum of 10,000 posterior samples.”. But probably less than that, if we perform many moves per generation, as in RevBayes?

**We have edited this section to make it clearer that the number of recorded samples is due to space and memory constraints, and that the sampling frequency will indeed be dependent on the specific software used.** 7. Page 7. “Another important feature of phylogenies is whether they are dated, i.e., whether their branch lengths are expressed in units of genetic/morphological distance or in units of time.” and “Thus we mainly target this article at analyses which include a molecular/morphological clock. . . ”. **Fixed.** 8. Page 7. Estimating a dated phylogeny requires a model for the molecular or morphological clock, a model of lineage diversification, as well as time information to calibrate the tree.

**We have edited this section to mention diversification models, but note that dated phylogenies can also be estimated without such a model (e.g. assuming a uniform prior on topologies and some continuous distribution on the branch lengths).** 9. Page 7. Thus, Scaling moves the components designed to advance the chain and are a core part of any MCMC inference software. **Fixed.** 10. Page 10. “In practice, however, character data is not available or limited for most groups. . . ” As an alternative here, continuous morphological characters could be used in total-evidence analyses (e.g., Álvarez-Carretero et al. (2019)[Ref-1], Zhang et al. (2021)[Ref-2]in press). These could be more readily available for some taxonomic groups, although the performance of including these characters need to be carefully considered (Varón-González et al. (2020)[Ref-3]. **Added.** 11. Page 12. 5.1 Inference technical setup. Here I missed some mentioning of MCMCMC and the use of heated chains or adjusting chain temperature values to try to improve convergence. I guess this is a central topic in the current subject, that should be briefly mentioned by the authors.

**Thank you for pointing out this missing topic. We have now added a section on MCMCMC.** 12. Page 12.” If an operator is missing. . . ”. Maybe the authors could emphasize here that this is a more relevant issue (as far as I can see) in RevBayes, in which we are ”freer” to customize the inclusion of operators (or anything else, basically!).

**We now mention that this issue is particularly relevant for users of RevBayes.** 13. Page 14. “..and with added constraints such as [ - monophyletic] subclades.”. Clades (or subclades) are monophyletic by definition.

**We have clarified this sentence.** 14. Page 15. 5.2.1 Priors. Here I missed some advice on avoiding the use of improper priors, as in some of the Beast2 default settings. These improper priors can also lead to convergence issues sometimes.

**We have added advice on improper priors.** 15. Page 18. ” . . . partition morphological character data”. Although only weakly related to the main theme of the paper, I think other methods of morphological data partitioning and what we know about their performances (which is little, if compared to dna. . . ) could be briefly mentioned here, to give a broader picture to the reader (e.g., Clarke & Middleton (2008)[Ref-4], Rosa et al. (2019)[Ref-5], Casali et al. (2023)[Ref-6]). **Added.**
